# Gene mapping and development of molecular markers for thousand-grain weight in rye based on bulked segregant analysis

**DOI:** 10.7717/peerj.20811

**Published:** 2026-02-12

**Authors:** Lai Wei, Zhenbo Zhai, Yunjie Yang, Yanping Yang, Yonghe Che

**Affiliations:** 1 Hebei Key Laboratory of Crop Stress Biology, Qinhuangdao, Hebei, China; 2 College of Agronomy and Biotechnology, Hebei Normal University of Science and Technology, Qinhuangdao, He Bei, China

**Keywords:** Secale L., BSA-seq, Thousand-grain weight, CP hybrid population, KASP marker

## Abstract

Xinjiang wild rye (*Secale cereale* subsp. *segetale*) is a wild rye species that was discovered in Xinjiang Province of China in the 20th century. It is a relatively understudied cereal crop within the *Poaceae* family, and there is currently a lack of research on its yield-related traits. Mapping genes controlling thousand-grain weight (TGW) is crucial for developing high-yielding rye cultivars. In this study, bulk segregant analysis sequencing (BSA-seq) was performed on a cross-pollinated (CP) hybrid population derived from cultivated rye (Z837) and Xinjiang wild rye (90R13) to identify genomic regions associated with TGW. Molecular markers were then developed in the region of the initially mapped genes to further localize the TGW gene. BSA-seq analysis identified 10 candidate regions containing 68 single nucleotide polymorphism (SNP) loci across the seven chromosomes and unassembled chromosomal segments of rye. Molecular markers were designed for these loci and PCR-based validation was performed, yielding six high-quality Kompetitive allele-specific PCR (KASP) markers and one simple sequence repeat (SSR) marker. After evaluating amplification efficiency, a single KASP marker, *TGW-16*, proved highly effective for selecting germplasm with superior TGW. Functional annotation of the candidate gene *ScWN7R01G304400* (linked to *TGW-16*) revealed that it encodes a protein containing an RNA recognition motif (RRM1) domain. These findings establish both genetic resources and methodological frameworks for marker-assisted breeding in rye, laying a solid foundation for cultivating elite varieties with optimized TGW performance.

## Introduction

Rye (*Secale* L., 2*n* = 2*x* = 14), a diploid species within the *Triticeae* subtribe (*Poaceae*), is a close relative of wheat (Sun et al., 2022). A unique type of wild rye, Xinjiang wild rye (*Secale cereale* subsp. *segetale*), was discovered in the Xinjiang Province of China during the 20th century (Che et al., 2016). Its outstanding advantages, such as resistance to powdery mildew and rust, strong drought resistance, cold resistance, and strong tillering ability, could be used as excellent genetic resources for crop improvement. Based on morphological traits including tillering, plant height, spike morphology, and grain size, Xinjiang wild rye exhibits substantial genetic diversity (Che et al., 2024, 2023).

Grain yield, a key breeding target in *Poaceae* crops, is determined by three quantitative traits: productive tiller number, grains per spike, and thousand grain weight (TGW) (Madhukar, Kumar & Dashora, 2020). Consequently, mapping TGW-associated loci and identifying candidate genes in rye are valuable both theoretically and practically for improving rye productivity and advancing *Triticeae* crop breeding. TGW is a polygenic trait influenced by multiple genes (Mackay, Stone & Ayroles, 2009). These genes collectively regulate grain morphological characteristics including length, width, and shape (Guan et al., 2020; Wang et al., 2019; Kumar et al., 2016). In rice which is used as a model system for *Poaceae* research (Zheng et al., 2022), studies have identified key genes that regulate grain traits. For instance, *GS3* and *GS5* control grain shape, while *GW2*, *GW5*, and *GW8* are major contributors to grain weight. The *GS3* gene, which encodes a transmembrane protein, was characterized as a negative regulator of grain size (Huang et al., 2013; Shomura et al., 2008). *GS3* orthologs function similarly in maize and wheat (Li et al., 2010; Zhang et al., 2014). Conversely, *GS5* encodes a positive regulator of seed size (Li et al., 2011). Loss-of-function mutations in *GW2* increase spikelet hull cell number and grain filling rate, thereby enhancing grain weight (Huang et al., 2013; Song et al., 2007), while *GW5* loss-of-function also increases grain weight (Shomura et al., 2008). *GW8* promotes cell division, and specific promoter mutations enhance its activity (Wang et al., 2012).

Conventional gene mapping approaches for quantitative traits are often time-consuming and costly (Voss-Fels, Cooper & Hayes, 2019). To address these limitations, bulked segregant analysis (BSA) has been increasingly used. As a next generation sequencing-based approach that uses pooled segregating populations, BSA offers an efficient alternative for gene mapping and cloning (Schneeberger & Weigel, 2011). This technique has demonstrated efficacy across various crops. In rapeseed, BSA identified 113 candidate dwarfing genes on chromosome A03 (Huang et al., 2024). In barley, it enabled the characterization of *eIF4E* as conferring strong resistance to both barley yellow mosaic virus and mild mosaic (Shi et al., 2019). Furthermore, in peach, an integrated approach combining genome-wide association studies (GWAS), BSA-seq, and RNA-seq pinpointed a robust genomic region associated with male fertility on chromosome 6 (Huang et al., 2025).

BSA-seq facilitates the identification of single nucleotide polymorphism (SNP) markers and their loci within candidate gene regions. SNP markers are highly valuable for genetic research due to their genomic stability, dense distribution, and capacity for high-throughput genotyping. Researchers conducted experiments using 276 winter rapeseed hybrids and identified 13,116 SNP markers, of which 26 significant SNP loci were associated with yield traits (Bocianowski et al., 2025). In rye, researchers performed GWAS on 526 hybrid rye cultivars evaluated across 19 environments over 2 years; through cross validation of marker-trait associations, they identified 38 yield-associated SNPs, including 13 nonsynonymous SNPs in protein coding sequences that were physically mapped to the Lo7 reference genome (Dörthe et al., 2021; Bauer et al., 2017).

Molecular markers are indispensable for genetic linkage analysis, gene mapping, genome assembly, and breeding. Among these, simple sequence repeat (SSR), SNP, Kompetitive allele-specific PCR (KASP), and AFLP (amplified fragment length polymorphism) markers are widely used. The KASP marker system uses fluorophore-labeled biallelic discrimination at target polymorphic sites (Wei et al., 2020). Its flexibility, accuracy, high throughput capacity, and cost effectiveness have led to widespread adoption in gene mapping, genetic linkage map construction, and population genetics studies. KASP markers have demonstrated broad utility across diverse crop species. In southern poplar, researchers developed KASP markers for five SNP loci within candidate quantitative trait locus (QTL) regions, successfully identifying three markers significantly associated with leaf area and plant height (Liu et al., 2025). For castor bean, two KASP markers tightly linked to *Fusarium* wilt resistance were established, enabling marker-assisted breeding (Sathishkumar et al., 2025). In wheat, KASP markers for resistance to stripe rust and leaf rust resistance were validated across cultivars from multiple countries (Bansal et al., 2021). Rice variety identification was achieved using 48 KASP primer pairs, enabling efficient classification of numerous cultivars (Tang et al., 2022).

As a distinct species in the *Poaceae* family, rye exhibits outcrossing and self-incompatibility characteristics. These have brought significant challenges for genetic studies. The application of mapping the double pseudo-crossing (cross-pollinated, CP) hybrid population has contributed to discovering effective QTLs in the self-incompatibility population (Shi et al., 2020; Zhou et al., 2018; Zhu et al., 2015). The current study used cultivated rye (*S. cereale* L.), Xinjiang wild rye (*S. cereale* subsp. *segetale*,), and their CP hybrid progeny (C_1_, C_2_) as plant materials. BSA-seq was used to map genomic regions harboring TGW-associated loci and identify linked polymorphic sites (SNPs). These candidate SNPs were converted into molecular markers, ultimately identifying genes exhibiting strong association with TGW variation. These results provide a foundation for breeding high-yielding rye cultivars *via* marker-assisted selection.

## Materials and Methods

### Materials

The plant materials used in this study were provided and maintained by Hebei Normal University of Science and Technology. All field experiments were conducted under conventional management at the University’s Agricultural Experimental Station in Changli, Hebei Province, with hill-drop sowing every October (30 cm row spacing × 20 cm plant spacing), standard irrigation/fertilization management, and trait investigations performed the following August.

The CP Hybrid seeds (F_1_) were generated by crossing Z837 (cultivated rye) with 90R13 (Xinjiang wild rye). The F_1_ hybrid population (C_1_), consisting of 376 lines, was cultivated in 2020 for BSA. Subsequently, the F_2_ population (C_2_), comprising 322 lines, was grown in 2021 for validation studies.

### Phenotypic investigation and extreme pool sequencing

Phenotypic characterization was performed on all plants from both C_1_ and C_2_ populations after rye maturation and drying. These traits included productive tiller number per plant, yield per plant, spike length, spike width, grain number per spike, grain weight per spike, and TGW. Spike-related traits were assessed by randomly selecting five spikes per plant, with trait measurements averaged across replicates. For TGW determination, all seeds from individual plants were pooled, followed by random sampling of 1,000 seeds for triplicate measurements. The mean value was calculated for subsequent analysis.

Twenty plants each exhibiting extreme TGW phenotypes from the C_1_ population, along with parental controls, were selected for DNA extraction and pooled library construction (performed by Biomarker Technologies Corporation, Beijing). Genomic DNA was extracted, and its purity and integrity were assessed using agarose gel electrophoresis and spectrophotometry (OD _260/280_ ratio). Following quantification, DNA was randomly fragmented (−350 bp), subjected to end repair and A-tailing, and ligated to adapters. After purification and amplification, libraries were constructed and sequenced on an Illumina HiSeq™ PE150 platform.

### Candidate gene fragment screening

The obtained data were filtered to remove paired reads-containing adapters, single end reads with over 10% N content, or paired reads where more than half of the bases in either read were of low quality. High-quality reads were aligned to the Weining rye reference genome using BWA (Li et al., 2021; Li & Durbin, 2009). PCR duplicates were removed using SAMtools (Li et al., 2009). SNPs in the samples were subsequently detected and filtered using GATK 3.8 (McKenna et al., 2010). By sequentially mapping the SNP-index and InDel-index of progeny along the chromosomes, significant variations in these indices were observed across different genomic loci. The difference in SNP-index and InDel-index between the two extreme progeny pools was calculated as Δ (All-index) = All-index (high TGW)-All-index (low TGW). The average Δ (All-index) value within each sliding window was used to determine the final candidate regions (Takagi et al., 2013).

### Analysis of SNP frequency in progeny and identification of minor-effect genes

For the SNP loci obtained by BSA-seq, and using the maternal Xinjiang wild rye 90R13 as the reference parent, the SNP-index (ranging from 0 for identical SNPs to 1 for completely different SNPs) was calculated for polymorphic markers in both progeny populations. Only non-missing loci with SNP-index values >0.3 and sequencing depth >7 were retained for subsequent analysis.

The Δ (SNP-index) distribution between the two progeny populations was calculated using a 1 Mb sliding window with a 1 kb step size. Candidate regions associated with the target trait were identified through 1,000 permutation tests, with windows exceeding the 95% and 99% confidence thresholds considered significant.

To further investigate the contribution of minor effect QTLs to TGW, genome-wide screening was performed to select SNPs or InDels showing significant differences in their respective indices between the two progeny populations. These variants were annotated using ANNOVAR (Wang, Li & Hakonarson, 2010), with priority given to mutations predicted to affect gene expression regulation. The corresponding genes harboring these regulatory mutations were identified as candidate genes for TGW.

### Molecular marker development

Based on BSA-seq results, molecular markers were designed targeting preliminary candidate SNP loci associated with TGW. PCR amplification was performed using DNA from C_1_ and C_2_ populations as templates. For SSR markers, PCR products were analyzed by gel electrophoresis. For KASP markers, the CFX Connect Real-Time PCR Detection System was used for PCR amplification and fluorescence intensity determination. Following amplification, genotyping was performed. The genotyping results were combined with phenotypic data and analyzed using t-test to evaluate significant associations between genotypes and TGW phenotypes.

The genome assembly and annotation files of Weining rye were downloaded from the NCBI database. Subsequently, molecular markers with statistically significant associations (*P* < 0.05) were analyzed, and the corresponding SNP loci were used as reference points to identify nearby genes within the Weining rye genome. The transcriptome data of rye were downloaded from the WheatOmics database, and the expression patterns of these genes across various tissues under non-stress growth conditions were analyzed. Genes with relatively high expression levels were selected as candidate genes. Subsequently, the molecular markers corresponding to these candidate genes were validated across different rye populations to obtain the final set of molecular markers and candidate genes.

### Candidate gene analysis

Using the annotated genes of Weining rye as a reference. The coding sequences (CDS) and protein sequences of the candidate genes were obtained using TBtools (Chen et al., 2020). Functional annotation was performed *via* eggNOG-mapper (Carlos et al., 2021), and further investigation determined whether these proteins affect TGW traits in *Poaceae* species (wheat, rice, *etc*.). This cross-species analysis identified genes controlling TGW in Xinjiang wild rye. Synteny analysis was then conducted between these confirmed genes and their orthologs in wheat/rice using TBtools to examine whether the syntenic genes have documented effects on TGW.

## Results

### Construction of extreme pool

**Figure 1:**
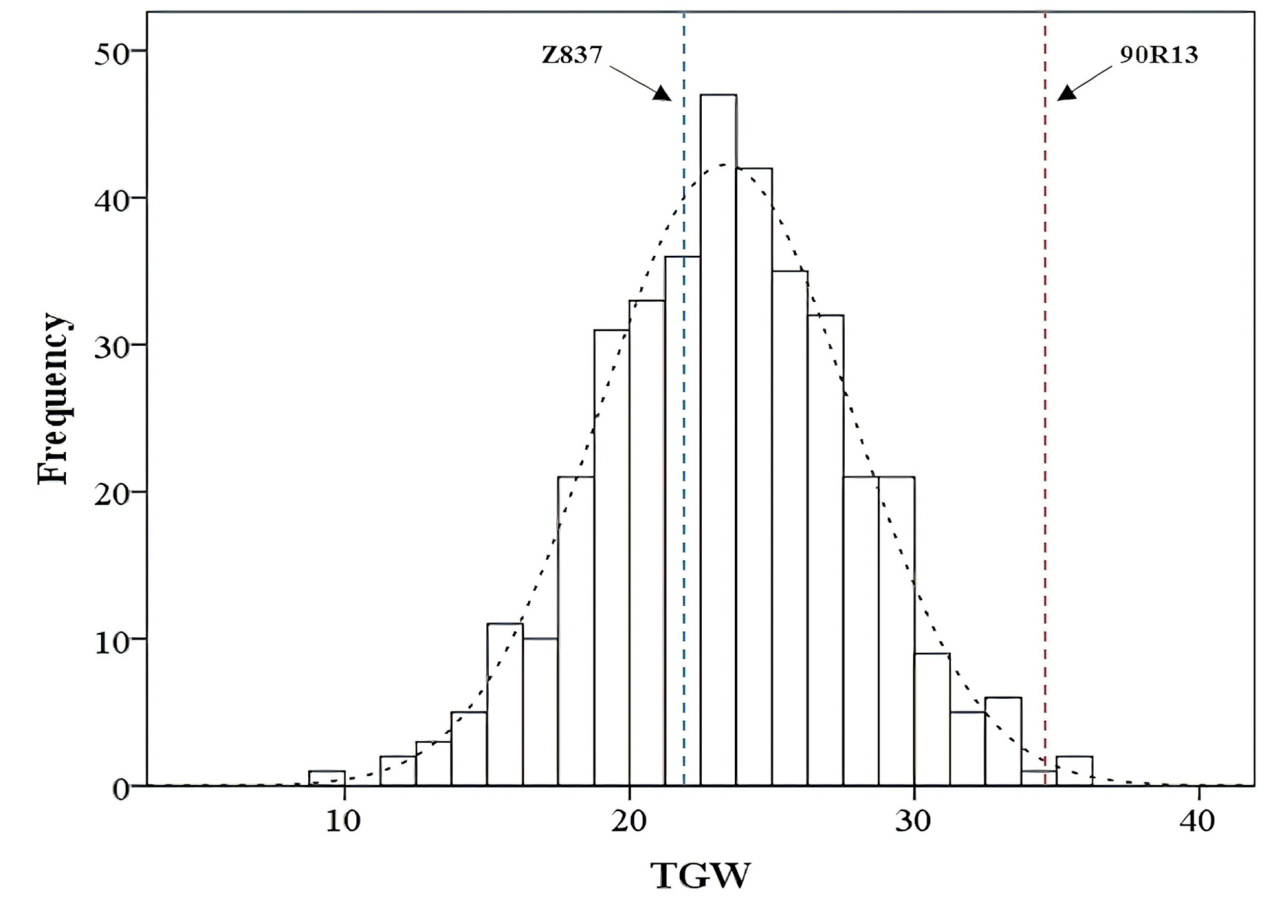
Frequency distribution of the TGW traits in the C_1_ population. The X-axis represents the TGW phenotypic data (unit: g), while the Y-axis indicates the number of rye plants falling within each corresponding trait range.

Phenotypic analysis of the C_1_ population revealed that the TGW data for individual plants followed a normal distribution (Fig. 1). Based on this distribution, 20 extreme individuals each with the highest and lowest TGW values were selected to construct two bulks (Table 1). Phenotypic data comparison confirmed a significant difference between these two extreme bulks.

**Table 1:** Phenotypic statistics of the two extreme TGW bulks.

Metrics	H-pool	L-pool
Standard deviation	1.456	1.607
Variance	2.119	2.583
Deviation	1.052	−1.03
Kurtosis	0.669	1.073
Extreme difference	5.39	6.19
Max/(g)	35.92	16.16
Min/(g)	30.53	9.97
Mean/(g)	32.3097	14.2577

### BSA sequencing results

Sequencing was performed for both parental lines and the two extreme pools. As shown in Table 2, the experiment generated a total of 438.1 GB of raw sequencing data, of which 428.2 GB remained after quality filtering for downstream analysis. The filtered sequencing data showed high quality, with Q20 ≥ 96.88% and Q30 ≥ 91.82%. The GC content was between 45.66% and 46.1%. From these data, 1,602,850 polymorphic marker loci were identified.

**Table 2:** Sequence data summary for parents and bulked H-pool and L-pool.

Sample	Clean base (bp)	Mapped reads	GC content (%)	Mapping rate (%)	Q20 (%)	Q30 (%)	Average depth (X)
M	71,109,292,200	474,061,948	46.1	98.68	96.98	91.97	8.55
P	69,990,140,400	466,600,936	45.95	98.76	96.88	91.82	8.3
L-pool	140,384,085,600	978,255,588	45.76	98.52	96.89	91.85	14.68
H-pool	146,738,338,200	935,893,904	45.66	97.85	96.93	91.92	15.31

**Note:**

Sample: the name of the sample; Clean Base: the effective data volume after filtering, the number of sequenced sequences after filtering multiplied by the length of sequenced sequences in bp; Q20, Q30: the percentage of the bases with Phred value greater than 20 or 30; GC Content: the percentage of the sum of bases G and C in the total bases; Mapped reads: the number of reads matched to the reference (including single-end matching and double-end matching); Mapping rate: the matching rate, the number of reads matched to the reference genome. Mapped reads: the number of reads matched to the reference (including single-end matching and double-end matching); Mapping rate: the matching rate, the number of reads matched to the reference genome divided by the number of reads in the valid sequencing data; Average depth: the average sequencing depth, the total number of bases matched to the reference genome divided by the total number of bases in the genome, Average depth: average sequencing depth, the total number of bases matched to the reference genome divided by the genome size.

### Analysis of candidate genomic regions associated with TGW Traits

**Figure 2:**
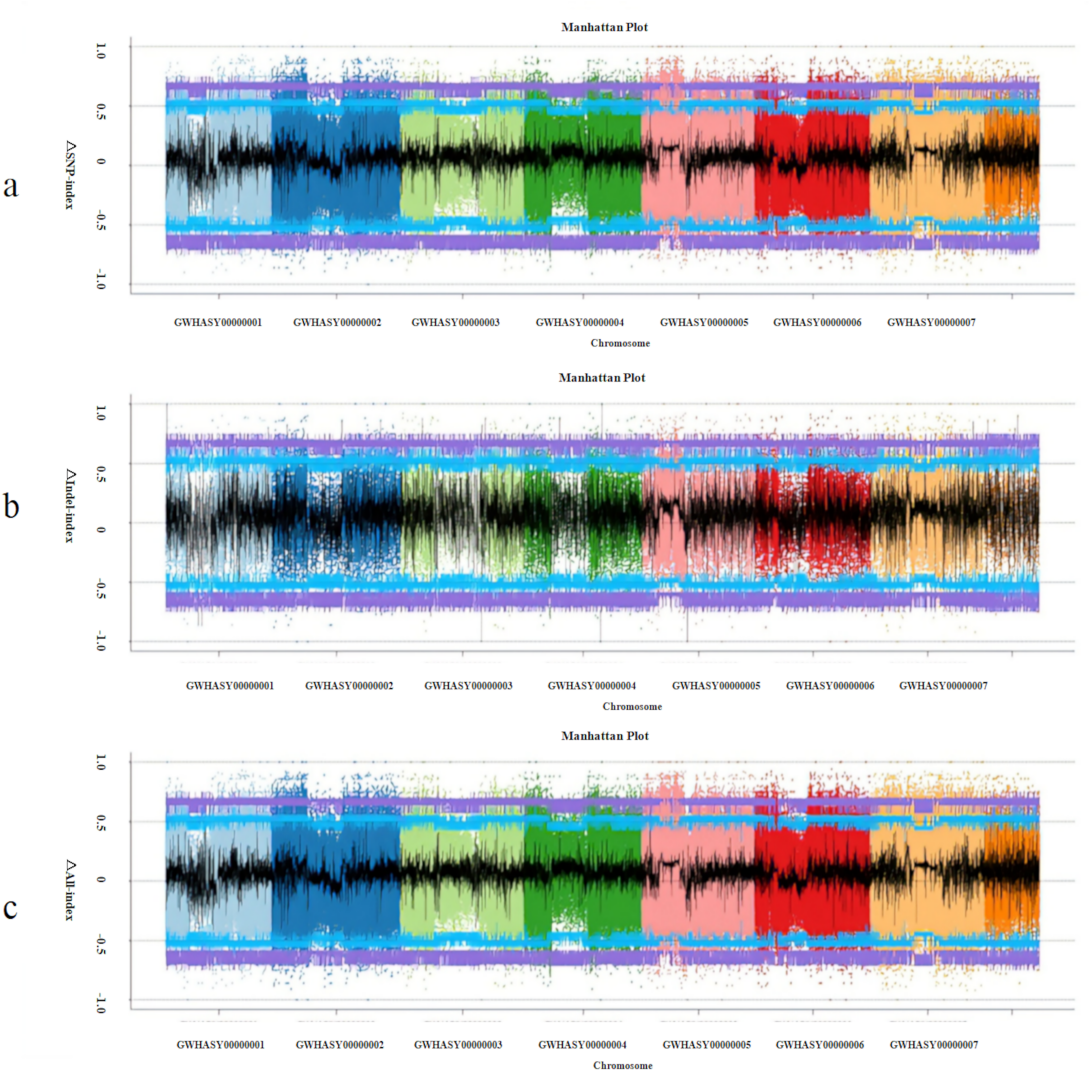
Distribution of polymorphic loci on chromosomes. (A) Distribution of two offspring ΔSNP-index on chromosomes. (B) Distribution of two offspring ΔIndel-index on chromosomes. (C) Distribution of two offspring ΔAll-index on chromosomes. Each data point in the figure represents an individual SNP or InDel locus, with its horizontal coordinate indicating the midpoint of the genomic window containing the variant and its vertical coordinate showing the ΔAll-index difference between the two progeny pools. Distinct colors differentiate chromosomes. The black line denotes the mean ΔAll-index value, while the blue and purple lines represent the 95% and 99% significance thresholds, respectively. Genomic regions where the mean line exceeds these threshold lines warrant particular attention.

The distributions of the ΔSNP-index, ΔInDel-index, and Δ(All-index) across rye chromosomes are shown in Fig. 2. Figures 2A, 2B, and 2C present the chromosomal distributions of ΔSNP-index (34,087,464 loci), ΔInDel-index (2,414,323 loci), and Δ(All-index) (36,501,787 loci) which combines SNP and InDel variant, respectively, in the two progeny populations. Through data analysis at the 95% confidence level, 10 candidate gene segments were identified across the seven rye chromosomes and unnamed fragments. Their distribution was as follows: three on chromosome 1; one each on chromosomes 3, 4, and 5; and four on unnamed chromosomal fragments (sequences not assigned to specific chromosomes in the reference genome) (Table 3). Among these, the *qTGW-5-1* interval exceeded the threshold at the 99% confidence level.

**Table 3:** Rye TGW candidate gene region information.

Location	Chromosome	Start and End
*qTGW-1-1*	Chr.1	295,623,001 295,798,000
*qTGW-1-2*	Chr.1	349,326,001 349,379,000
*qTGW-1-3*	Chr.1	357,667,001 357,852,000
*qTGW-3-1*	Chr.3	1,094,711,001 1094773000
*qTGW-4-1*	Chr.4	313,824,001 314,483,000
*qTGW-5-1*	Chr.5	68,480,001 68,485,000
*qTGW-Un-1*	Chr.8	181,169,001 181,571,000
*qTGW-Un-2*	Chr.8	291,706,001 291,734,000
*qTGW-Un-3*	Chr.8	331,785,001 331,844,000
*qTGW-Un-4*	Chr.8	417,453,001 417,855,000

### Screening results of minor-effect candidate genes

Based on genotyping results, polymorphic markers were identified between the two parental lines, with a total of 1,602,850 markers detected. Following rigorous screening, a total of 68 candidate SNP loci were ultimately identified (Table 4). The majority of SNP loci were located in the upstream regions of genes.

**Table 4:** Annotation of candidate SNPs loci.

Category	Number of SNPs
Downstream	1
Frameshift insertion	1
Nonsynonymous	13
Upstream	38
UTR3	8
UTR5	7
Total	68

### Molecular marker development

**Figure 3:**
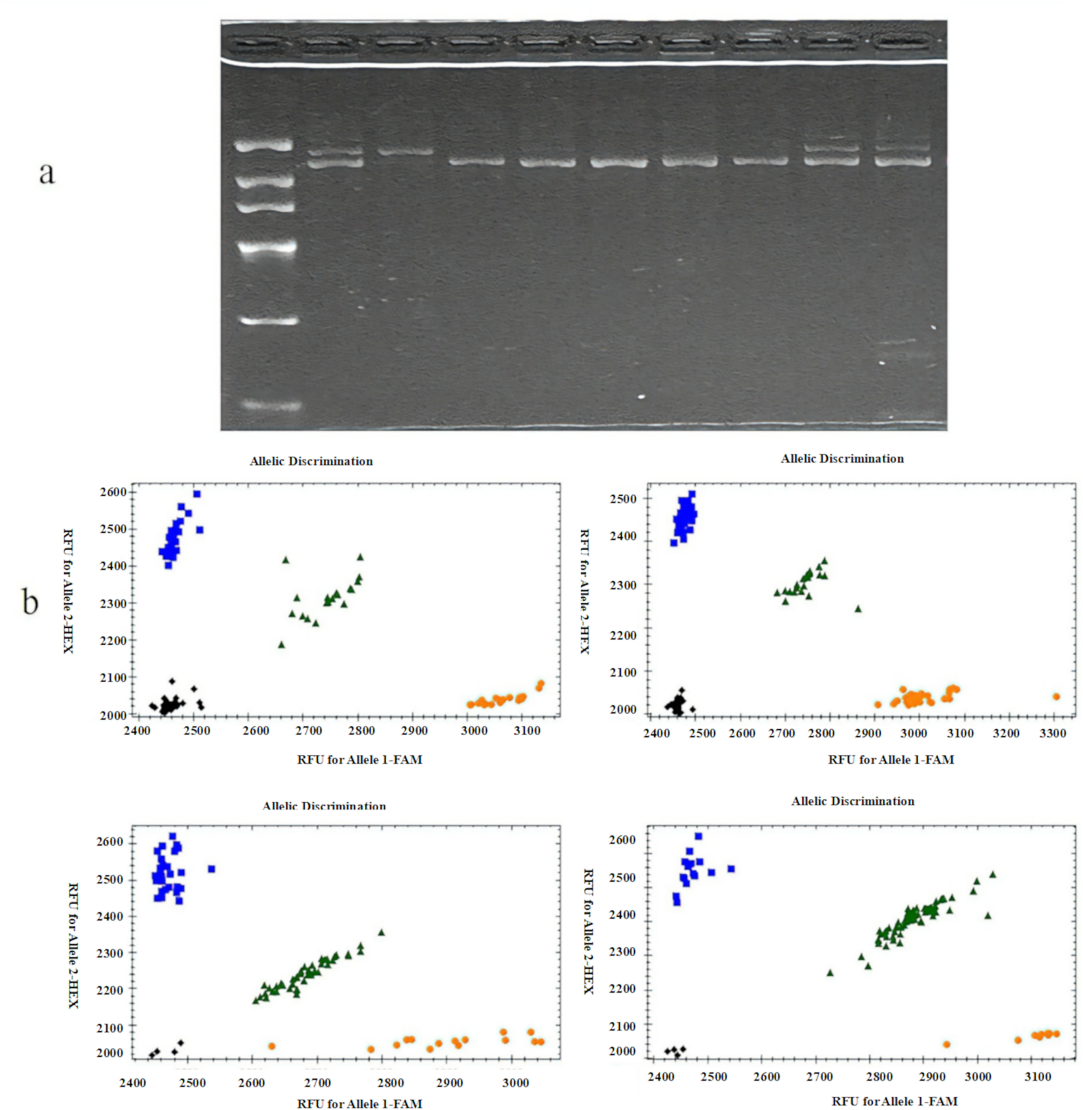
Representative experimental results. (A) Results of *SSR25* in the C_1_ population. Marker is 100–2,000 bp; length of the target band is 1,478 bp; lane 1–9 is the target gene band; lane 2 is allele 1; lanes 3, 4, 5, 6, and 7 are allele 2; lane 1, 8 and 9 are heterozygous. (B) Genotyping plot of KASP markers in C_1_ population. X-axis, Allele 1, reported by FAM-type fluorescence; Y-axis, Allele 2, reported by HEX-type fluorescence; blue dots stand for homozygous allele group 1; orange dots stand for homozygous allele group 2; green dots stand for heterozygous alleles; black dots stand for no call.

Using the 68 polymorphic loci as anchors, linked SSR sequences were identified and corresponding primers were designed (designated *SSR-X*). These primers were tested for polymorphism detection in the C_1_ population, resulting in one successfully genotyped primer pair (*SSR-25*). T-test analysis revealed a significant effect (*P* < 0.05) of *SSR-25* genotype on TGW. Detailed primer significance levels and amplification efficiencies are shown in Table 5, and representative experimental results are shown in Fig. 3.

**Table 5:** Primer information of markers.

Primer name	Primer type	Primer sequence	Amplification rate	*P*
SSR-25	Forward	ACGATGGCGTCCCTACA	87.50%	0.019
	Reverse	GCTTGCGTTAATGGTGCT		
TGW-3	Forward 1	GAAGGTGACCAAGTTCATGCTagactgagatgcaaacaagacgt	76.61%	0.023
	Forward 2	GAAGGTCGGAGTCAACGGATTagactgagatgcaaacaagacgtC		
	Reverse	TGCGAGATCGGACGTTCCT		
TGW-8	Forward 1	GAAGGTGACCAAGTTCATGCTctccccatctggcacgcC	100.00%	0.044
	Forward 2	GAAGGTCGGAGTCAACGGATTctccccatctggcacgcA		
	Reverse	ATGTATGTGTGGGTGCAGCT		
TGW-15	Forward 1	GAAGGTGACCAAGTTCATGCTcgctggtcgatgtggagG	77.69%	0.008
	Forward 2	GAAGGTCGGAGTCAACGGATTcgctggtcgatgtggagC		
	Reverse	GTGCTGTCTACGAGGTGGAG		
TGW-16	Forward 1	GAAGGTGACCAAGTTCATGCTtcgttcagcagcatacctttttC	98.40%	0.0016
	Forward 2	GAAGGTCGGAGTCAACGGATTtcgttcagcagcatacctttttT		
	Reverse	AGCATGGCACCTTGGATGAT		
TGW-25	Forward 1	GAAGGTGACCAAGTTCATGCTaccttgttggagatgctagagaG	99.73%	0.001
	Forward 2	GAAGGTCGGAGTCAACGGATTaccttgttggagatgctagagaC		
	Reverse	AAACTCACTGCCTCATCGCG		
TGW-36	Forward 1	GAAGGTGACCAAGTTCATGCTcgaccgatttgcccagacaC	99.19%	0.102
	Forward 2	GAAGGTCGGAGTCAACGGATTcgaccgatttgcccagacaT		
	Reverse	TGACAACCAACACGCTTCGA		
TGW-49	Forward 1	GAAGGTGACCAAGTTCATGCTaaggaacagaggggagagcA	99.73%	0.0001
	Forward 2	GAAGGTCGGAGTCAACGGATTaaggaacagaggggagagcT		
	Reverse	AGGAGGACGACCAAGAGGAG		
TGW-52	Forward 1	GAAGGTGACCAAGTTCATGCTagatgaagatcaagcactactcG	99.19%	0.508
	Forward 2	GAAGGTCGGAGTCAACGGATTagatgaagatcaagcactactcA		
	Reverse	CTACTGCGGGTTCTCGGTG		

Based on the 68 polymorphic loci, KASP primers were designed and screened using DNA templates from extreme phenotype samples of the C_1_ population. Eight polymorphic primer pairs were successfully developed and designated as *TGW-X* (where X represents numerical identifiers). These eight KASP markers were then applied to genotype the entire C_1_ population. After excluding heterozygous individuals, t-tests were performed combining genotypic and phenotypic data. The analysis revealed that *TGW-15*, *TGW-16*, *TGW-25*, and *TGW-49* showed highly significant associations (*P* < 0.01), while *TGW-8* demonstrated significant association (*P* < 0.05). Notably, *TGW-25* and *SSR-25* were able to genotype the same polymorphic locus. Complete statistical results for all primers and amplification efficiencies are provided in Table 5, and representative experimental results are shown in Fig. 3. The information regarding SNP loci corresponding to molecular markers is shown in Table 6. This table presents phenotypic data and comparative analysis of SNP loci, enabling the determination of their relative contributions to TGW. Notably, the SNP loci corresponding to *TGW-16* and *TGW-49* demonstrated the highest contribution rates of 30.24% and 12.12%, respectively.

**Table 6:** The information of SNP loci corresponding to molecular markers.

Markers	Trans ID	Chrom	Pos	Ref	TGW data (g)	Alt	TGW data (g)	Rate (%)
TGW-8	*ScWN3R01G177500*	GWHASIY00000003	202370748	C	22.04	A	23.39	6.13
TGW-15	*ScWN6R01G478100*	GWHASIY00000006	882213763	C	24.59	G	22.91	7.33
TGW-16	*ScWN7R01G304400*	GWHASIY00000007	627731665	G	18.29	A	23.82	30.24
TGW-25	*ScWN2R01G320500*	GWHASIY00000002	665760865	G	23.85	C	21.78	9.5
SSR-25								
TGW-49	*ScWN7R01G178400*	GWHASIY00000007	205488809	T	21.61	A	24.23	12.12

**Figure 4:**
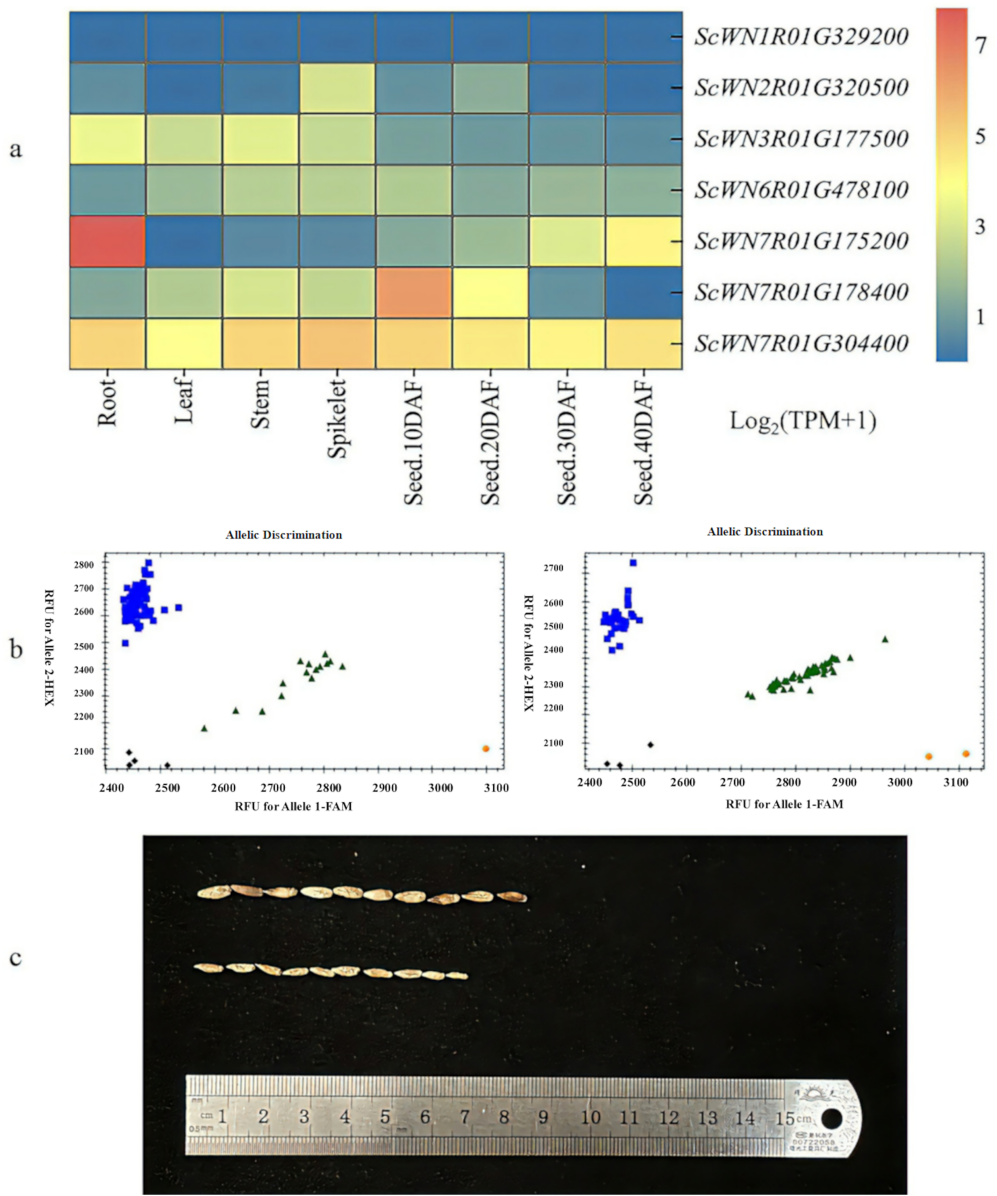
Molecular marker verification. (A) Expression status of seven genes in roots, leaves, stems, spikes, and seeds; red color represents high expression and blue color represents low expression. (B) Genotyping plot of *TGW-16* marker detection in C_2_ population. X-axis, Allele 1, reported by FAM-type fluorescence; Y-axis, Allele 2, reported by HEX-type fluorescence; biue dots stand for homozygous allele group 1; orange dots stand for homozygous allele group 2; green dots stand for heterozygous alleles; black dots stand for no call. (C) Comparison of TGW of plants screened by molecular markers; the upper part is the high TGW grain and same genotype as the parent 90R13; the lower part is the bottom TGW grain and same genotype as the parent Z837.

We analyzed the genes that showed significant associations (*P* < 0.05) with molecular markers, as well as their spatial expression profiles across different rye tissues. The expression levels of these genes were visualized using TBtools (Fig. 4A). The results showed that the gene *ScWN7R01G178400* corresponding to *TGW-15* marker was highly expressed in all tissues, with particularly high expression in developing grains at early stages (10 DAF) followed by a sharp decrease after 20 days. This gene encodes a xyloglucan endotransglycosylase/hydrolase (HET) that regulates the cross-linking strength of the cellulose-xyloglucan network through dual enzymatic activities: hydrolytic cleavage of β-1,4-glycosidic bonds in xyloglucan backbones and transglycosylation of cleaved fragments to other xyloglucan chains. These functions collectively modulate cell wall extensibility and mechanical properties, though no prior studies of *Poaceae* have linked this protein’s activity to TGW variation. Another gene corresponding to the *TGW-16* marker, *ScWN7R01G304400*, showed the highest expression in spikes while also maintaining relatively high expression during grain development. This gene encodes an RNA recognition motif (RRM) domain-containing protein, and previous studies have demonstrated that this protein can influence seed development in rice.

Based on the expression patterns of candidate genes in rye tissues, marker amplification efficiency, and t-test results of molecular markers, *TGW-16* was identified as a promising candidate marker. Validation was performed using 322 lines from the C_2_ population. Initial screening with extreme phenotype progeny confirmed successful genotyping using *TGW-16* primers (Fig. 4B). This was followed by whole population genotyping using the same reaction system and data processing methods as described previously. The amplification rate was 94.1%. T-test analysis of genotypic and phenotypic data demonstrated that *P* = 0.0024, reaching extremely significant levels (*P* < 0.01). The representative experimental data and grain morphology comparison between high and low TGW plants selected by *TGW-16* in the C_2_ population are shown in Fig. 4C.

### Candidate gene analysis

Gene annotation was performed for *TGW-16* and other well-genotyped markers (*SSR-25*, *TGW-3*, *TGW-8*, *TGW-15*, *TGW-25*, and *TGW-49*) with *P* < 0.05. The *TGW-16* associated gene *ScWN7R01G304400* encodes an RNA recognition motif 1 (RRM1) domain, while other genes encode MYB-binding site proteins, F-box family proteins, glycosyl hydrolase family proteins, and xyloglucan endotransglucosylase/hydrolase proteins (Table 7).

**Table 7:** Annotation of Lo7 rye TGW gene and localized gene candidates.

Gene	Description	PFAMs
*SECCE2Rv1G0077160.1*	CobN/Magnesium Chelatase	CobN-Mg_chel, DUF3479
*SECCE2Rv1G0109590.1*	Glycosyl hydrolase family 9	Glyco_hydro_9
*SECCE3Rv1G0163840.1*	Protein gamma response	SAE2
*SECCE3Rv1G0197430.1*	C2H2-type zinc finger	zf-C2H2_6
*SECCE3Rv1G0200810.1*	Chlorophyll A-B binding protein	Chloroa_b-bind
*SECCE4Rv1G0222350.1*	inositol 3-kinase activity	PfkB
*SECCE4Rv1G0280740.1*	Belongs to the cytochrome P450 family	p450
*SECCE7Rv1G0464290.1*	3-ketoacyl-CoA synthase	ACP_syn_III_C, FAE1_CUT1_RppA
*SECCE7Rv1G0481970.1*	Myb-like DNA-binding domain	Myb_DNA-binding
*SECCE7Rv1G0490840.1*	Belongs to the glycosyltransferase 2 family	Cellulose_synt
*SECCE7Rv1G0522470.1*	cytochrome p450	p450
*SECCE7Rv1G0522500.1*	von Willebrand factor (vWF) type A domain	VWA_2, zf-RING_11, zf-RING_2
*ScWN2R01G320500.1*	zpr3 zpr3 (little zipper 3)	–
*ScWN3R01G177500.1*	Myb-like DNA-binding domain	Myb_DNA-binding
*ScWN6R01G478100.1*	–	F-box,F-box-like
*ScWN7R01G178400.1*	Catalyzes xyloglucan endohydrolysis (XEH) and or endotransglycosylation (XET).	Glyco_hydro_16, XET_C
*ScWN7R01G304400.1*	HAT (Half-A-TPR) repeats	Lsm_interact, RRM_1, Suf

**Note:**

SECCE: Gene previously identified to affect TGW in LO7 rye, ScWN: Candidate genes newly associated with TGW variation in this study.

**Figure 5:**
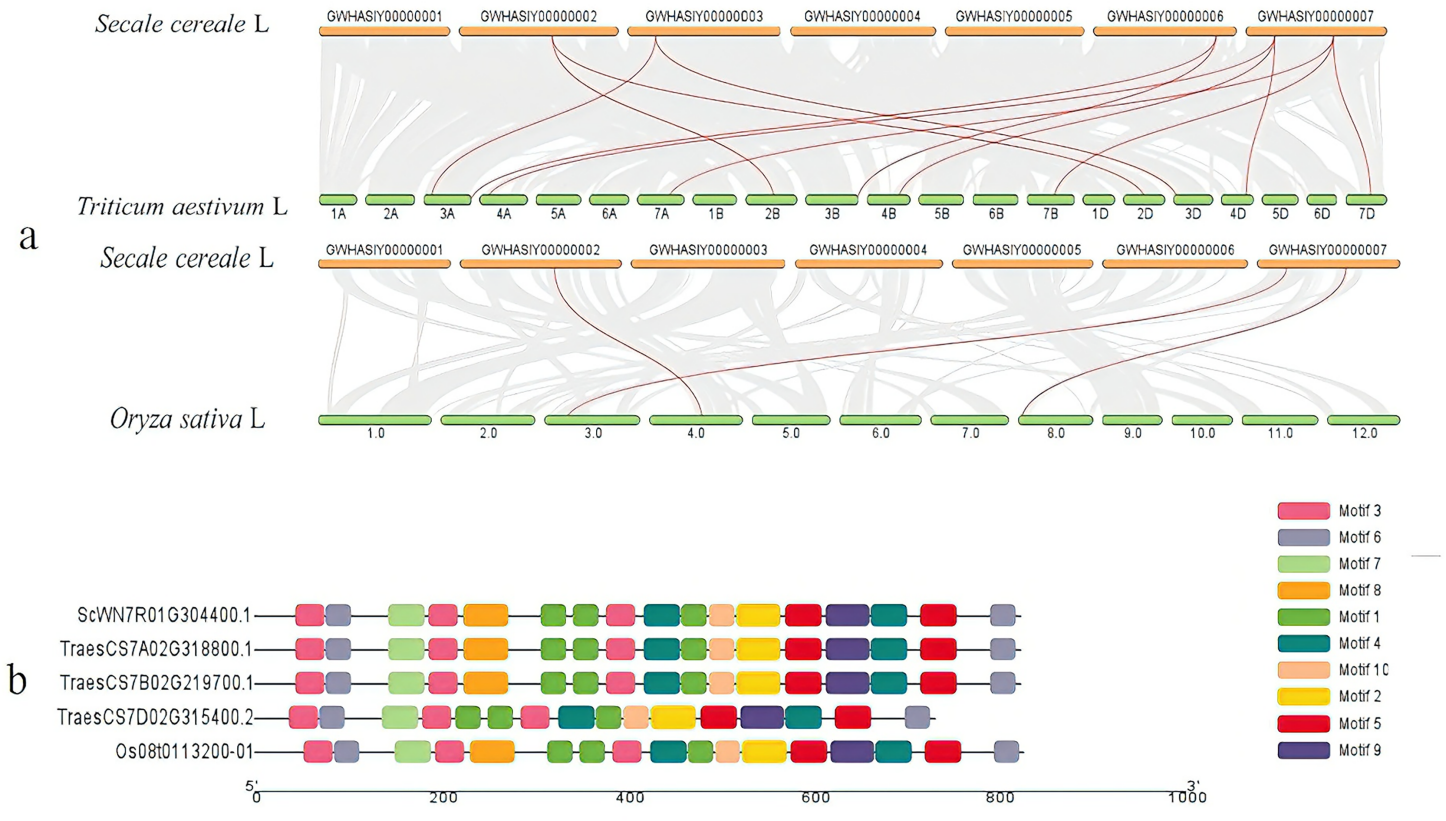
Candidate gene analysis. (A) The collinearity results of rye candidate genes with wheat and rice. Collinearity of rye with rice and wheat genomes. The gray line indicates the collinear relationship between rye and the other two plants, and the red line indicates the collinear relationship between target gene. (B) Protein domain distribution map. The boxes of different colors represent different conserved motifs, which are divided into Motif 1–10.

Collinearity analysis results are shown in Fig. 5A. The analysis revealed that the *TGW-16* associated gene *ScWN7R01G304400* was homologous to *TraesCS7A02G318800*, *TraesCS7B02G219700*, and *TraesCS7D02G315400* in wheat, and to *Os08t0113200* in rice. Other genes also showed collinear relationships: *ScWN2R01G320500* was homologous to *TraesCS2B02G310600*, *TraesCS2D02G291900* and *Os04t0411200*. *ScWN3R01G177500* was homologous to *TraesCS3A02G123700* and *TraesCS3D02G125300*. *ScWN6R01G478100* was homologous to *TraesCS3A02G518400* and *TraesCS3B02G585800*. *ScWN7R01G178400* was homologous to *TraesCS4A02G073300*, *TraesCS4B02G229500*, *TraesCS4D02G230600*, and *Os03t0239000*. The functional annotations of these syntenic genes in wheat and rice were investigated, and there were no reported associations with TGW in the existing literature.

To further investigate the distribution of RRM1 domains in these proteins, conserved motif analysis was performed using the MEME suite for *ScWN7R01G304400* and its homologous genes (*TraesCS7A02G318800*, *TraesCS7B02G219700*, *TraesCS7D02G315400*, and *Os08t0113200*). The results are presented in Fig. 5B, which clearly demonstrates that all examined proteins shared identical functional domains (motif 1–motif 10), indicating strong evolutionary conservation of this protein domain architecture.

## Discussion

### Analysis of yield-related traits in rye

Rye exhibits self-pollination barriers due to its self-incompatibility system (Li et al., 2021), resulting in a genetically diverse background that contributes to extensive phenotypic variation. Systematic evaluation of morphological traits in rye progeny populations, combined with comparative analysis of agronomic trait phenotypes between parental and offspring lines, enables effective identification and utilization of superior genetic traits. This approach provides valuable genetic resources for both trait mapping in rye and genetic improvement in cereal breeding programs. This study conducted morphological characterization and analysis of rye hybrid populations, revealing highly significant correlations among most traits.

For example, the increases in TGW were consistently associated with concurrent increases in spike weight, kernel length, kernel width, and kernel weight per spike (Table 8). These findings indicate that when selecting individual plants with superior target traits, one must consider both the primary target traits and their positively correlated agronomic characteristics. The hybrid populations exhibited substantial genetic diversity across agronomic traits, suggesting that targeted selection can facilitate breeding improved cultivars while establishing a foundation for identifying elite genetic resources.

**Table 8:** Correlation analysis of TGW and other agronomic traits in the C_1_ population.

Traits	PH	ATN	BY	PY	SL	SWd	SWg	GL	GW	KNS	KWS	SNS
Relevance	0.34**	0.28**	0.37**	0.40**	0.32**	0.50**	0.53**	0.51**	0.68**	−0.11*	0.54**	−0.02
*P*	0.000	0.000	0.000	0.000	0.000	0.000	0.000	0.000	0.000	0.032	0.000	0.972

**Notes:**

PH represents plant height, ATN represents available tiller number, BY represents biological yield, PY represents plant yield, SL represents spike length, SWd represents spike width, SWg represents spike weight, GL represents grain length, GW represents grain width, KNS represents kernel number per spike, KWS represents kernel weight per spike, SNS represents spikelet number per spike.

* indicates significant correlation (*P* < 0.05).

** indicates highly significant correlation (*P* < 0.01).

### Preliminary mapping analysis of TGW genes

This study used a hybrid population derived from crosses between wild and cultivated rye as experimental material. Phenotypic screening identified individuals exhibiting extreme TGW phenotypes, which were subsequently pooled to construct two bulks for BSA-seq. Preliminary mapping of TGW associated loci was performed using the Weining rye genome as reference (Li et al., 2021), combined with parental genome resequencing data. A total of six putative quantitative trait loci distributed across chromosomes 1R, 3R, 4R, and 5R were identified. It should be noted that four genomic scaffolds in the Weining v1.0 assembly remain unanchored to specific chromosomes and were designated as “Un” chromosomal segments.

### Analysis of TGW candidate genes in rye

Rye is a relatively understudied cereal crop within the *Poaceae* family, and there is current a lack of research on its yield-related traits, particularly TGW. This study screened multiple SNP loci and identified 68 candidate SNPs associated with TGW, which were then used to develop molecular markers for TGW related gene mapping. Genotyping analysis using the Xinjiang wild rye population C_1_ identified six KASP markers and one SSR marker with reliable genotyping performance. Validation of the well-performing *TGW-16* marker across different rye populations confirmed its utility as a molecular marker for future rye breeding programs. Subsequent genotyping and phenotypic comparison of the SNP locus associated with this molecular marker revealed a 30.24% phenotypic variation in TGW.

Functional annotation of the *ScWN7R01G304400* gene corresponding to the *TGW-16* marker revealed that it encodes an RNA recognition motif (RRM1) domain. RRM1 protein has a regulatory role in grain development. For example, the rice SOE (SUPPRESSOR OF OsEIN2) protein containing an RRM domain maintains mRNA stability of the DNA demethylase gene *DNG701* by regulating spliceosome activity, thereby preserving promoter demethylation levels and influencing grain development and epigenetic modifications (Liu et al., 2020). SOE gene mutants significantly increase grain size and yield per plant. The Fudan University research team unexpectedly discovered that overexpression of the RRM1 domain in the rice flowering gene FCA enlarges all cell types (including grain cells), ultimately increasing grain volume and weight by 73% (Zhao et al., 2024). The exogenous RRM1 competes with the endogenous FCA protein’s RRM1 domain, interfering with normal FCA function and activating a suppressed “large-grain formation program.” Additionally, *OsGRF4* (*GS2*) contains an RRM domain that binds *miRNA396* to relieve target gene suppression, promoting cell proliferation and grain enlargement (Hong et al., 2007). Collinearity analysis confirmed *ScWN7R01G304400* orthology with genes in wheat chromosome 7 and rice chromosome 8. Conservative sequence analysis of this gene and its syntenic counterparts revealed high conservation of this protein domain.

The candidate genes (*ScWN3R01G177500*, *ScWN6R01G478100*, *ScWN2R01G320500*, and *ScWN7R01G178400*) corresponding to well-genotyped molecular markers were compared with known genes affecting TGW in *LO7* rye (Dörthe et al., 2021; Bauer et al., 2017), revealing no positional overlap (Table 7). Collinearity analysis between these genes and their orthologs in wheat and rice identified several syntenic genes (Fig. 5A). Functional annotation of these syntenic genes in wheat and rice indicated they remain uncharacterized in current literature. Functional annotation of the genes revealed that they encode MYB binding sites, F-box family proteins, and glycosyl hydrolase family proteins. Previous studies have demonstrated these proteins’ involvement in grain development: F-box proteins in wheat and rice regulate plant growth and panicle size through the ubiquitin proteasome pathway (Jain et al., 2007), while MYB binding sites were shown to influence TGW in rye *LO7* lines (Dörthe et al., 2021; Bauer et al., 2017).

## Conclusion

This study conducted BSA-seq analysis on a hybrid rye population, identifying 10 candidate gene regions and 68 SNPs significantly associated with TGW traits. Analysis of these SNPs led to a successful development and validation of a functional KASP marker (*TGW-16*), which showed tight linkage with the candidate gene *ScWN7R01G304400*. Functional annotation revealed that *ScWN7R01G304400* encodes a protein containing an RRM1 (RNA recognition motif 1) domain, a protein family known to play important roles in RNA binding and post-transcriptional regulation. This discovery provides new molecular targets for genetic research on TGW traits in rye, suggesting that future studies should focus on this gene and its encoded protein family to elucidate their roles in grain weight determination. The developed *TGW-16* marker offers a practical tool for molecular breeding selection of high-yield rye varieties.

In conclusion, these findings provide valuable genetic resources and tools for screening and breeding rye varieties with improved yield traits.

## Supplemental Information

10.7717/peerj.20811/supp-1 Supplemental Information 1 BAS-seq results.

10.7717/peerj.20811/supp-2 Supplemental Information 2 The rye gene sequence.

10.7717/peerj.20811/supp-3 Supplemental Information 3 Relevant information of 68 SNP loci generated after BSA sequencing.

10.7717/peerj.20811/supp-4 Supplemental Information 4 Raw numerical data for plant measurements.

10.7717/peerj.20811/supp-5 Supplemental Information 5 Δ SNP-index.

10.7717/peerj.20811/supp-6 Supplemental Information 6 ΔIndel-index.

10.7717/peerj.20811/supp-7 Supplemental Information 7 ΔAll-index.

10.7717/peerj.20811/supp-8 Supplemental Information 8 Results of SSR25 in the C_1_ population.

10.7717/peerj.20811/supp-9 Supplemental Information 9 Genotyping plot of KASP markers in C _1_ population.

10.7717/peerj.20811/supp-10 Supplemental Information 10 Expression status of seven genes.

10.7717/peerj.20811/supp-11 Supplemental Information 11 Genotyping plot of *TGW-16* marker detection in C_2_ population.

10.7717/peerj.20811/supp-12 Supplemental Information 12 Comparison of TGW of plants screened by molecular markers.

## Additional Information and Declarations

### Data Availability

The following information was supplied regarding data availability:

The raw data is available in the Supplemental Files.
