# Supplementary figures and images for: Gene mapping and development of molecular markers for thousand-grain weight in rye based on bulked segregant analysis (part 2 of 2)
Source: PeerJ. 2026 Feb 12;14:e20811. doi: 10.7717/peerj.20811 (PMC12906707; doi:10.7717/peerj.20811)

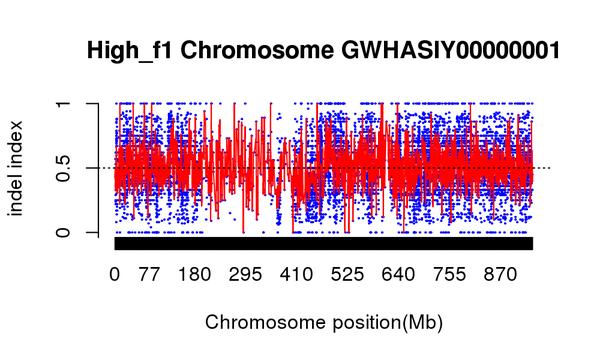

Supplement: Supplemental Information 1 [file peerj-14-20811-s001.zip › Supplementary 1/src/images/High_f1.InDel_index.GWHASIY00000001.png]

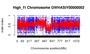

Supplement: Supplemental Information 1 [file peerj-14-20811-s001.zip › Supplementary 1/src/images/High_f1.InDel_index.GWHASIY00000002.JPEG]

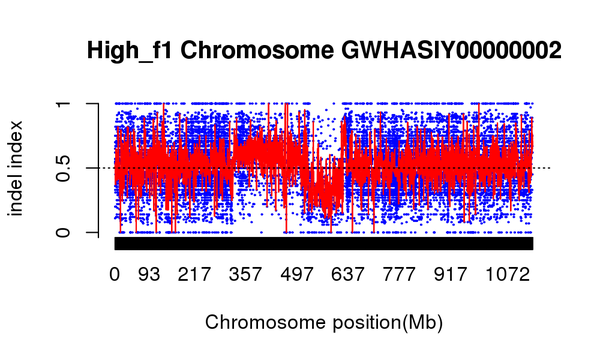

Supplement: Supplemental Information 1 [file peerj-14-20811-s001.zip › Supplementary 1/src/images/High_f1.InDel_index.GWHASIY00000002.png]

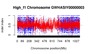

Supplement: Supplemental Information 1 [file peerj-14-20811-s001.zip › Supplementary 1/src/images/High_f1.InDel_index.GWHASIY00000003.JPEG]

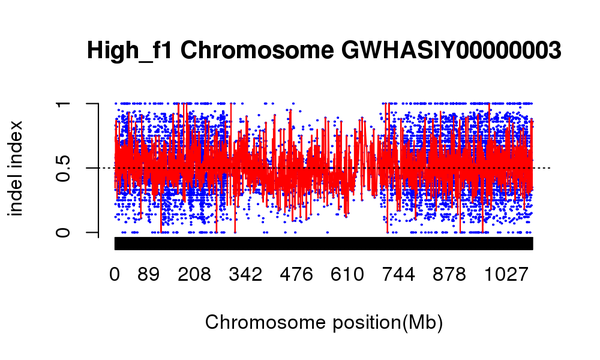

Supplement: Supplemental Information 1 [file peerj-14-20811-s001.zip › Supplementary 1/src/images/High_f1.InDel_index.GWHASIY00000003.png]

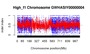

Supplement: Supplemental Information 1 [file peerj-14-20811-s001.zip › Supplementary 1/src/images/High_f1.InDel_index.GWHASIY00000004.JPEG]

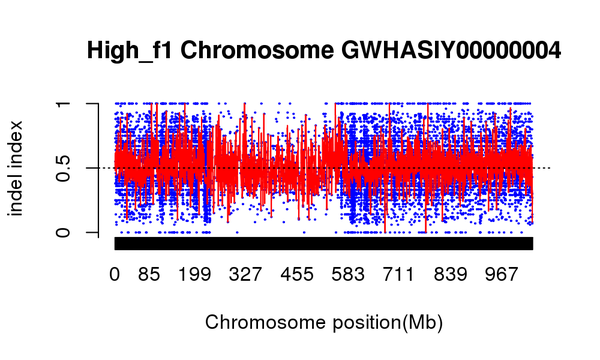

Supplement: Supplemental Information 1 [file peerj-14-20811-s001.zip › Supplementary 1/src/images/High_f1.InDel_index.GWHASIY00000004.png]

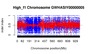

Supplement: Supplemental Information 1 [file peerj-14-20811-s001.zip › Supplementary 1/src/images/High_f1.InDel_index.GWHASIY00000005.JPEG]

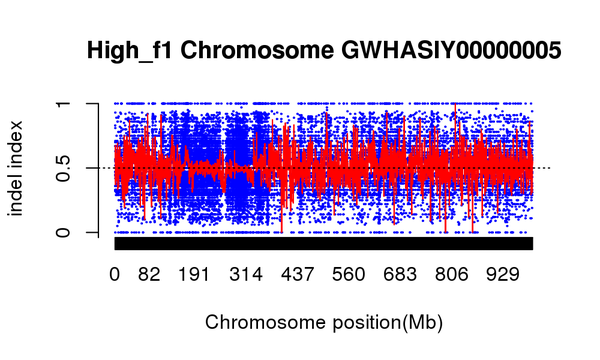

Supplement: Supplemental Information 1 [file peerj-14-20811-s001.zip › Supplementary 1/src/images/High_f1.InDel_index.GWHASIY00000005.png]

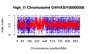

Supplement: Supplemental Information 1 [file peerj-14-20811-s001.zip › Supplementary 1/src/images/High_f1.InDel_index.GWHASIY00000006.JPEG]

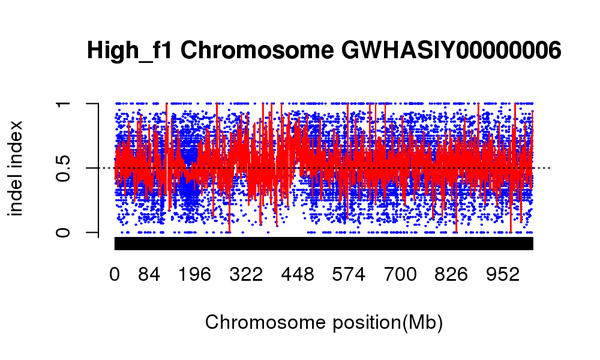

Supplement: Supplemental Information 1 [file peerj-14-20811-s001.zip › Supplementary 1/src/images/High_f1.InDel_index.GWHASIY00000006.png]

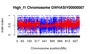

Supplement: Supplemental Information 1 [file peerj-14-20811-s001.zip › Supplementary 1/src/images/High_f1.InDel_index.GWHASIY00000007.JPEG]

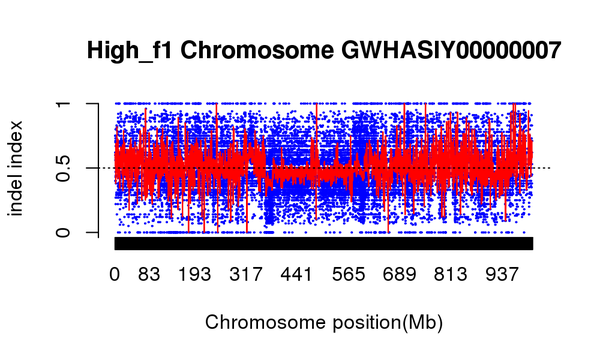

Supplement: Supplemental Information 1 [file peerj-14-20811-s001.zip › Supplementary 1/src/images/High_f1.InDel_index.GWHASIY00000007.png]

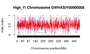

Supplement: Supplemental Information 1 [file peerj-14-20811-s001.zip › Supplementary 1/src/images/High_f1.InDel_index.GWHASIY00000008.JPEG]

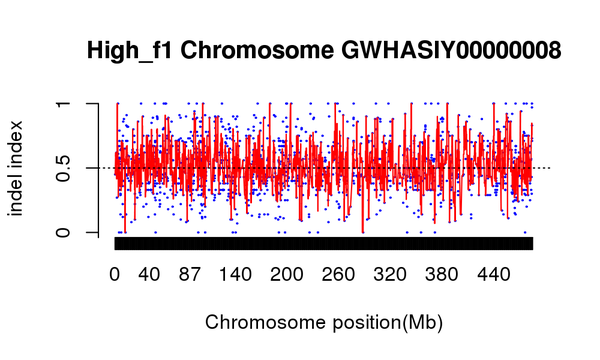

Supplement: Supplemental Information 1 [file peerj-14-20811-s001.zip › Supplementary 1/src/images/High_f1.InDel_index.GWHASIY00000008.png]

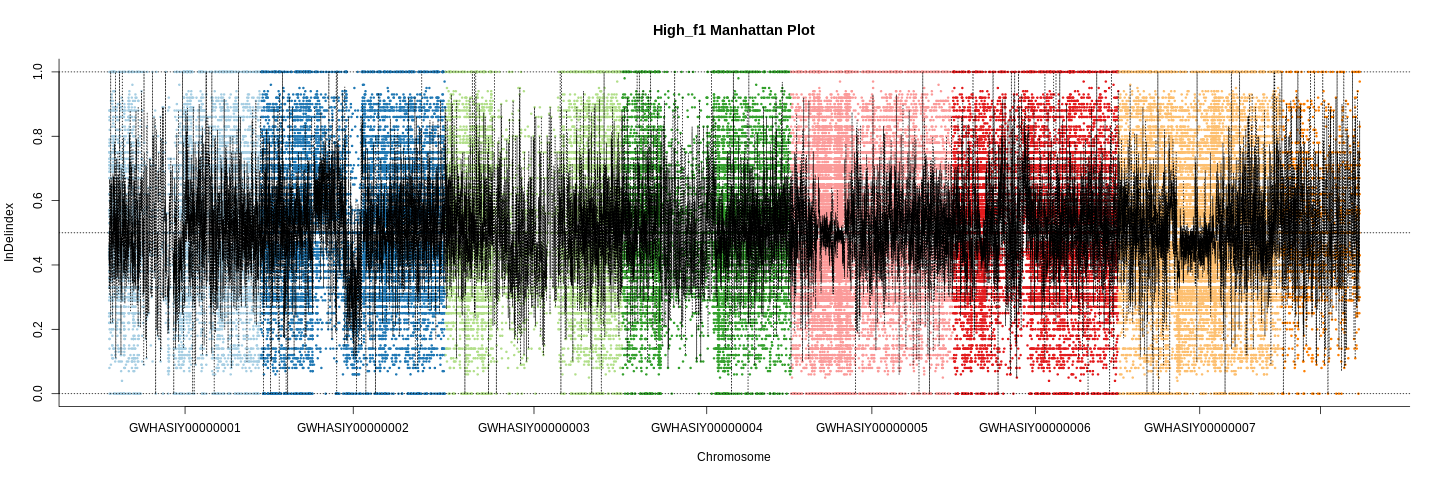

Supplement: Supplemental Information 1 [file peerj-14-20811-s001.zip › Supplementary 1/src/images/High_f1.InDel_index.manhattan.png]

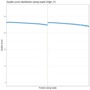

Supplement: Supplemental Information 1 [file peerj-14-20811-s001.zip › Supplementary 1/src/images/High_f1.quality_distribution.JPEG]

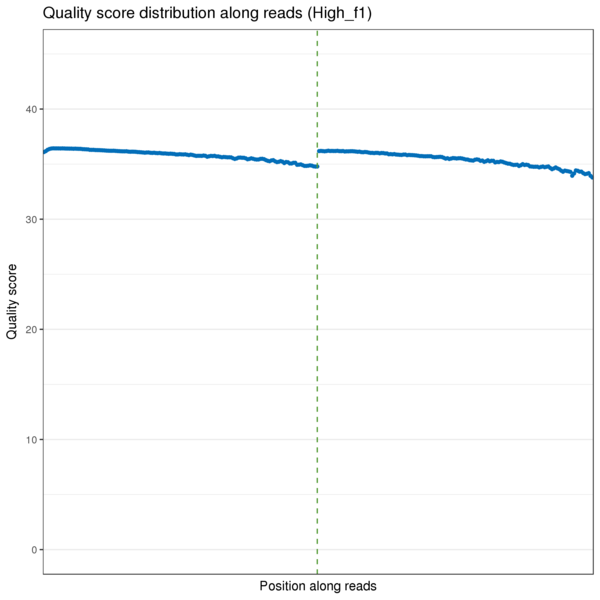

Supplement: Supplemental Information 1 [file peerj-14-20811-s001.zip › Supplementary 1/src/images/High_f1.quality_distribution.png]

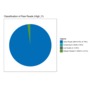

Supplement: Supplemental Information 1 [file peerj-14-20811-s001.zip › Supplementary 1/src/images/High_f1.raw_reads_classification.JPEG]

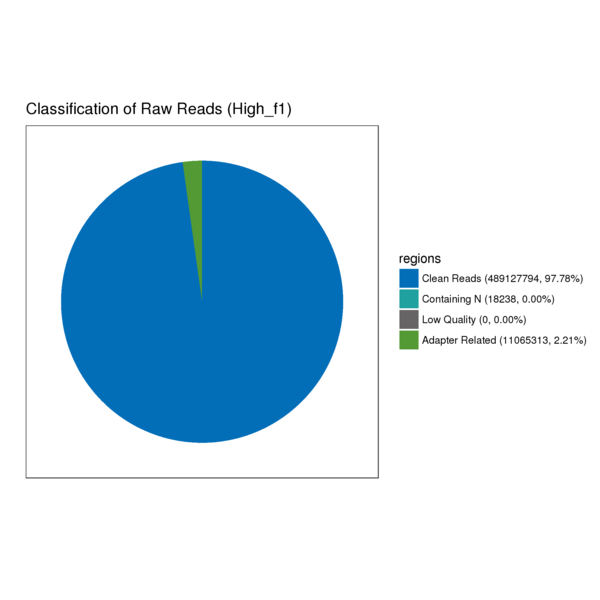

Supplement: Supplemental Information 1 [file peerj-14-20811-s001.zip › Supplementary 1/src/images/High_f1.raw_reads_classification.png]

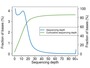

Supplement: Supplemental Information 1 [file peerj-14-20811-s001.zip › Supplementary 1/src/images/High_f1.Sequencing-depth.JPEG]

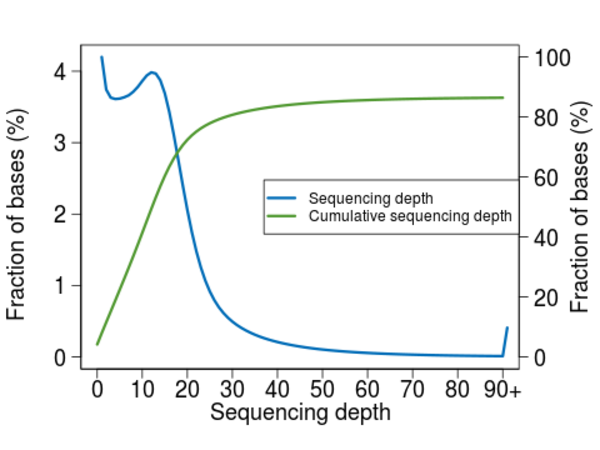

Supplement: Supplemental Information 1 [file peerj-14-20811-s001.zip › Supplementary 1/src/images/High_f1.Sequencing-depth.png]

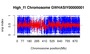

Supplement: Supplemental Information 1 [file peerj-14-20811-s001.zip › Supplementary 1/src/images/High_f1.SNP_index.GWHASIY00000001.JPEG]

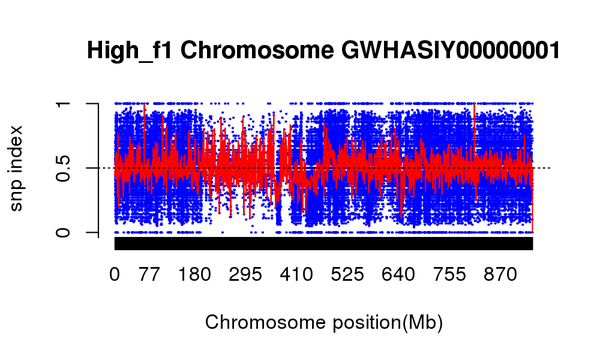

Supplement: Supplemental Information 1 [file peerj-14-20811-s001.zip › Supplementary 1/src/images/High_f1.SNP_index.GWHASIY00000001.png]

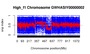

Supplement: Supplemental Information 1 [file peerj-14-20811-s001.zip › Supplementary 1/src/images/High_f1.SNP_index.GWHASIY00000002.JPEG]

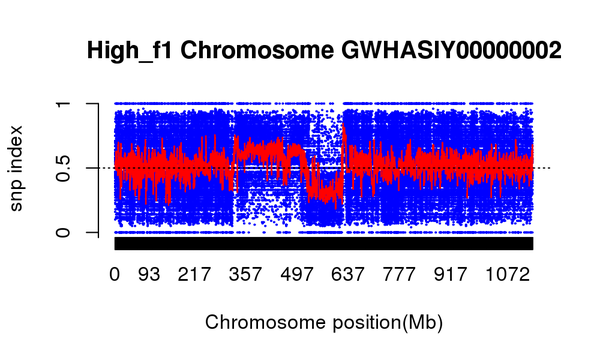

Supplement: Supplemental Information 1 [file peerj-14-20811-s001.zip › Supplementary 1/src/images/High_f1.SNP_index.GWHASIY00000002.png]

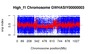

Supplement: Supplemental Information 1 [file peerj-14-20811-s001.zip › Supplementary 1/src/images/High_f1.SNP_index.GWHASIY00000003.JPEG]

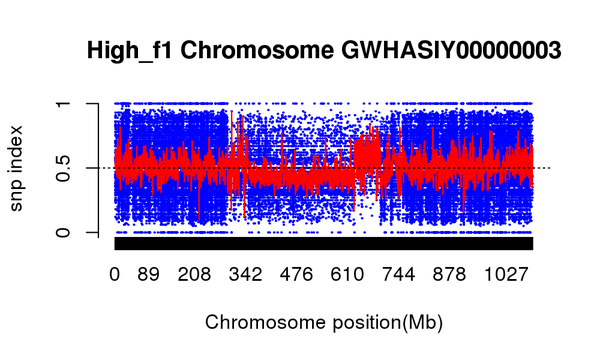

Supplement: Supplemental Information 1 [file peerj-14-20811-s001.zip › Supplementary 1/src/images/High_f1.SNP_index.GWHASIY00000003.png]

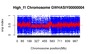

Supplement: Supplemental Information 1 [file peerj-14-20811-s001.zip › Supplementary 1/src/images/High_f1.SNP_index.GWHASIY00000004.JPEG]

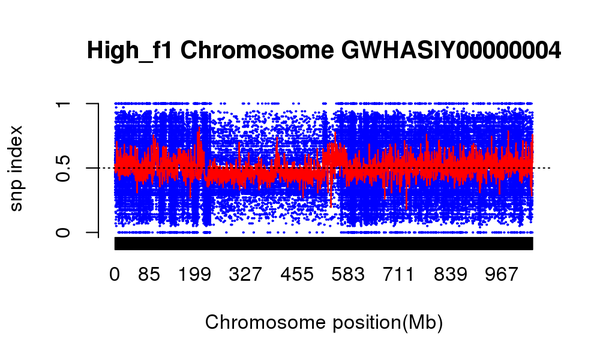

Supplement: Supplemental Information 1 [file peerj-14-20811-s001.zip › Supplementary 1/src/images/High_f1.SNP_index.GWHASIY00000004.png]

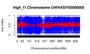

Supplement: Supplemental Information 1 [file peerj-14-20811-s001.zip › Supplementary 1/src/images/High_f1.SNP_index.GWHASIY00000005.JPEG]

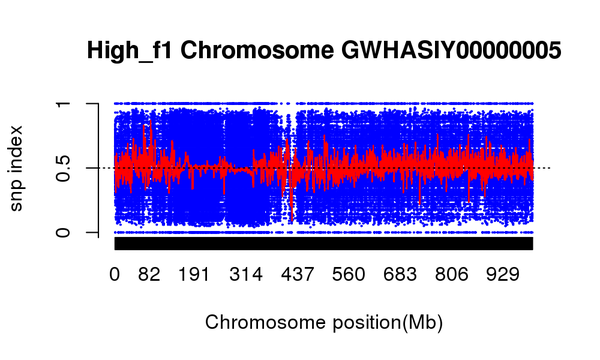

Supplement: Supplemental Information 1 [file peerj-14-20811-s001.zip › Supplementary 1/src/images/High_f1.SNP_index.GWHASIY00000005.png]

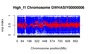

Supplement: Supplemental Information 1 [file peerj-14-20811-s001.zip › Supplementary 1/src/images/High_f1.SNP_index.GWHASIY00000006.JPEG]

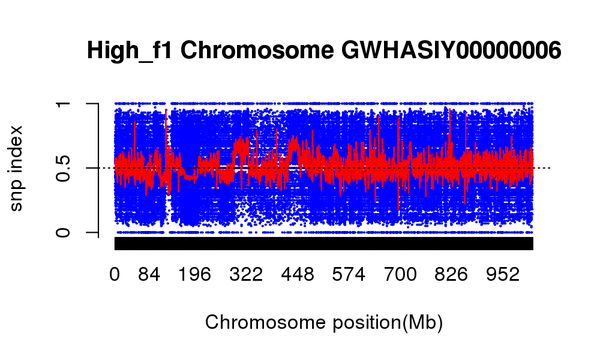

Supplement: Supplemental Information 1 [file peerj-14-20811-s001.zip › Supplementary 1/src/images/High_f1.SNP_index.GWHASIY00000006.png]

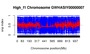

Supplement: Supplemental Information 1 [file peerj-14-20811-s001.zip › Supplementary 1/src/images/High_f1.SNP_index.GWHASIY00000007.JPEG]

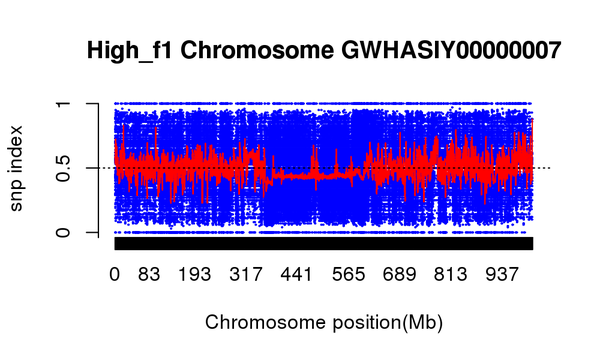

Supplement: Supplemental Information 1 [file peerj-14-20811-s001.zip › Supplementary 1/src/images/High_f1.SNP_index.GWHASIY00000007.png]

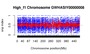

Supplement: Supplemental Information 1 [file peerj-14-20811-s001.zip › Supplementary 1/src/images/High_f1.SNP_index.GWHASIY00000008.JPEG]

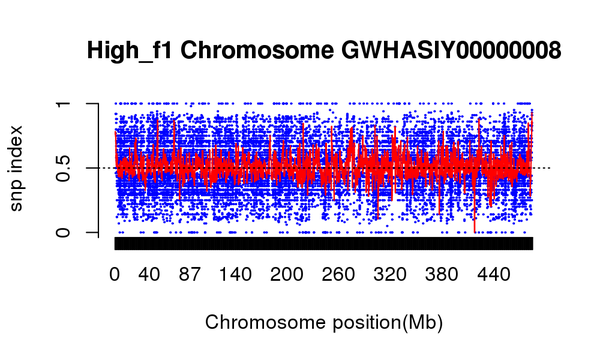

Supplement: Supplemental Information 1 [file peerj-14-20811-s001.zip › Supplementary 1/src/images/High_f1.SNP_index.GWHASIY00000008.png]

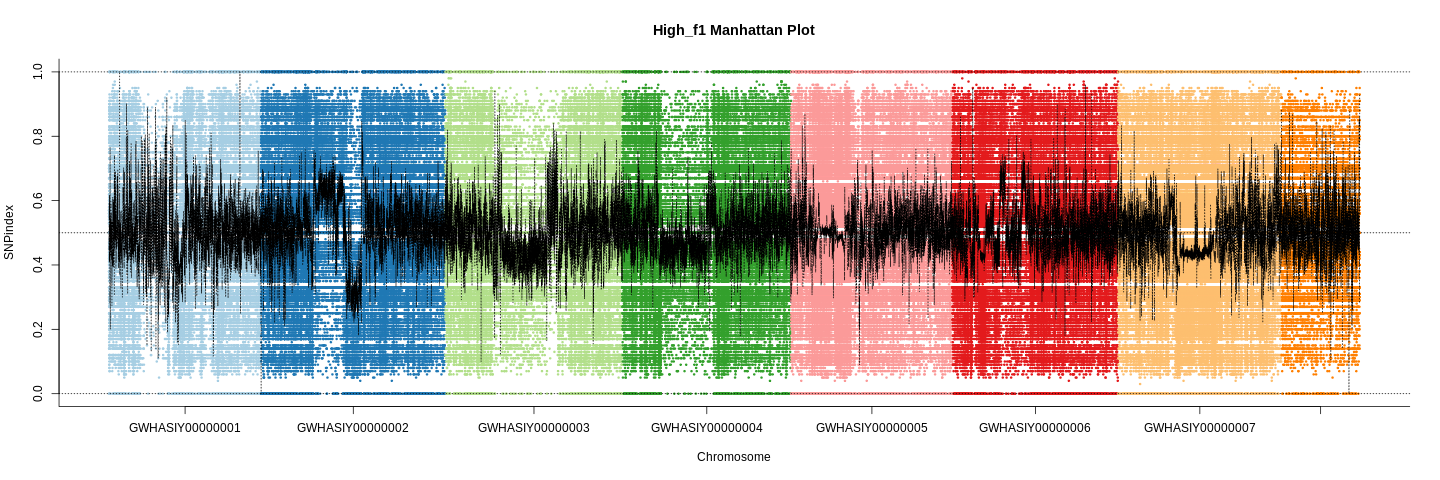

Supplement: Supplemental Information 1 [file peerj-14-20811-s001.zip › Supplementary 1/src/images/High_f1.SNP_index.manhattan.png]

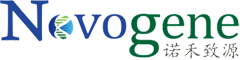

Supplement: Supplemental Information 1 [file peerj-14-20811-s001.zip › Supplementary 1/src/images/logo.png]

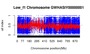

Supplement: Supplemental Information 1 [file peerj-14-20811-s001.zip › Supplementary 1/src/images/Low_f1.All_index.GWHASIY00000001.JPEG]

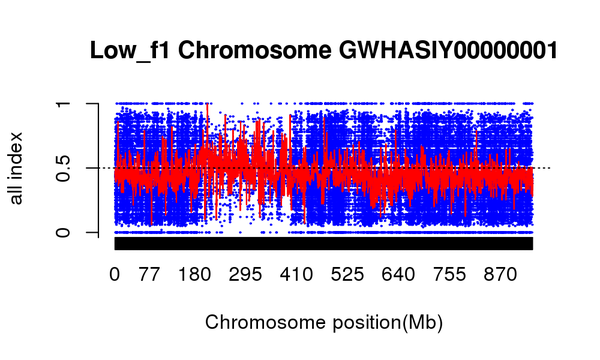

Supplement: Supplemental Information 1 [file peerj-14-20811-s001.zip › Supplementary 1/src/images/Low_f1.All_index.GWHASIY00000001.png]

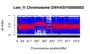

Supplement: Supplemental Information 1 [file peerj-14-20811-s001.zip › Supplementary 1/src/images/Low_f1.All_index.GWHASIY00000002.JPEG]

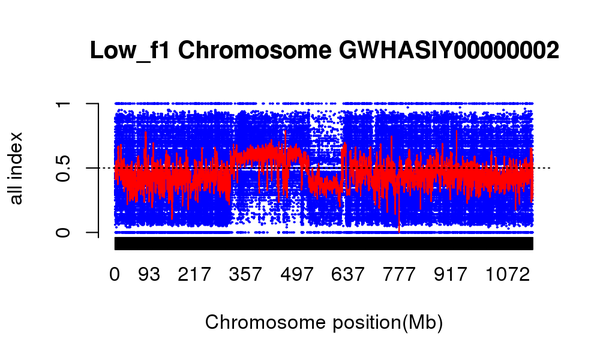

Supplement: Supplemental Information 1 [file peerj-14-20811-s001.zip › Supplementary 1/src/images/Low_f1.All_index.GWHASIY00000002.png]

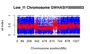

Supplement: Supplemental Information 1 [file peerj-14-20811-s001.zip › Supplementary 1/src/images/Low_f1.All_index.GWHASIY00000003.JPEG]

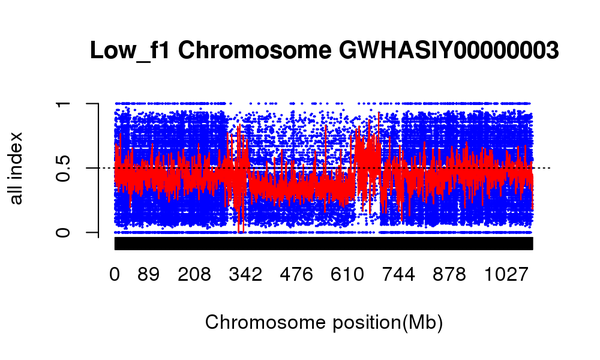

Supplement: Supplemental Information 1 [file peerj-14-20811-s001.zip › Supplementary 1/src/images/Low_f1.All_index.GWHASIY00000003.png]

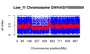

Supplement: Supplemental Information 1 [file peerj-14-20811-s001.zip › Supplementary 1/src/images/Low_f1.All_index.GWHASIY00000004.JPEG]

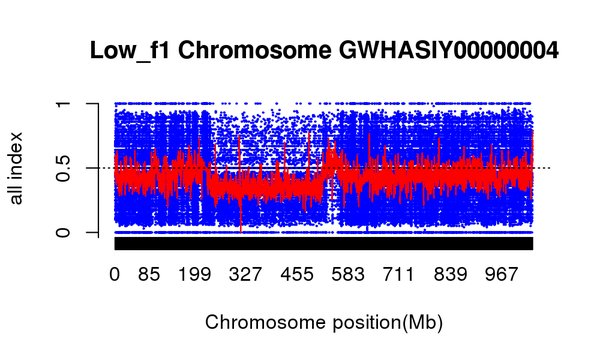

Supplement: Supplemental Information 1 [file peerj-14-20811-s001.zip › Supplementary 1/src/images/Low_f1.All_index.GWHASIY00000004.png]

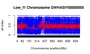

Supplement: Supplemental Information 1 [file peerj-14-20811-s001.zip › Supplementary 1/src/images/Low_f1.All_index.GWHASIY00000005.JPEG]

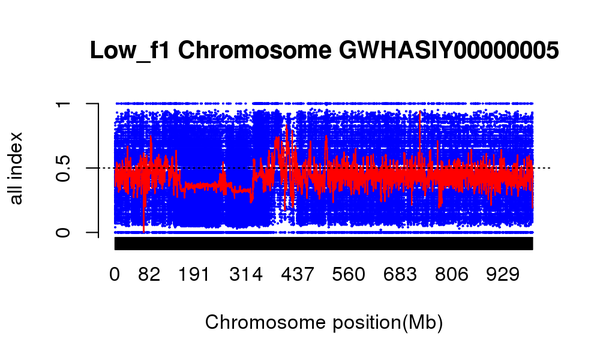

Supplement: Supplemental Information 1 [file peerj-14-20811-s001.zip › Supplementary 1/src/images/Low_f1.All_index.GWHASIY00000005.png]

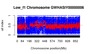

Supplement: Supplemental Information 1 [file peerj-14-20811-s001.zip › Supplementary 1/src/images/Low_f1.All_index.GWHASIY00000006.JPEG]

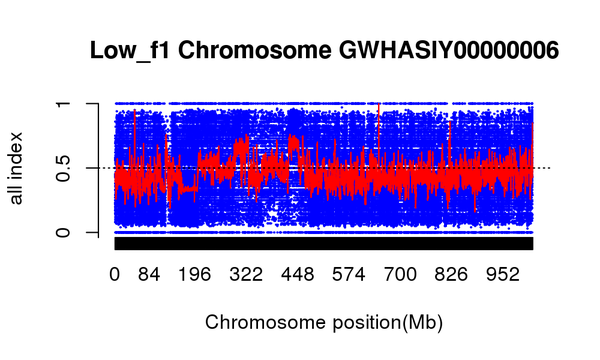

Supplement: Supplemental Information 1 [file peerj-14-20811-s001.zip › Supplementary 1/src/images/Low_f1.All_index.GWHASIY00000006.png]

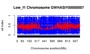

Supplement: Supplemental Information 1 [file peerj-14-20811-s001.zip › Supplementary 1/src/images/Low_f1.All_index.GWHASIY00000007.JPEG]

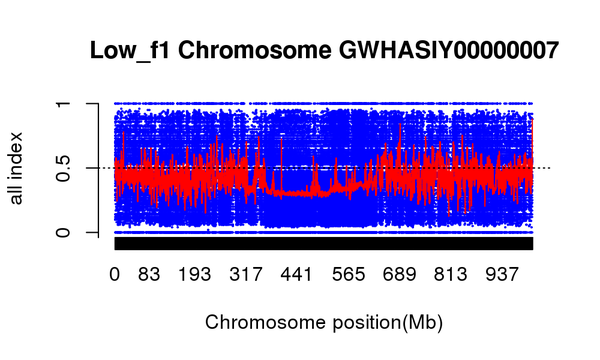

Supplement: Supplemental Information 1 [file peerj-14-20811-s001.zip › Supplementary 1/src/images/Low_f1.All_index.GWHASIY00000007.png]

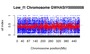

Supplement: Supplemental Information 1 [file peerj-14-20811-s001.zip › Supplementary 1/src/images/Low_f1.All_index.GWHASIY00000008.JPEG]

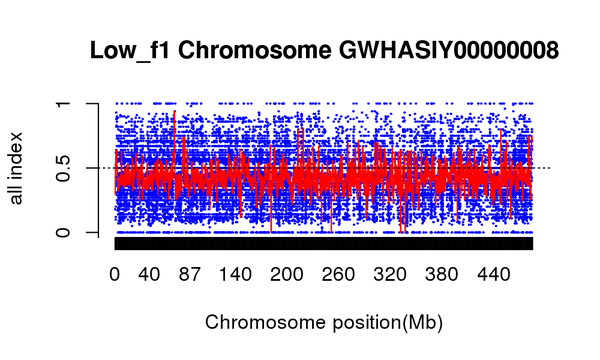

Supplement: Supplemental Information 1 [file peerj-14-20811-s001.zip › Supplementary 1/src/images/Low_f1.All_index.GWHASIY00000008.png]

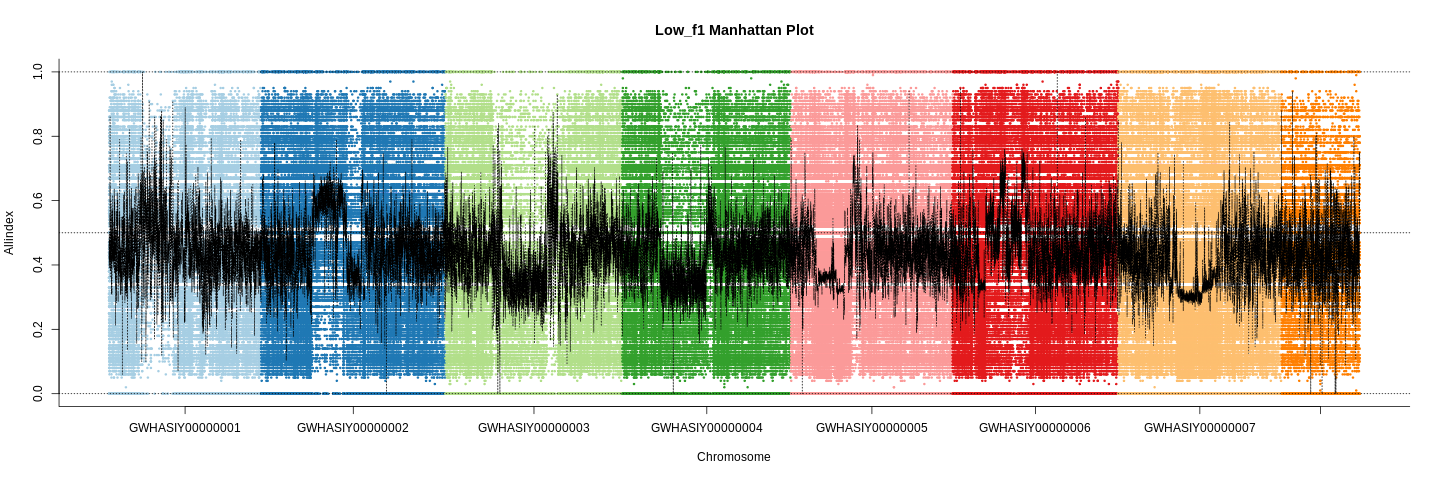

Supplement: Supplemental Information 1 [file peerj-14-20811-s001.zip › Supplementary 1/src/images/Low_f1.All_index.manhattan.png]

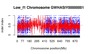

Supplement: Supplemental Information 1 [file peerj-14-20811-s001.zip › Supplementary 1/src/images/Low_f1.InDel_index.GWHASIY00000001.JPEG]

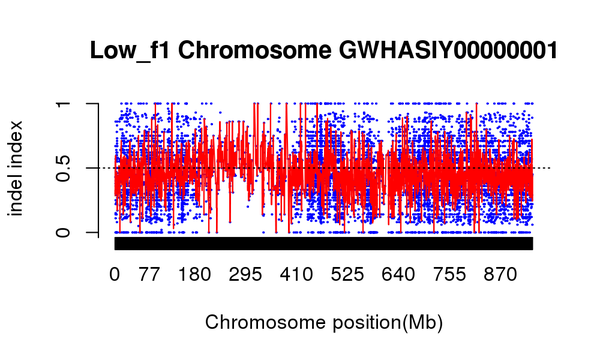

Supplement: Supplemental Information 1 [file peerj-14-20811-s001.zip › Supplementary 1/src/images/Low_f1.InDel_index.GWHASIY00000001.png]

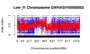

Supplement: Supplemental Information 1 [file peerj-14-20811-s001.zip › Supplementary 1/src/images/Low_f1.InDel_index.GWHASIY00000002.JPEG]

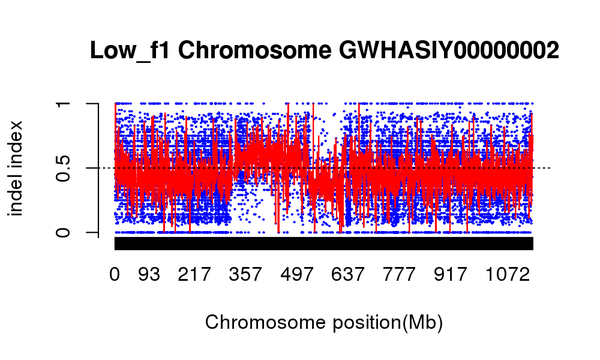

Supplement: Supplemental Information 1 [file peerj-14-20811-s001.zip › Supplementary 1/src/images/Low_f1.InDel_index.GWHASIY00000002.png]

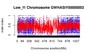

Supplement: Supplemental Information 1 [file peerj-14-20811-s001.zip › Supplementary 1/src/images/Low_f1.InDel_index.GWHASIY00000003.JPEG]

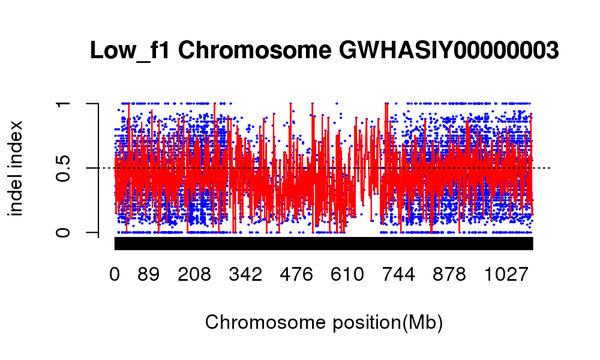

Supplement: Supplemental Information 1 [file peerj-14-20811-s001.zip › Supplementary 1/src/images/Low_f1.InDel_index.GWHASIY00000003.png]

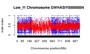

Supplement: Supplemental Information 1 [file peerj-14-20811-s001.zip › Supplementary 1/src/images/Low_f1.InDel_index.GWHASIY00000004.JPEG]

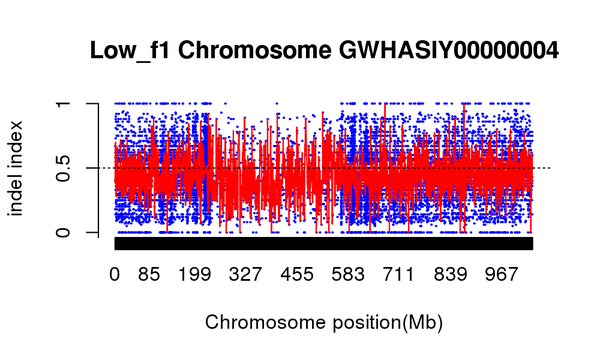

Supplement: Supplemental Information 1 [file peerj-14-20811-s001.zip › Supplementary 1/src/images/Low_f1.InDel_index.GWHASIY00000004.png]

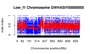

Supplement: Supplemental Information 1 [file peerj-14-20811-s001.zip › Supplementary 1/src/images/Low_f1.InDel_index.GWHASIY00000005.JPEG]

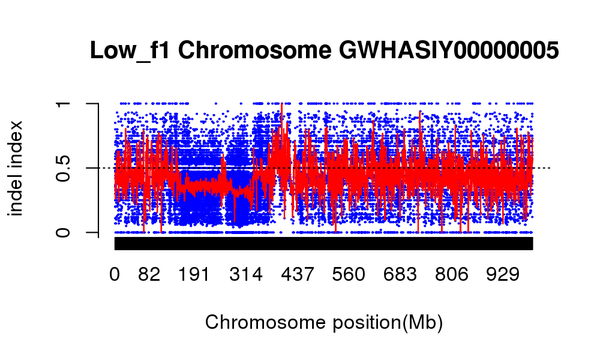

Supplement: Supplemental Information 1 [file peerj-14-20811-s001.zip › Supplementary 1/src/images/Low_f1.InDel_index.GWHASIY00000005.png]

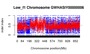

Supplement: Supplemental Information 1 [file peerj-14-20811-s001.zip › Supplementary 1/src/images/Low_f1.InDel_index.GWHASIY00000006.JPEG]

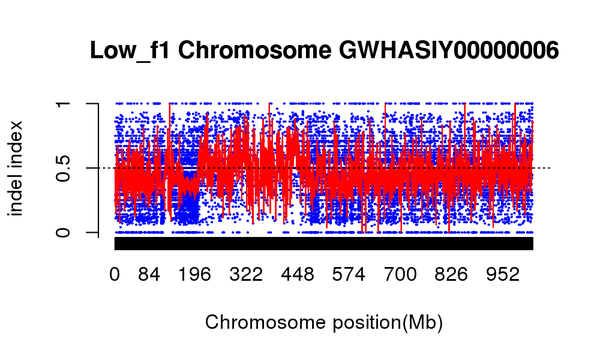

Supplement: Supplemental Information 1 [file peerj-14-20811-s001.zip › Supplementary 1/src/images/Low_f1.InDel_index.GWHASIY00000006.png]

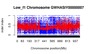

Supplement: Supplemental Information 1 [file peerj-14-20811-s001.zip › Supplementary 1/src/images/Low_f1.InDel_index.GWHASIY00000007.JPEG]

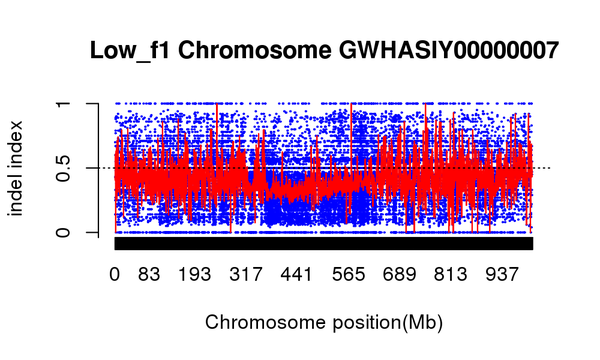

Supplement: Supplemental Information 1 [file peerj-14-20811-s001.zip › Supplementary 1/src/images/Low_f1.InDel_index.GWHASIY00000007.png]

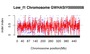

Supplement: Supplemental Information 1 [file peerj-14-20811-s001.zip › Supplementary 1/src/images/Low_f1.InDel_index.GWHASIY00000008.JPEG]

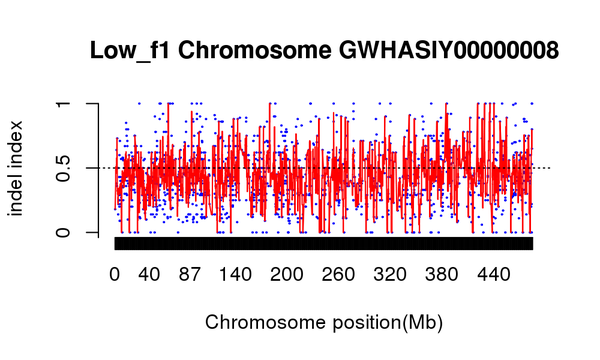

Supplement: Supplemental Information 1 [file peerj-14-20811-s001.zip › Supplementary 1/src/images/Low_f1.InDel_index.GWHASIY00000008.png]

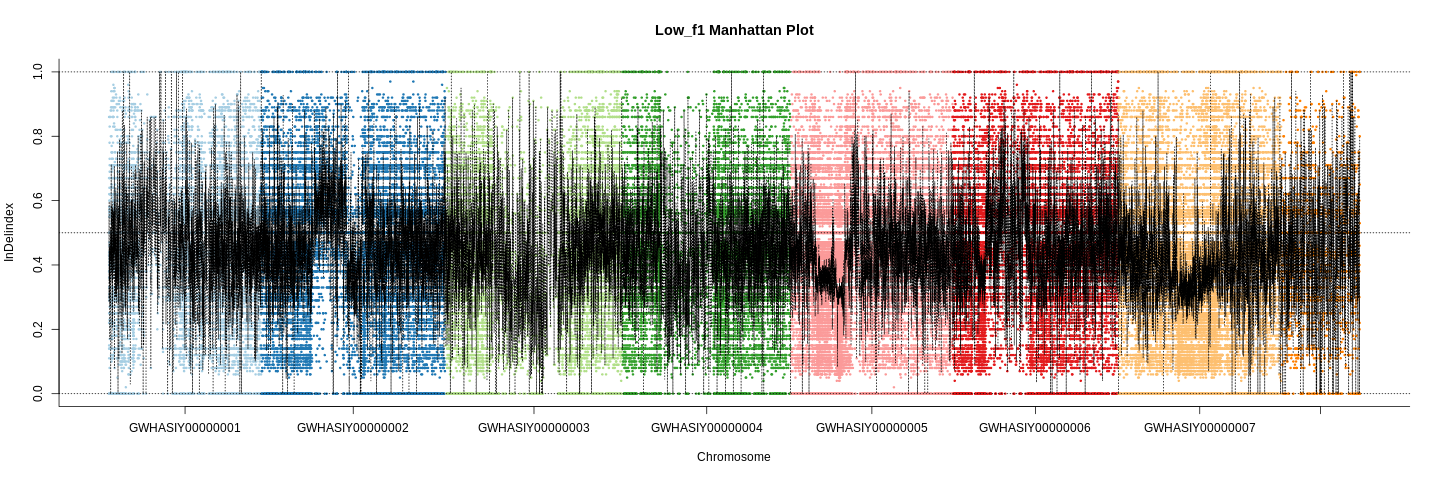

Supplement: Supplemental Information 1 [file peerj-14-20811-s001.zip › Supplementary 1/src/images/Low_f1.InDel_index.manhattan.png]

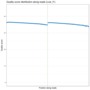

Supplement: Supplemental Information 1 [file peerj-14-20811-s001.zip › Supplementary 1/src/images/Low_f1.quality_distribution.JPEG]

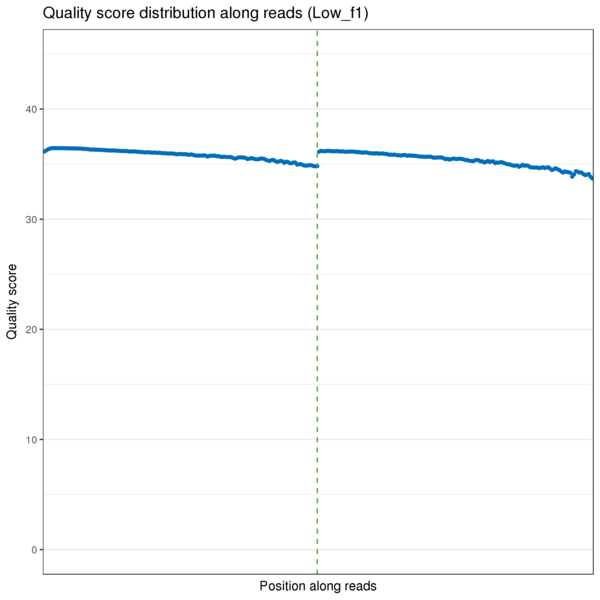

Supplement: Supplemental Information 1 [file peerj-14-20811-s001.zip › Supplementary 1/src/images/Low_f1.quality_distribution.png]

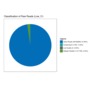

Supplement: Supplemental Information 1 [file peerj-14-20811-s001.zip › Supplementary 1/src/images/Low_f1.raw_reads_classification.JPEG]

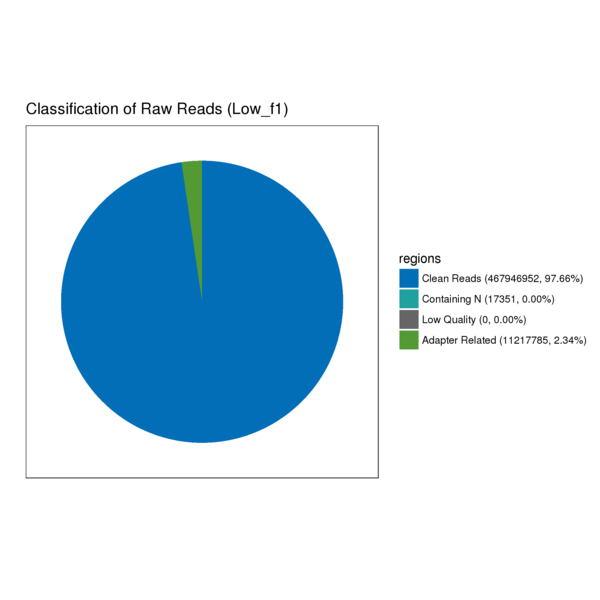

Supplement: Supplemental Information 1 [file peerj-14-20811-s001.zip › Supplementary 1/src/images/Low_f1.raw_reads_classification.png]

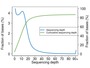

Supplement: Supplemental Information 1 [file peerj-14-20811-s001.zip › Supplementary 1/src/images/Low_f1.Sequencing-depth.JPEG]

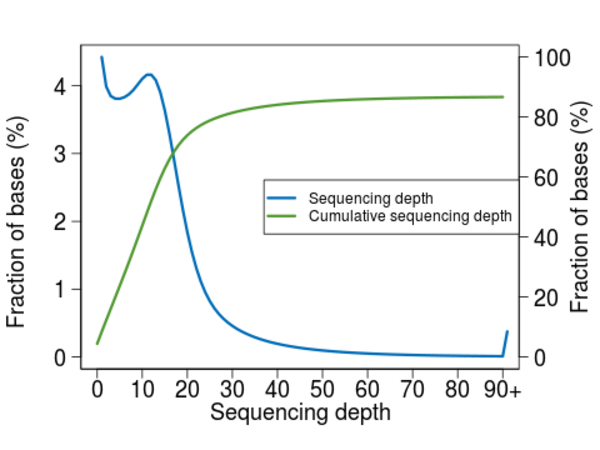

Supplement: Supplemental Information 1 [file peerj-14-20811-s001.zip › Supplementary 1/src/images/Low_f1.Sequencing-depth.png]

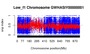

Supplement: Supplemental Information 1 [file peerj-14-20811-s001.zip › Supplementary 1/src/images/Low_f1.SNP_index.GWHASIY00000001.JPEG]

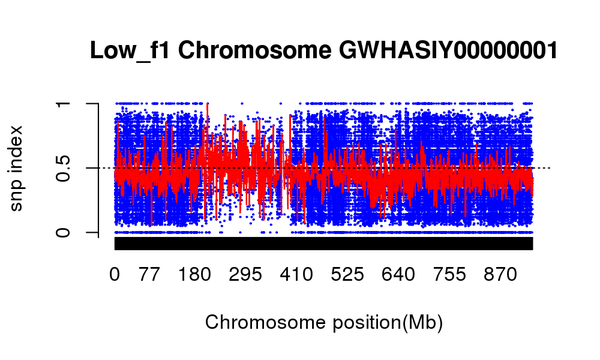

Supplement: Supplemental Information 1 [file peerj-14-20811-s001.zip › Supplementary 1/src/images/Low_f1.SNP_index.GWHASIY00000001.png]

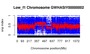

Supplement: Supplemental Information 1 [file peerj-14-20811-s001.zip › Supplementary 1/src/images/Low_f1.SNP_index.GWHASIY00000002.JPEG]

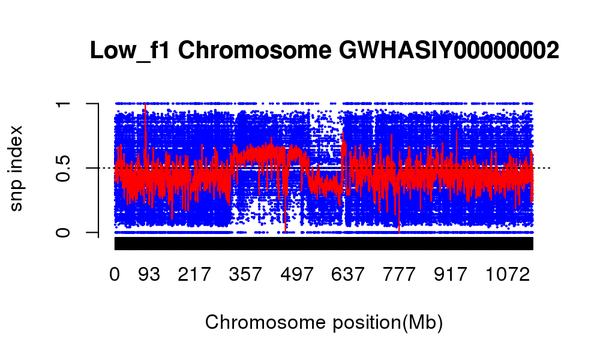

Supplement: Supplemental Information 1 [file peerj-14-20811-s001.zip › Supplementary 1/src/images/Low_f1.SNP_index.GWHASIY00000002.png]

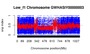

Supplement: Supplemental Information 1 [file peerj-14-20811-s001.zip › Supplementary 1/src/images/Low_f1.SNP_index.GWHASIY00000003.JPEG]

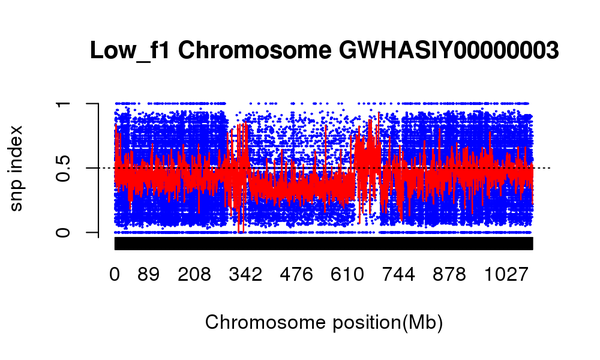

Supplement: Supplemental Information 1 [file peerj-14-20811-s001.zip › Supplementary 1/src/images/Low_f1.SNP_index.GWHASIY00000003.png]

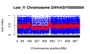

Supplement: Supplemental Information 1 [file peerj-14-20811-s001.zip › Supplementary 1/src/images/Low_f1.SNP_index.GWHASIY00000004.JPEG]

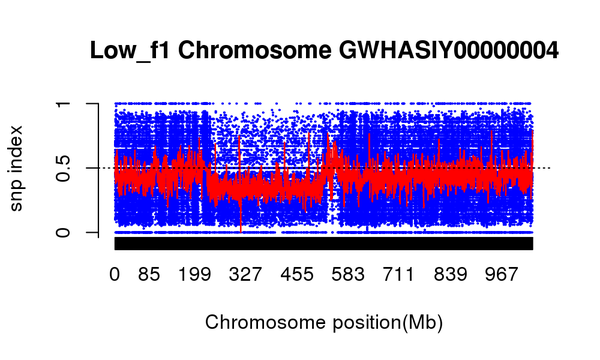

Supplement: Supplemental Information 1 [file peerj-14-20811-s001.zip › Supplementary 1/src/images/Low_f1.SNP_index.GWHASIY00000004.png]

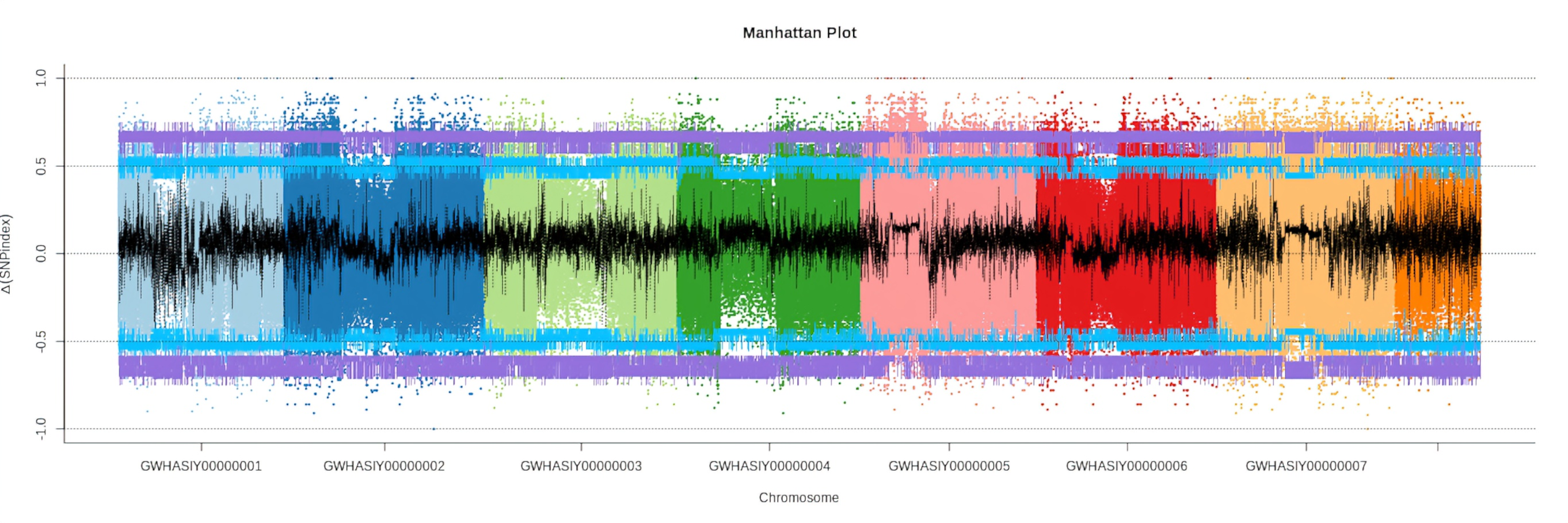

Supplement: Supplemental Information 5 [file peerj-14-20811-s005.png]

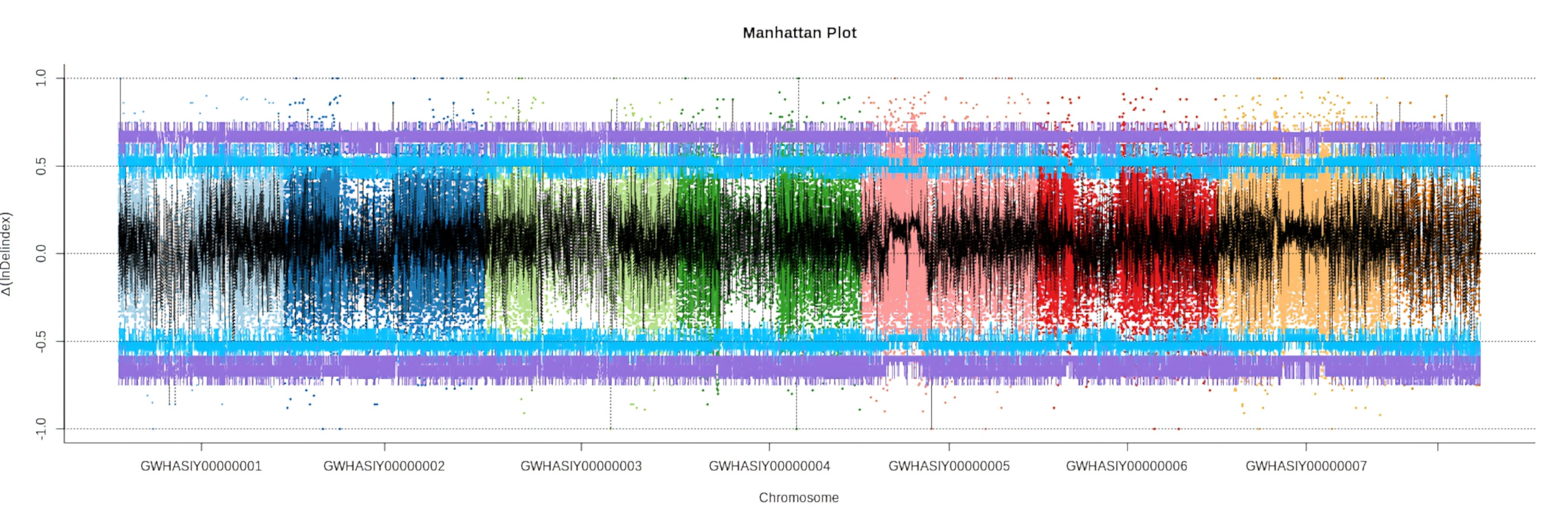

Supplement: Supplemental Information 6 [file peerj-14-20811-s006.png]

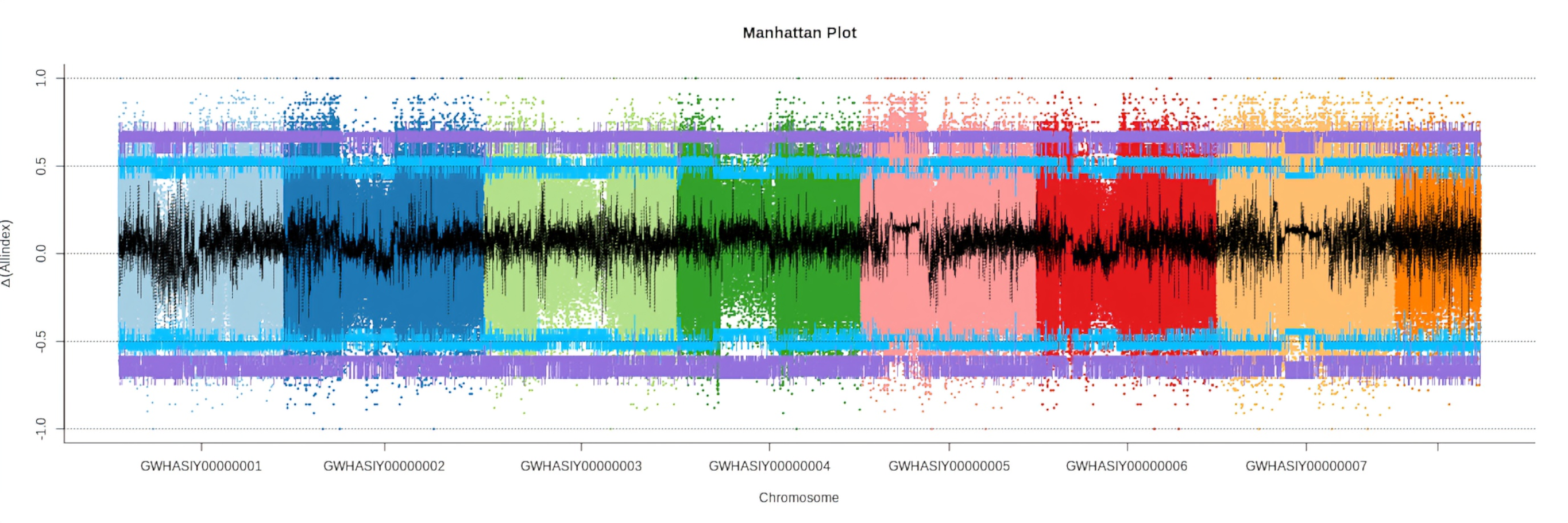

Supplement: Supplemental Information 7 [file peerj-14-20811-s007.png]

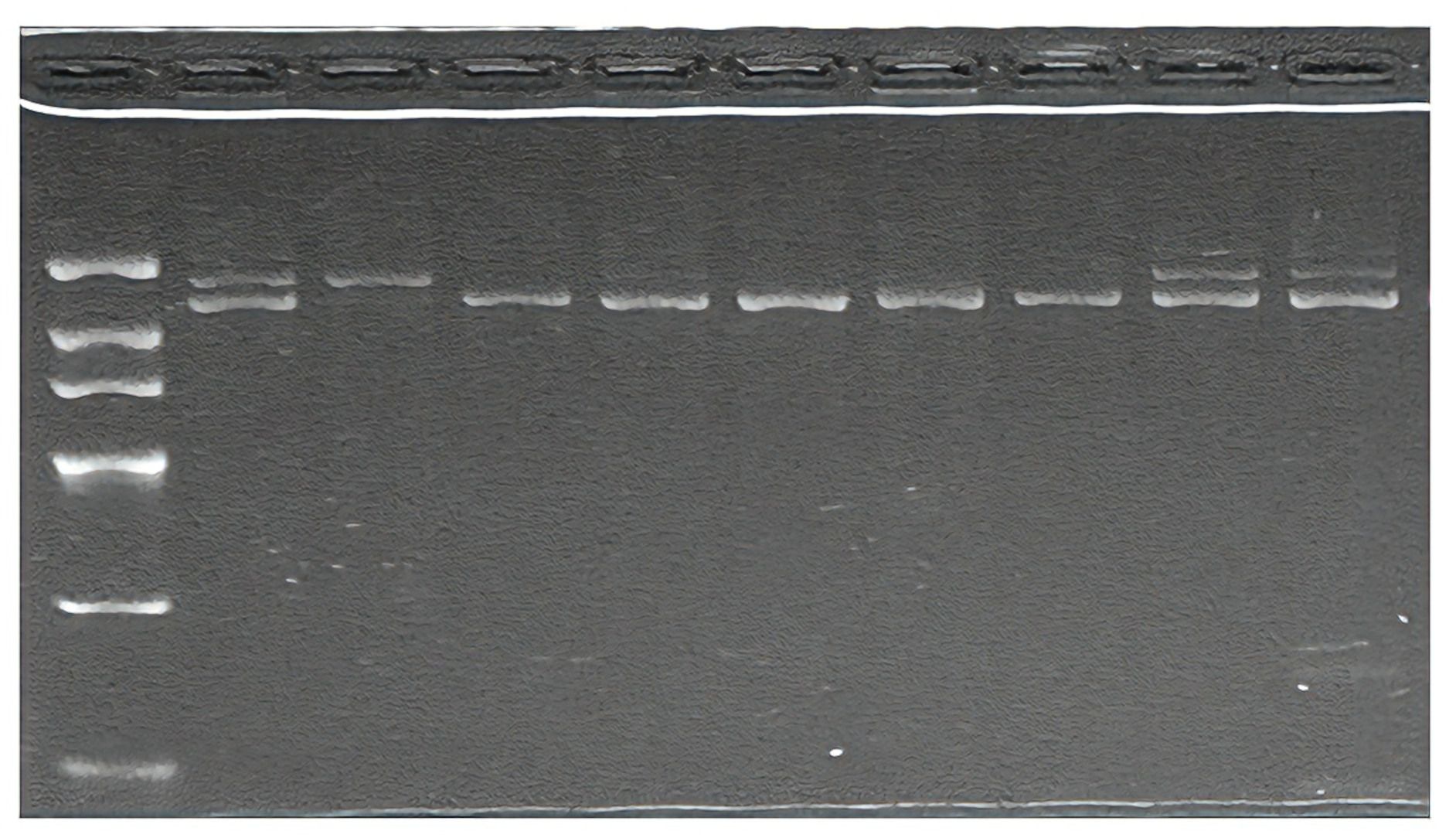

Supplement: Supplemental Information 8 [file peerj-14-20811-s008.png]

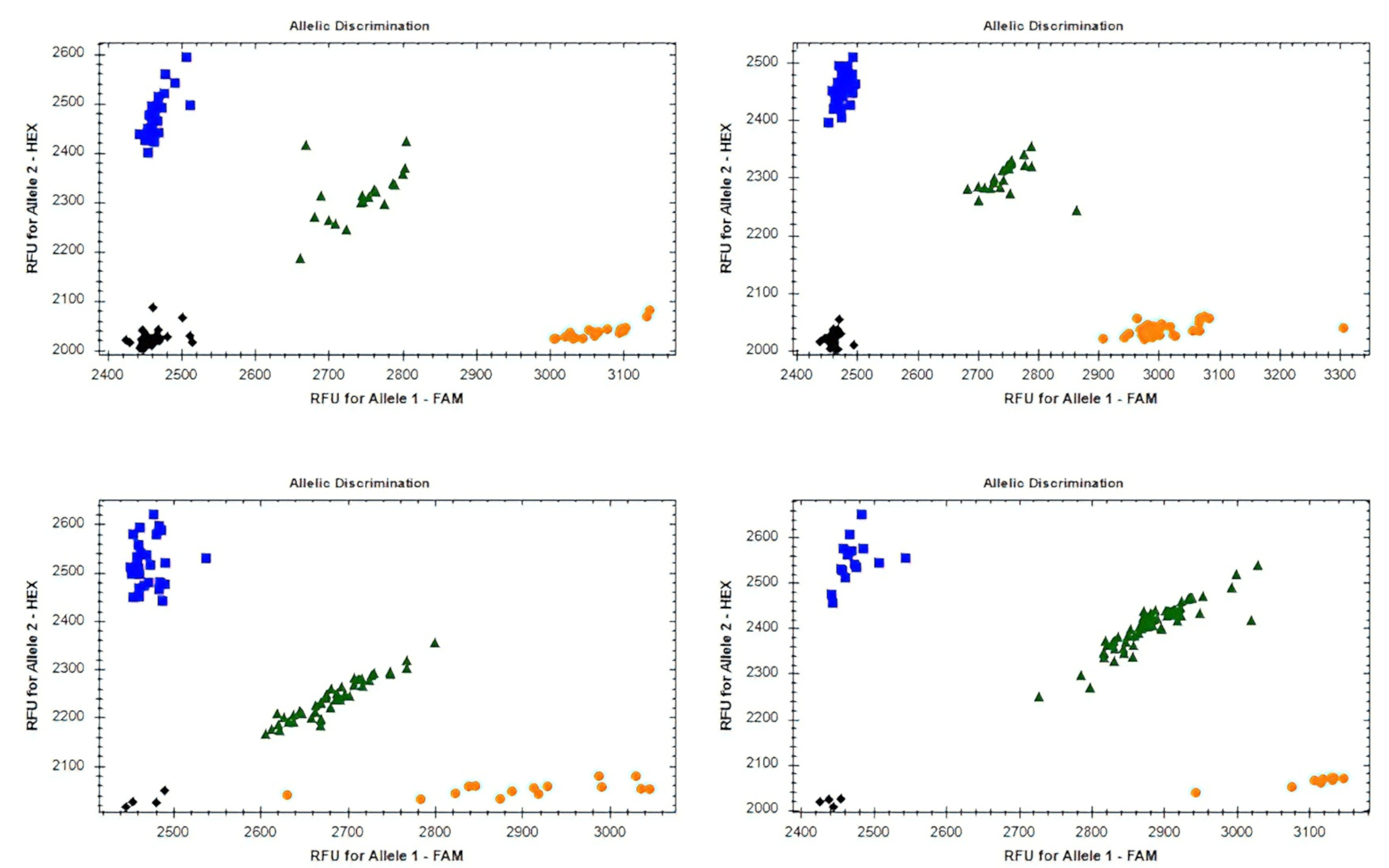

Supplement: Supplemental Information 9 [file peerj-14-20811-s009.png]

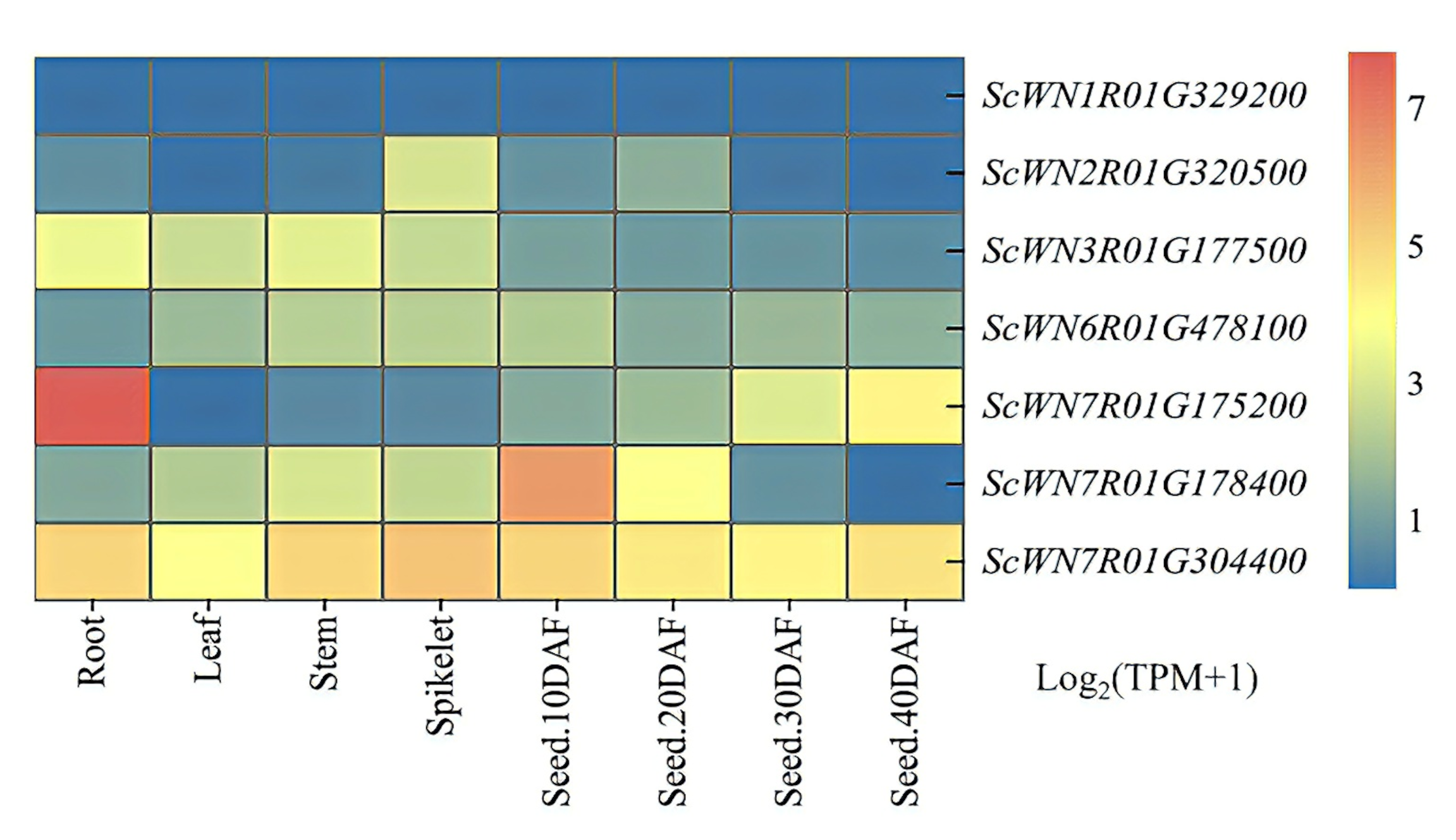

Supplement: Supplemental Information 10 [file peerj-14-20811-s010.png]

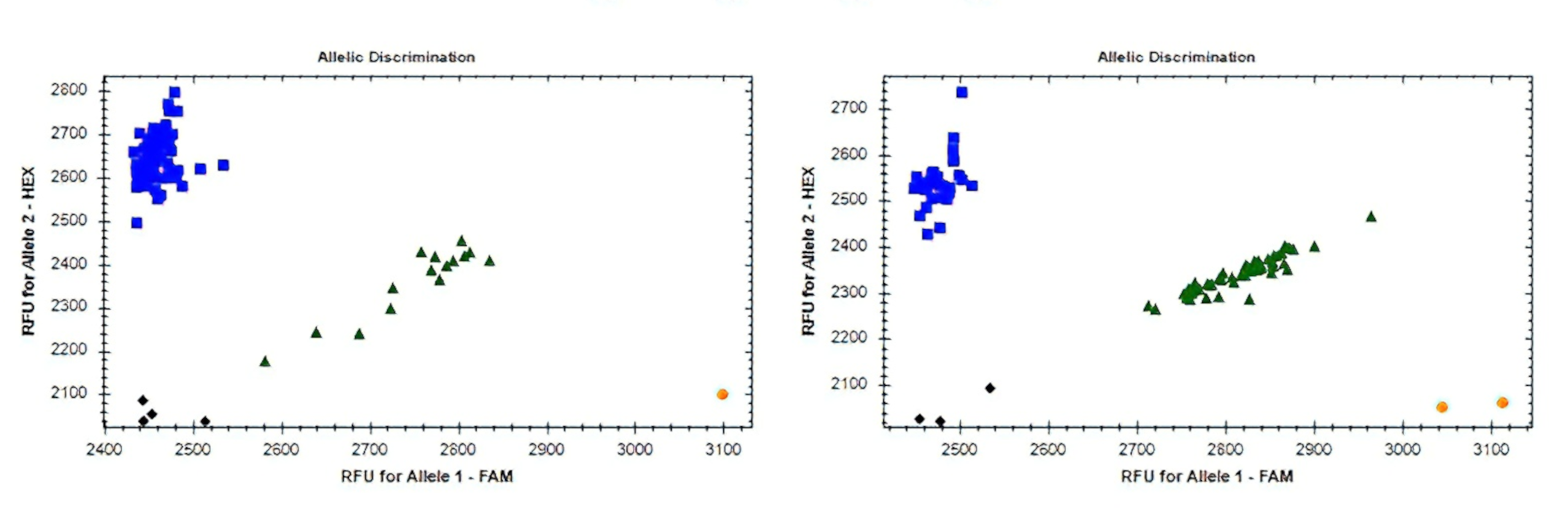

Supplement: Supplemental Information 11 [file peerj-14-20811-s011.png]

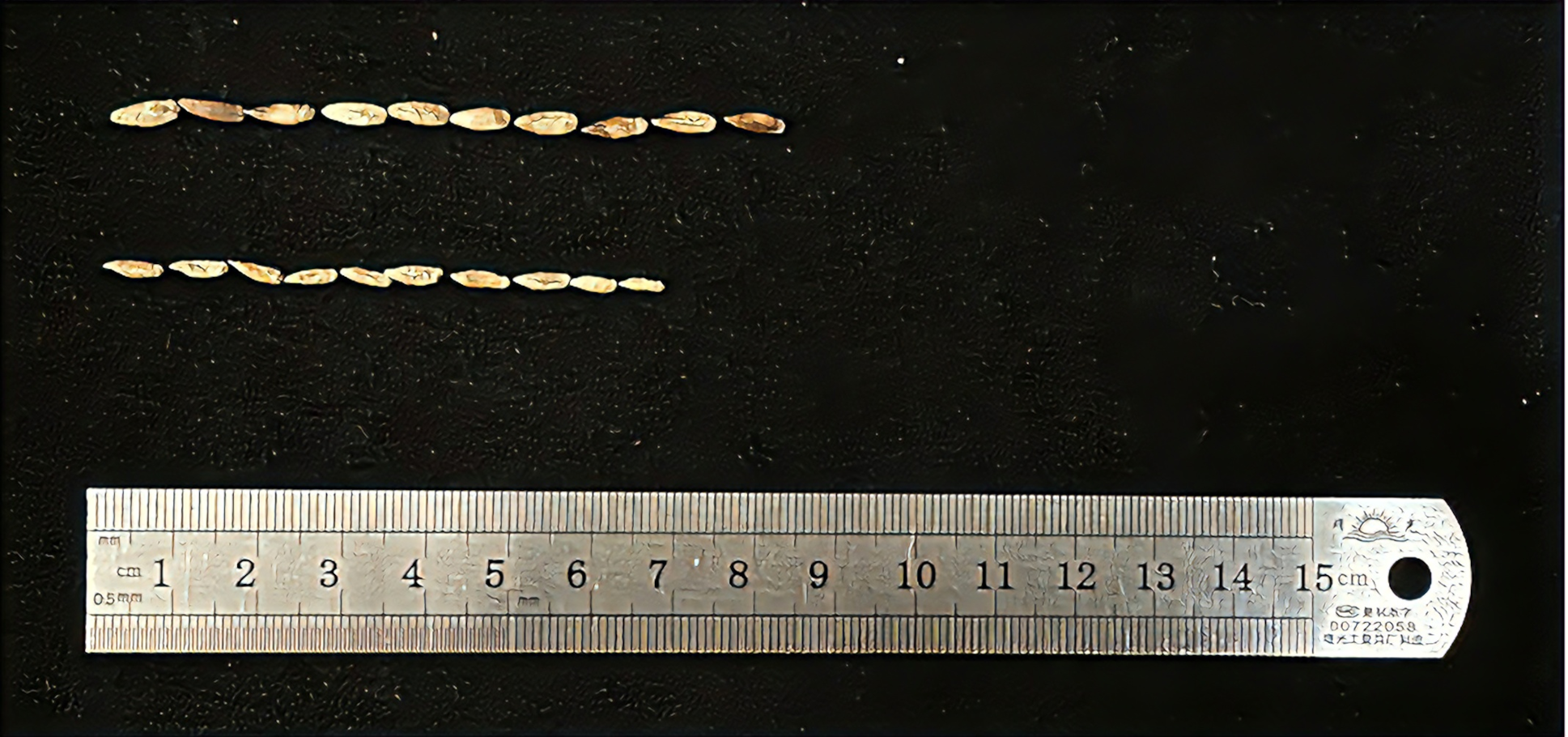

Supplement: Supplemental Information 12 [file peerj-14-20811-s012.png]
