# Supplementary figures and images for: Gene mapping and development of molecular markers for thousand-grain weight in rye based on bulked segregant analysis (part 1 of 2)
Source: PeerJ. 2026 Feb 12;14:e20811. doi: 10.7717/peerj.20811 (PMC12906707; doi:10.7717/peerj.20811)

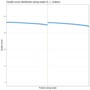

Supplement: Supplemental Information 1 [file peerj-14-20811-s001.zip › Supplementary 1/src/images/3_1_muben.quality_distribution.JPEG]

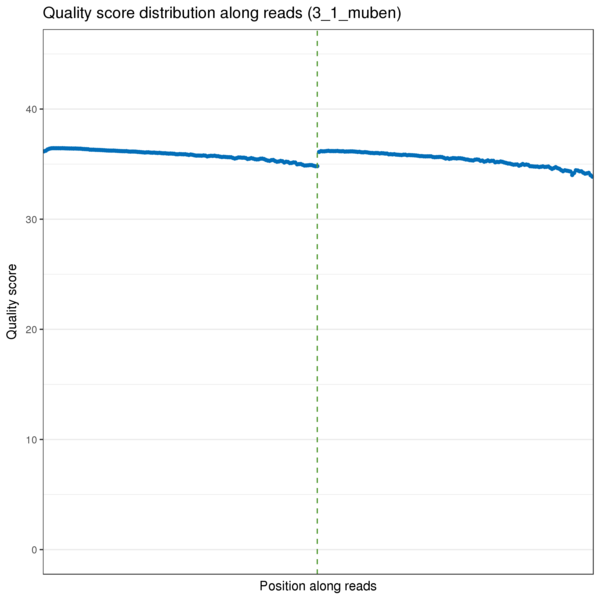

Supplement: Supplemental Information 1 [file peerj-14-20811-s001.zip › Supplementary 1/src/images/3_1_muben.quality_distribution.png]

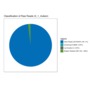

Supplement: Supplemental Information 1 [file peerj-14-20811-s001.zip › Supplementary 1/src/images/3_1_muben.raw_reads_classification.JPEG]

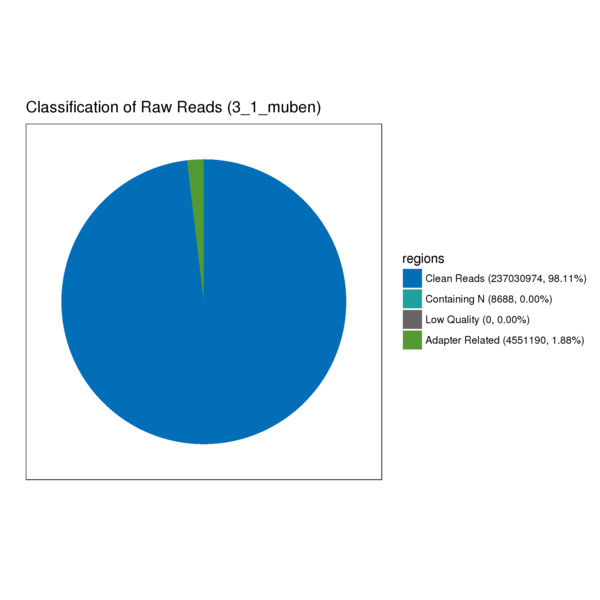

Supplement: Supplemental Information 1 [file peerj-14-20811-s001.zip › Supplementary 1/src/images/3_1_muben.raw_reads_classification.png]

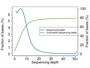

Supplement: Supplemental Information 1 [file peerj-14-20811-s001.zip › Supplementary 1/src/images/3_1_muben.Sequencing-depth.JPEG]

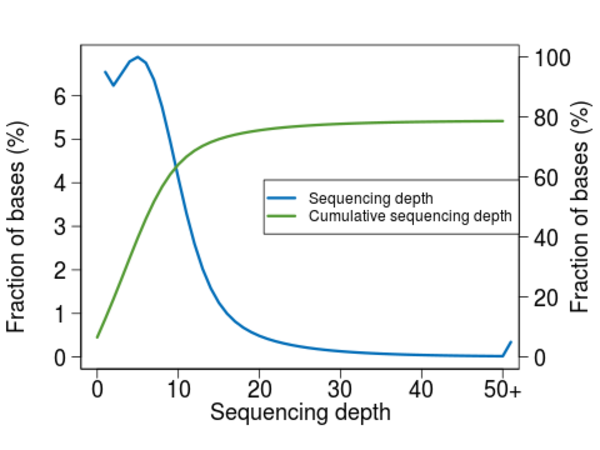

Supplement: Supplemental Information 1 [file peerj-14-20811-s001.zip › Supplementary 1/src/images/3_1_muben.Sequencing-depth.png]

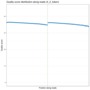

Supplement: Supplemental Information 1 [file peerj-14-20811-s001.zip › Supplementary 1/src/images/4_2_fuben.quality_distribution.JPEG]

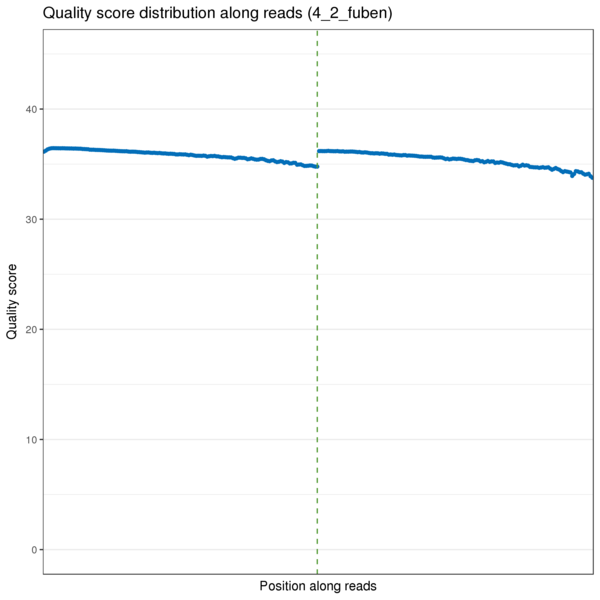

Supplement: Supplemental Information 1 [file peerj-14-20811-s001.zip › Supplementary 1/src/images/4_2_fuben.quality_distribution.png]

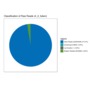

Supplement: Supplemental Information 1 [file peerj-14-20811-s001.zip › Supplementary 1/src/images/4_2_fuben.raw_reads_classification.JPEG]

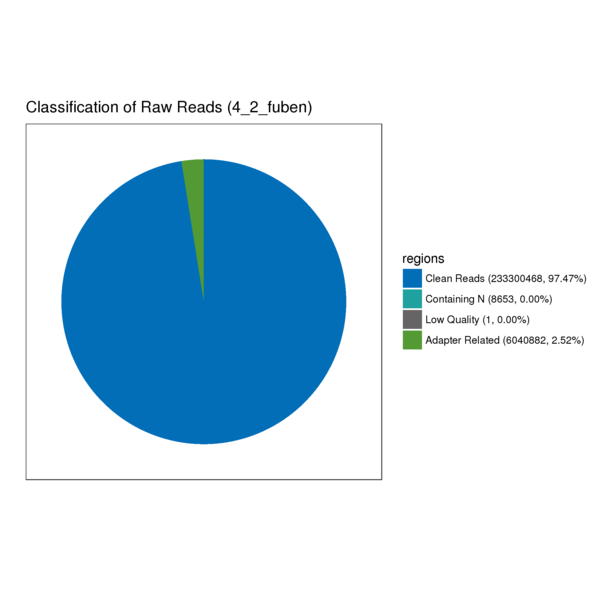

Supplement: Supplemental Information 1 [file peerj-14-20811-s001.zip › Supplementary 1/src/images/4_2_fuben.raw_reads_classification.png]

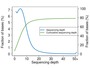

Supplement: Supplemental Information 1 [file peerj-14-20811-s001.zip › Supplementary 1/src/images/4_2_fuben.Sequencing-depth.JPEG]

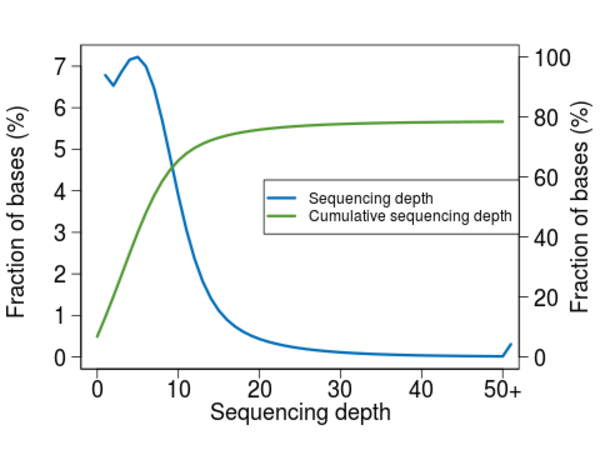

Supplement: Supplemental Information 1 [file peerj-14-20811-s001.zip › Supplementary 1/src/images/4_2_fuben.Sequencing-depth.png]

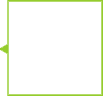

Supplement: Supplemental Information 1 [file peerj-14-20811-s001.zip › Supplementary 1/src/images/album-slider-arrow_box.png]

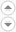

Supplement: Supplemental Information 1 [file peerj-14-20811-s001.zip › Supplementary 1/src/images/album-slider-button.png]

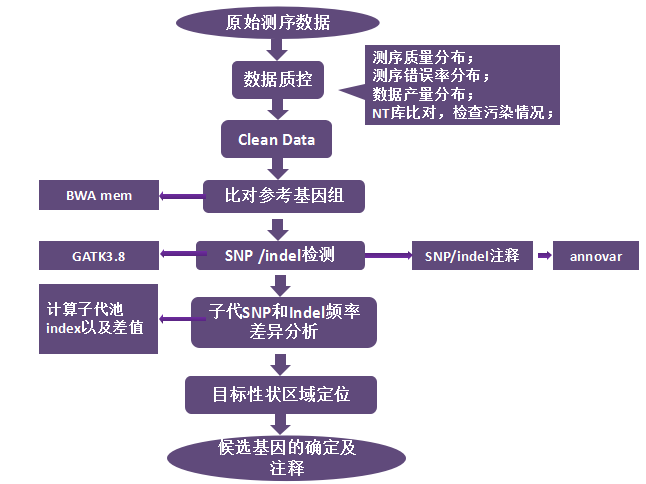

Supplement: Supplemental Information 1 [file peerj-14-20811-s001.zip › Supplementary 1/src/images/bioinfoWorkflow.png]

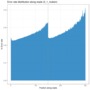

Supplement: Supplemental Information 1 [file peerj-14-20811-s001.zip › Supplementary 1/src/images/clean_3_1_muben.Error.JPEG]

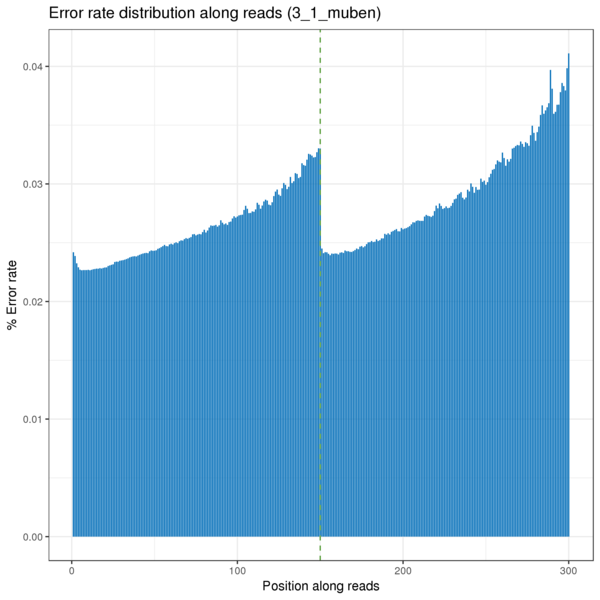

Supplement: Supplemental Information 1 [file peerj-14-20811-s001.zip › Supplementary 1/src/images/clean_3_1_muben.Error.png]

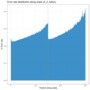

Supplement: Supplemental Information 1 [file peerj-14-20811-s001.zip › Supplementary 1/src/images/clean_4_2_fuben.Error.JPEG]

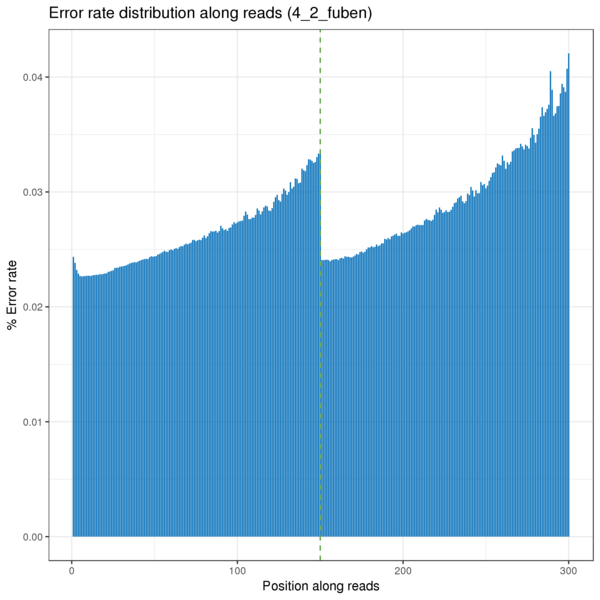

Supplement: Supplemental Information 1 [file peerj-14-20811-s001.zip › Supplementary 1/src/images/clean_4_2_fuben.Error.png]

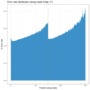

Supplement: Supplemental Information 1 [file peerj-14-20811-s001.zip › Supplementary 1/src/images/clean_High_f1.Error.JPEG]

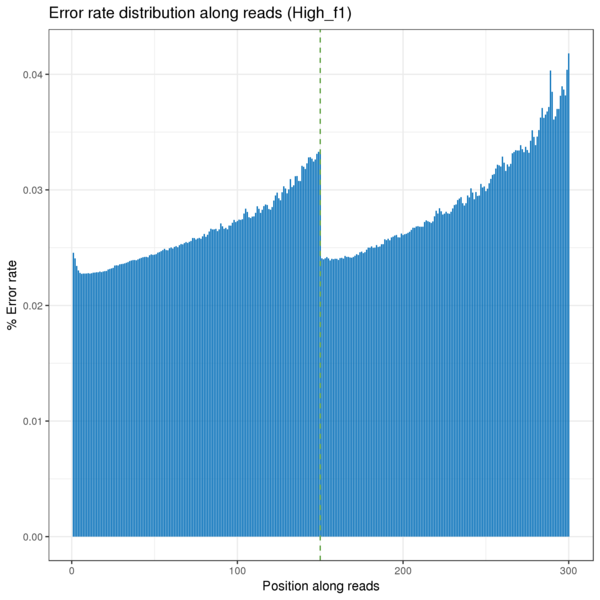

Supplement: Supplemental Information 1 [file peerj-14-20811-s001.zip › Supplementary 1/src/images/clean_High_f1.Error.png]

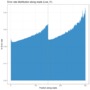

Supplement: Supplemental Information 1 [file peerj-14-20811-s001.zip › Supplementary 1/src/images/clean_Low_f1.Error.JPEG]

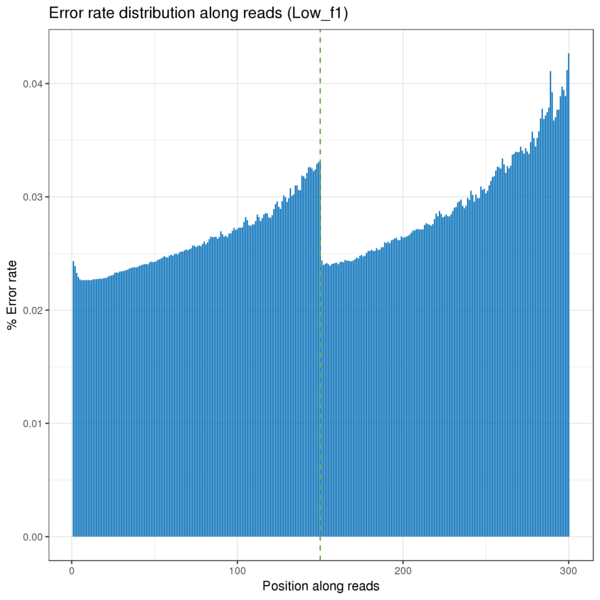

Supplement: Supplemental Information 1 [file peerj-14-20811-s001.zip › Supplementary 1/src/images/clean_Low_f1.Error.png]

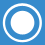

Supplement: Supplemental Information 1 [file peerj-14-20811-s001.zip › Supplementary 1/src/images/close.gif]

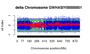

Supplement: Supplemental Information 1 [file peerj-14-20811-s001.zip › Supplementary 1/src/images/Delta_Allindex.GWHASIY00000001.JPEG]

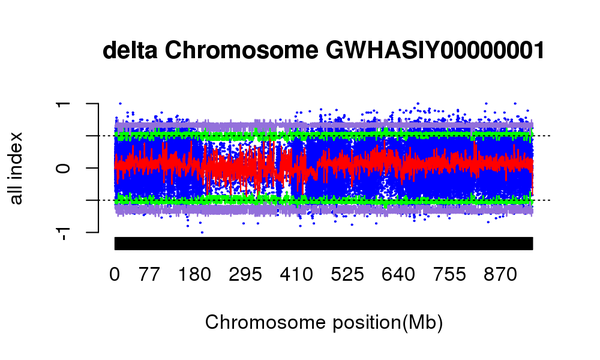

Supplement: Supplemental Information 1 [file peerj-14-20811-s001.zip › Supplementary 1/src/images/Delta_Allindex.GWHASIY00000001.png]

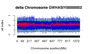

Supplement: Supplemental Information 1 [file peerj-14-20811-s001.zip › Supplementary 1/src/images/Delta_Allindex.GWHASIY00000002.JPEG]

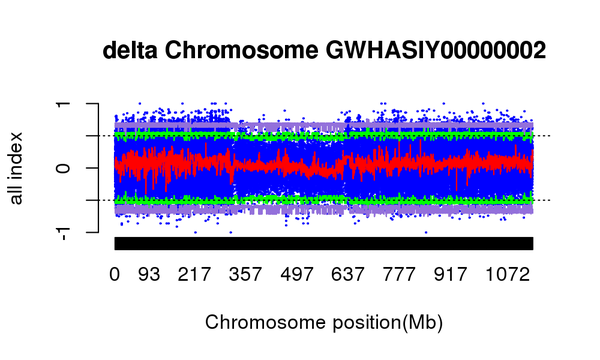

Supplement: Supplemental Information 1 [file peerj-14-20811-s001.zip › Supplementary 1/src/images/Delta_Allindex.GWHASIY00000002.png]

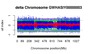

Supplement: Supplemental Information 1 [file peerj-14-20811-s001.zip › Supplementary 1/src/images/Delta_Allindex.GWHASIY00000003.JPEG]

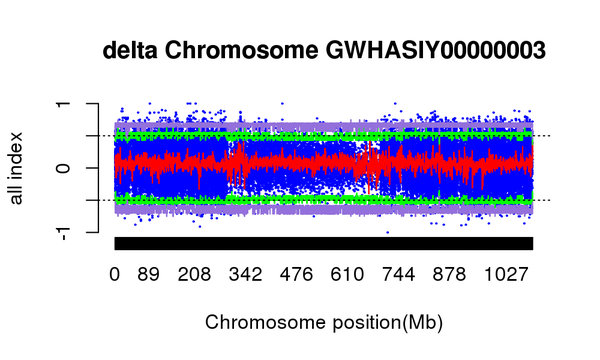

Supplement: Supplemental Information 1 [file peerj-14-20811-s001.zip › Supplementary 1/src/images/Delta_Allindex.GWHASIY00000003.png]

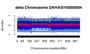

Supplement: Supplemental Information 1 [file peerj-14-20811-s001.zip › Supplementary 1/src/images/Delta_Allindex.GWHASIY00000004.JPEG]

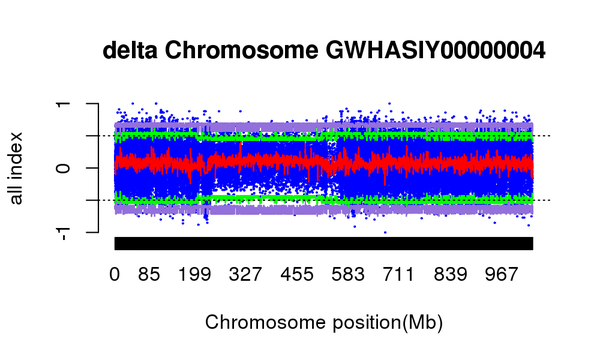

Supplement: Supplemental Information 1 [file peerj-14-20811-s001.zip › Supplementary 1/src/images/Delta_Allindex.GWHASIY00000004.png]

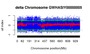

Supplement: Supplemental Information 1 [file peerj-14-20811-s001.zip › Supplementary 1/src/images/Delta_Allindex.GWHASIY00000005.JPEG]

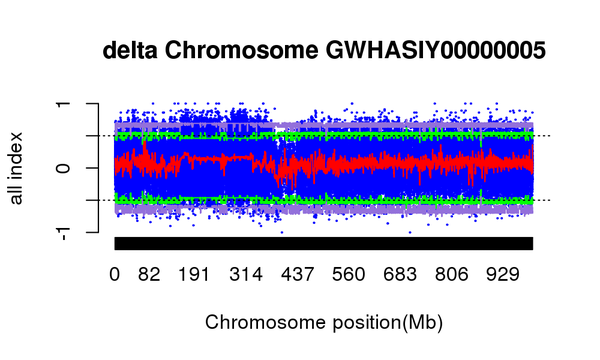

Supplement: Supplemental Information 1 [file peerj-14-20811-s001.zip › Supplementary 1/src/images/Delta_Allindex.GWHASIY00000005.png]

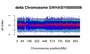

Supplement: Supplemental Information 1 [file peerj-14-20811-s001.zip › Supplementary 1/src/images/Delta_Allindex.GWHASIY00000006.JPEG]

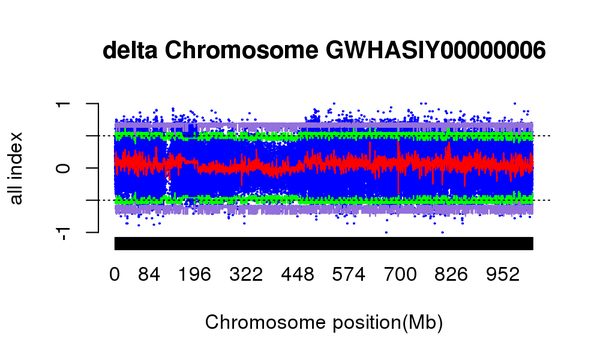

Supplement: Supplemental Information 1 [file peerj-14-20811-s001.zip › Supplementary 1/src/images/Delta_Allindex.GWHASIY00000006.png]

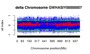

Supplement: Supplemental Information 1 [file peerj-14-20811-s001.zip › Supplementary 1/src/images/Delta_Allindex.GWHASIY00000007.JPEG]

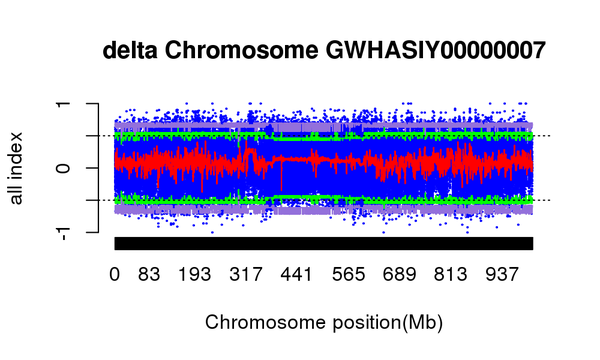

Supplement: Supplemental Information 1 [file peerj-14-20811-s001.zip › Supplementary 1/src/images/Delta_Allindex.GWHASIY00000007.png]

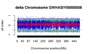

Supplement: Supplemental Information 1 [file peerj-14-20811-s001.zip › Supplementary 1/src/images/Delta_Allindex.GWHASIY00000008.JPEG]

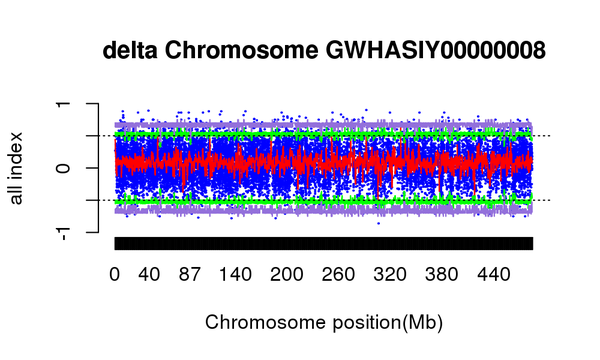

Supplement: Supplemental Information 1 [file peerj-14-20811-s001.zip › Supplementary 1/src/images/Delta_Allindex.GWHASIY00000008.png]

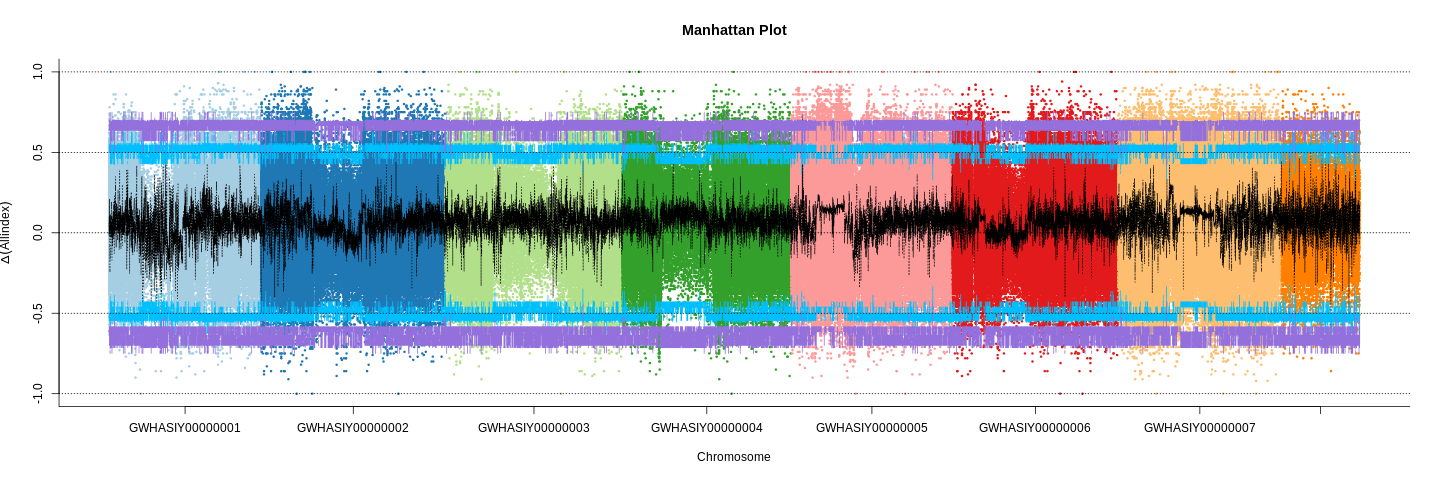

Supplement: Supplemental Information 1 [file peerj-14-20811-s001.zip › Supplementary 1/src/images/Delta_Allindex.manhattan.png]

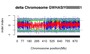

Supplement: Supplemental Information 1 [file peerj-14-20811-s001.zip › Supplementary 1/src/images/Delta_InDelindex.GWHASIY00000001.JPEG]

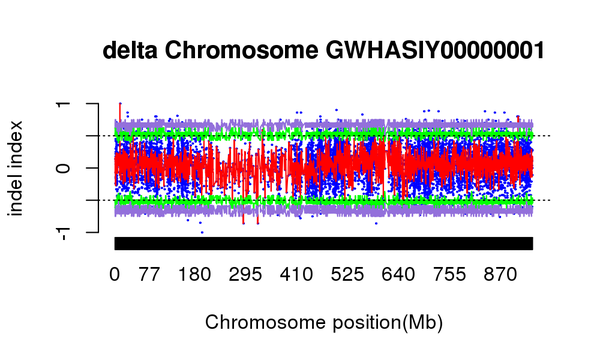

Supplement: Supplemental Information 1 [file peerj-14-20811-s001.zip › Supplementary 1/src/images/Delta_InDelindex.GWHASIY00000001.png]

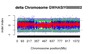

Supplement: Supplemental Information 1 [file peerj-14-20811-s001.zip › Supplementary 1/src/images/Delta_InDelindex.GWHASIY00000002.JPEG]

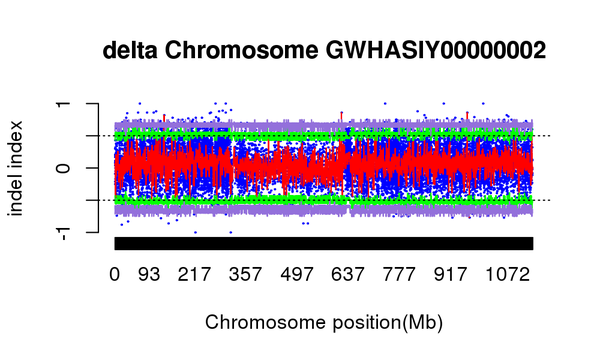

Supplement: Supplemental Information 1 [file peerj-14-20811-s001.zip › Supplementary 1/src/images/Delta_InDelindex.GWHASIY00000002.png]

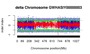

Supplement: Supplemental Information 1 [file peerj-14-20811-s001.zip › Supplementary 1/src/images/Delta_InDelindex.GWHASIY00000003.JPEG]

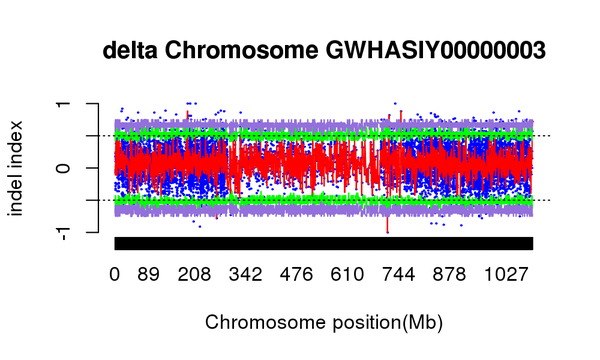

Supplement: Supplemental Information 1 [file peerj-14-20811-s001.zip › Supplementary 1/src/images/Delta_InDelindex.GWHASIY00000003.png]

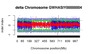

Supplement: Supplemental Information 1 [file peerj-14-20811-s001.zip › Supplementary 1/src/images/Delta_InDelindex.GWHASIY00000004.JPEG]

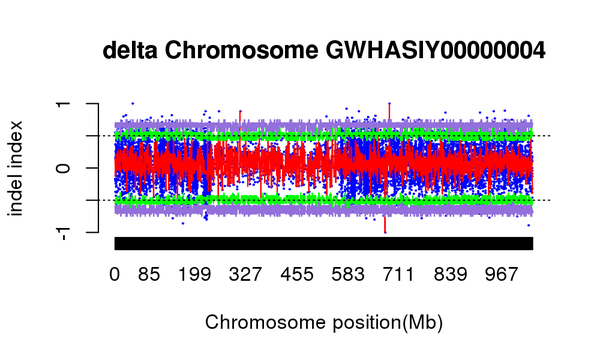

Supplement: Supplemental Information 1 [file peerj-14-20811-s001.zip › Supplementary 1/src/images/Delta_InDelindex.GWHASIY00000004.png]

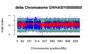

Supplement: Supplemental Information 1 [file peerj-14-20811-s001.zip › Supplementary 1/src/images/Delta_InDelindex.GWHASIY00000005.JPEG]

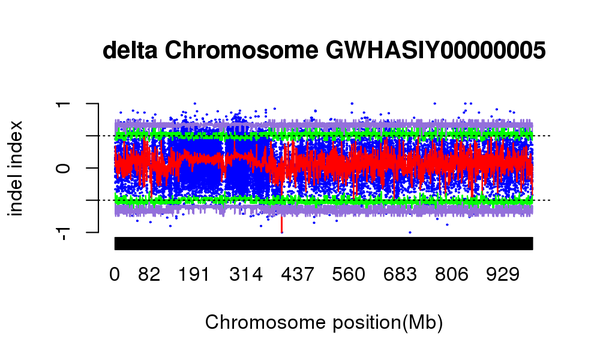

Supplement: Supplemental Information 1 [file peerj-14-20811-s001.zip › Supplementary 1/src/images/Delta_InDelindex.GWHASIY00000005.png]

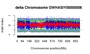

Supplement: Supplemental Information 1 [file peerj-14-20811-s001.zip › Supplementary 1/src/images/Delta_InDelindex.GWHASIY00000006.JPEG]

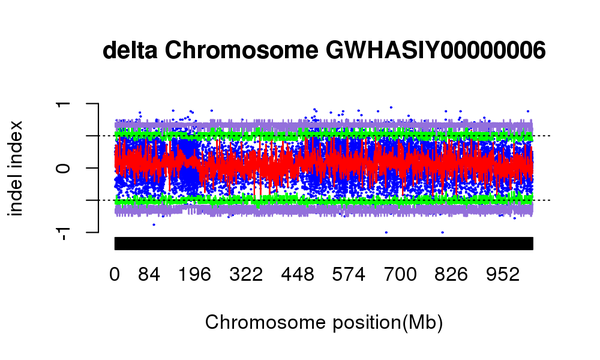

Supplement: Supplemental Information 1 [file peerj-14-20811-s001.zip › Supplementary 1/src/images/Delta_InDelindex.GWHASIY00000006.png]

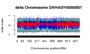

Supplement: Supplemental Information 1 [file peerj-14-20811-s001.zip › Supplementary 1/src/images/Delta_InDelindex.GWHASIY00000007.JPEG]

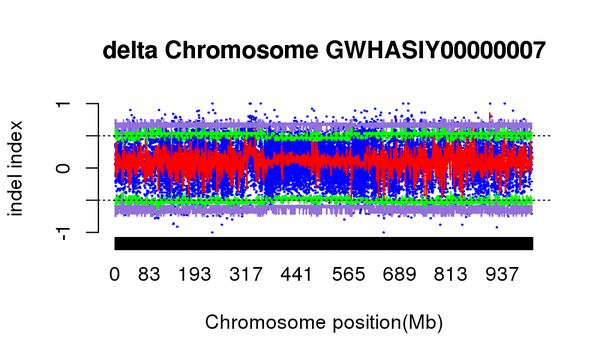

Supplement: Supplemental Information 1 [file peerj-14-20811-s001.zip › Supplementary 1/src/images/Delta_InDelindex.GWHASIY00000007.png]

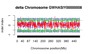

Supplement: Supplemental Information 1 [file peerj-14-20811-s001.zip › Supplementary 1/src/images/Delta_InDelindex.GWHASIY00000008.JPEG]

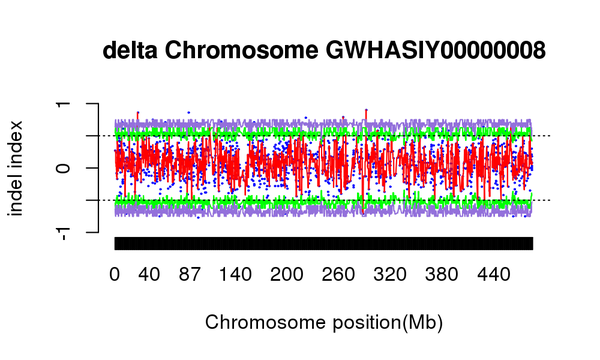

Supplement: Supplemental Information 1 [file peerj-14-20811-s001.zip › Supplementary 1/src/images/Delta_InDelindex.GWHASIY00000008.png]

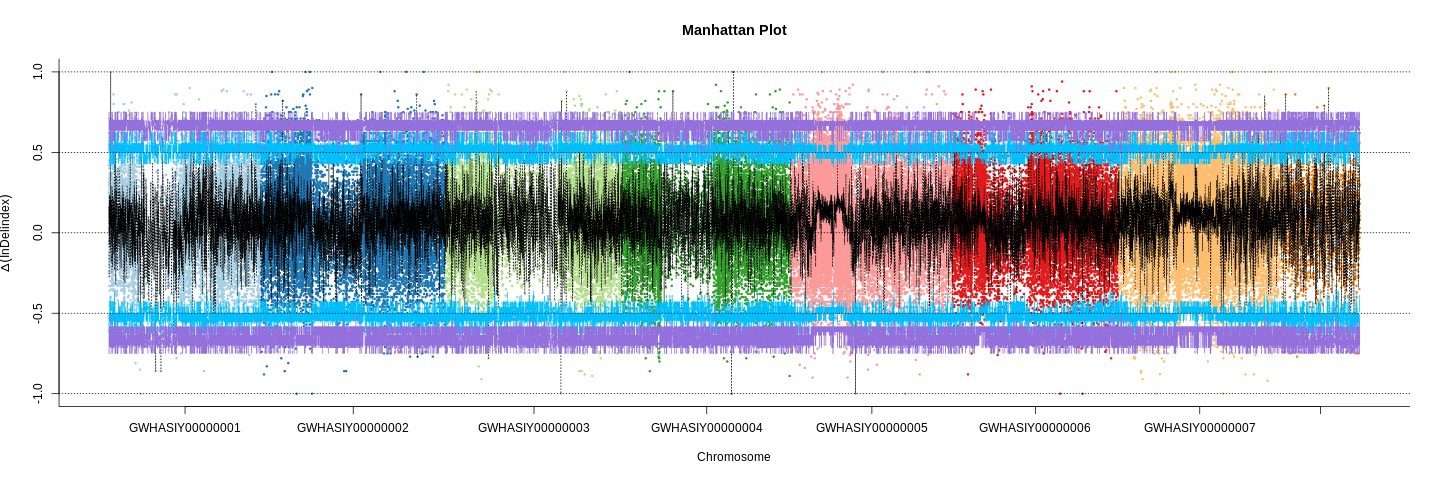

Supplement: Supplemental Information 1 [file peerj-14-20811-s001.zip › Supplementary 1/src/images/Delta_InDelindex.manhattan.png]

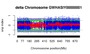

Supplement: Supplemental Information 1 [file peerj-14-20811-s001.zip › Supplementary 1/src/images/Delta_SNPindex.GWHASIY00000001.JPEG]

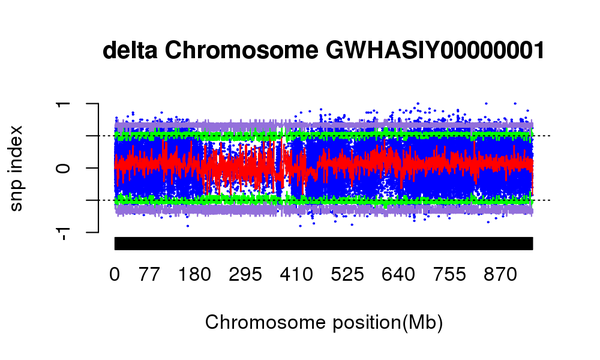

Supplement: Supplemental Information 1 [file peerj-14-20811-s001.zip › Supplementary 1/src/images/Delta_SNPindex.GWHASIY00000001.png]

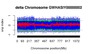

Supplement: Supplemental Information 1 [file peerj-14-20811-s001.zip › Supplementary 1/src/images/Delta_SNPindex.GWHASIY00000002.JPEG]

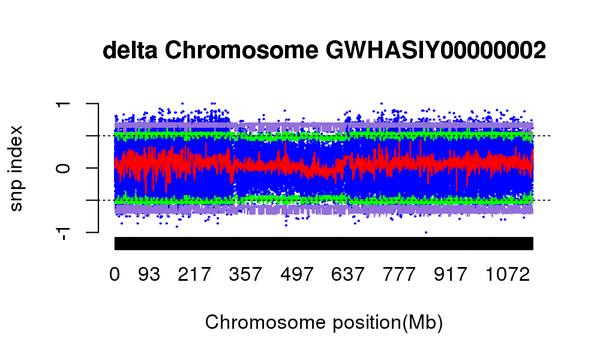

Supplement: Supplemental Information 1 [file peerj-14-20811-s001.zip › Supplementary 1/src/images/Delta_SNPindex.GWHASIY00000002.png]

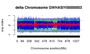

Supplement: Supplemental Information 1 [file peerj-14-20811-s001.zip › Supplementary 1/src/images/Delta_SNPindex.GWHASIY00000003.JPEG]

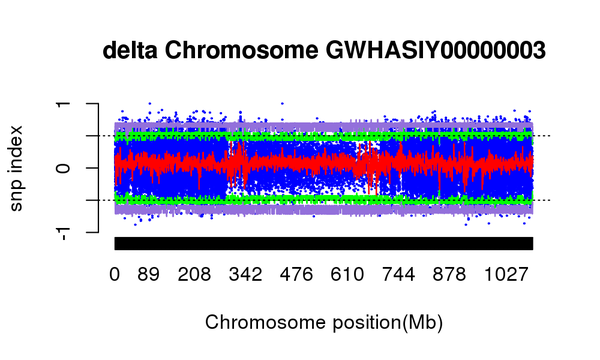

Supplement: Supplemental Information 1 [file peerj-14-20811-s001.zip › Supplementary 1/src/images/Delta_SNPindex.GWHASIY00000003.png]

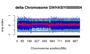

Supplement: Supplemental Information 1 [file peerj-14-20811-s001.zip › Supplementary 1/src/images/Delta_SNPindex.GWHASIY00000004.JPEG]

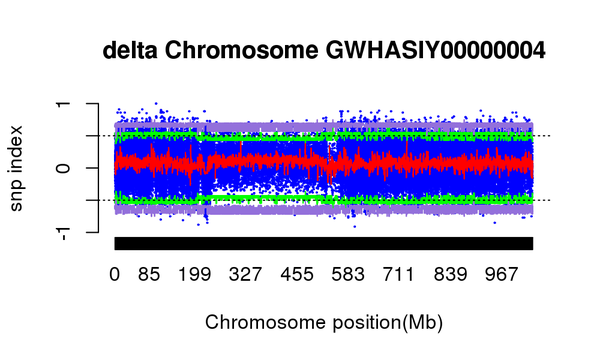

Supplement: Supplemental Information 1 [file peerj-14-20811-s001.zip › Supplementary 1/src/images/Delta_SNPindex.GWHASIY00000004.png]

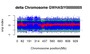

Supplement: Supplemental Information 1 [file peerj-14-20811-s001.zip › Supplementary 1/src/images/Delta_SNPindex.GWHASIY00000005.JPEG]

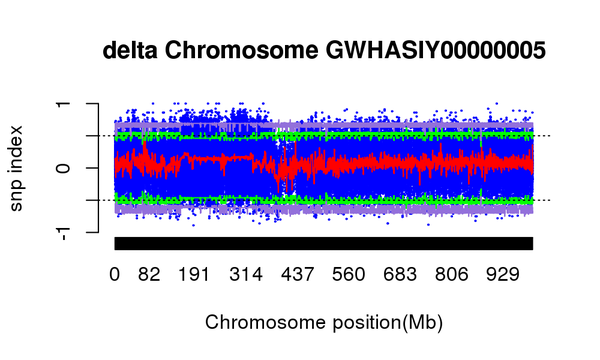

Supplement: Supplemental Information 1 [file peerj-14-20811-s001.zip › Supplementary 1/src/images/Delta_SNPindex.GWHASIY00000005.png]

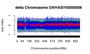

Supplement: Supplemental Information 1 [file peerj-14-20811-s001.zip › Supplementary 1/src/images/Delta_SNPindex.GWHASIY00000006.JPEG]

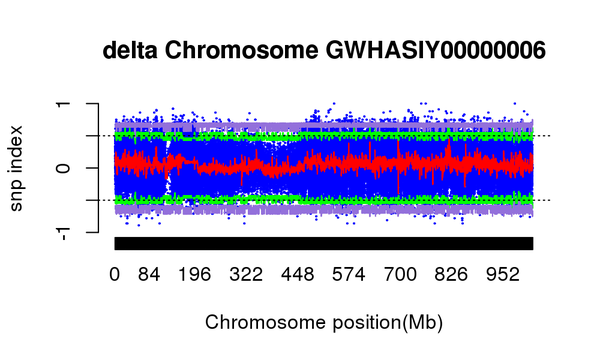

Supplement: Supplemental Information 1 [file peerj-14-20811-s001.zip › Supplementary 1/src/images/Delta_SNPindex.GWHASIY00000006.png]

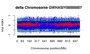

Supplement: Supplemental Information 1 [file peerj-14-20811-s001.zip › Supplementary 1/src/images/Delta_SNPindex.GWHASIY00000007.JPEG]

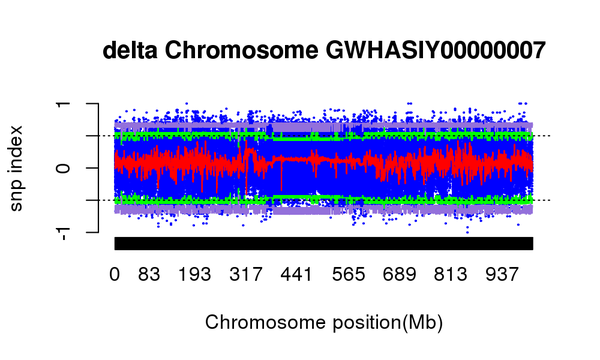

Supplement: Supplemental Information 1 [file peerj-14-20811-s001.zip › Supplementary 1/src/images/Delta_SNPindex.GWHASIY00000007.png]

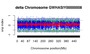

Supplement: Supplemental Information 1 [file peerj-14-20811-s001.zip › Supplementary 1/src/images/Delta_SNPindex.GWHASIY00000008.JPEG]

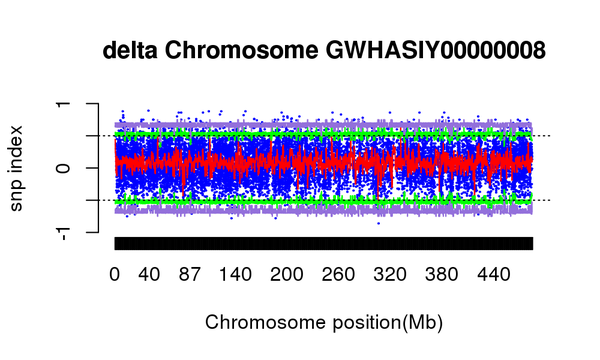

Supplement: Supplemental Information 1 [file peerj-14-20811-s001.zip › Supplementary 1/src/images/Delta_SNPindex.GWHASIY00000008.png]

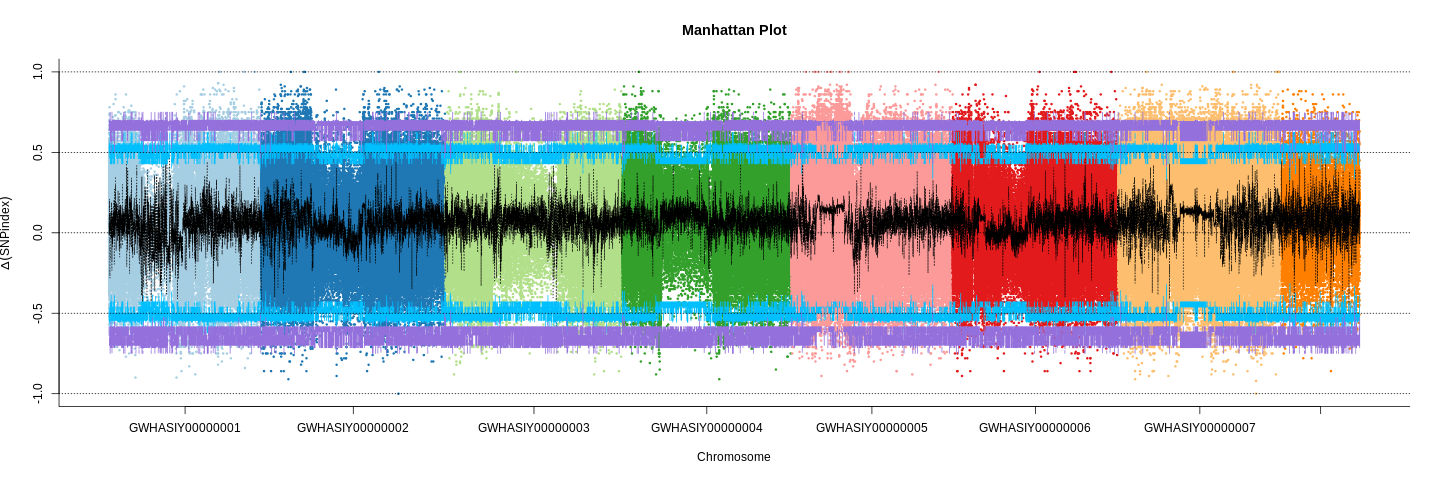

Supplement: Supplemental Information 1 [file peerj-14-20811-s001.zip › Supplementary 1/src/images/Delta_SNPindex.manhattan.png]

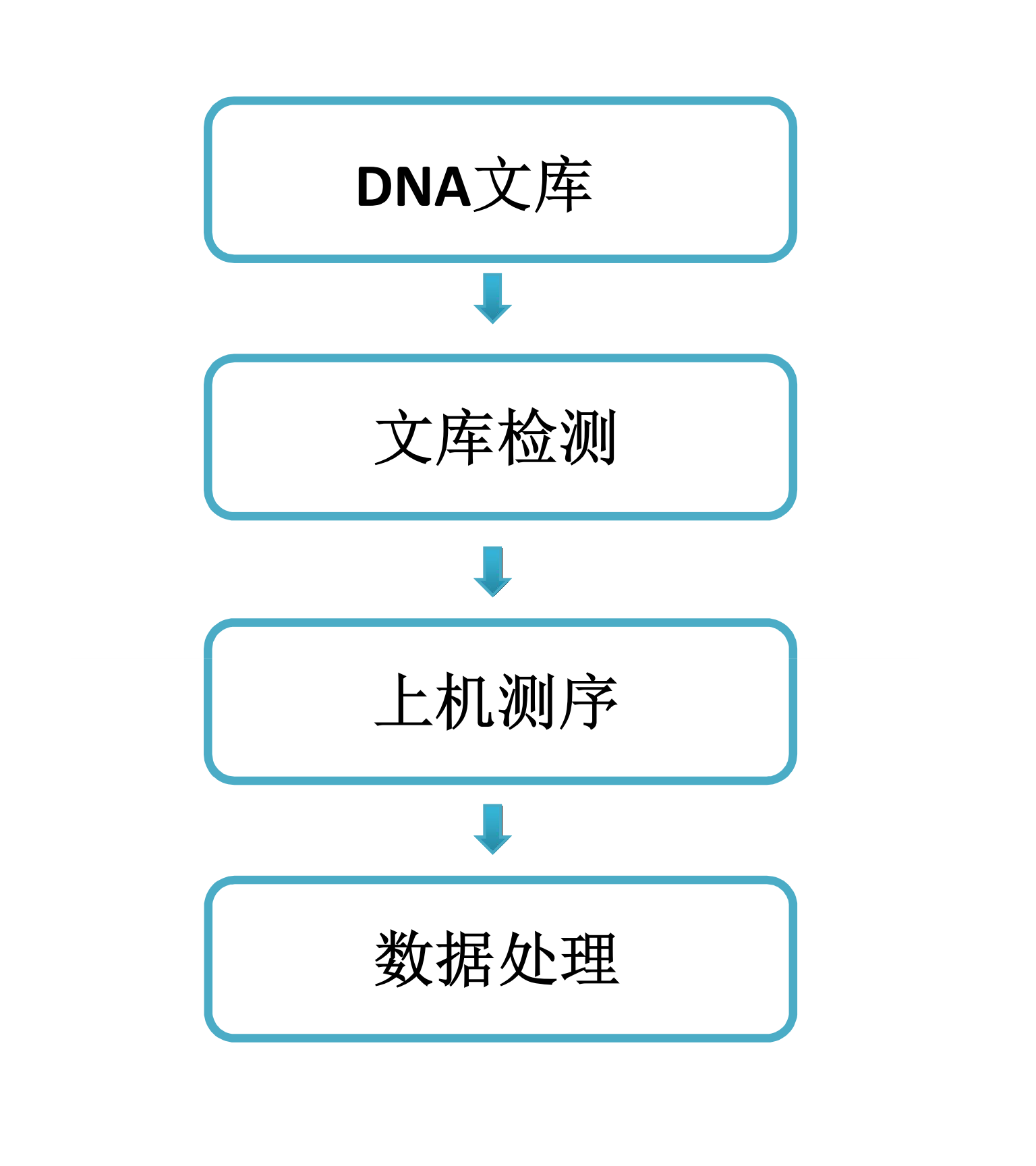

Supplement: Supplemental Information 1 [file peerj-14-20811-s001.zip › Supplementary 1/src/images/exp_pipeline.png]

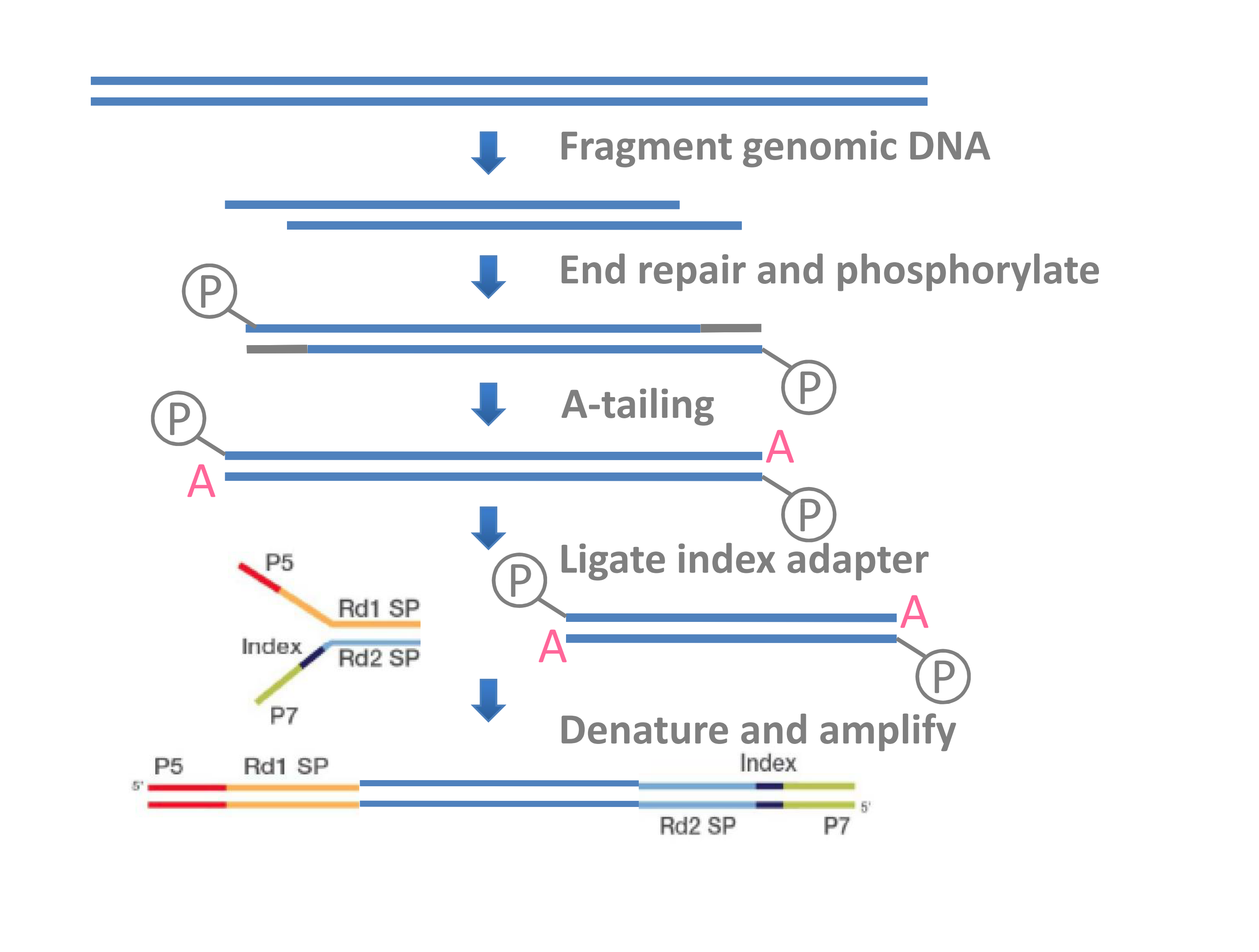

Supplement: Supplemental Information 1 [file peerj-14-20811-s001.zip › Supplementary 1/src/images/exp_pipeline_1.png]

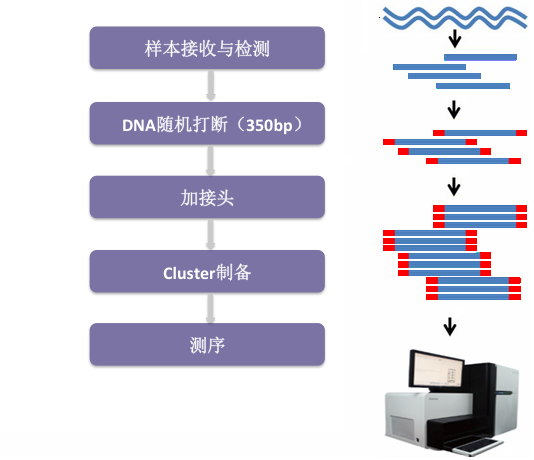

Supplement: Supplemental Information 1 [file peerj-14-20811-s001.zip › Supplementary 1/src/images/experimentWorkflow.png]

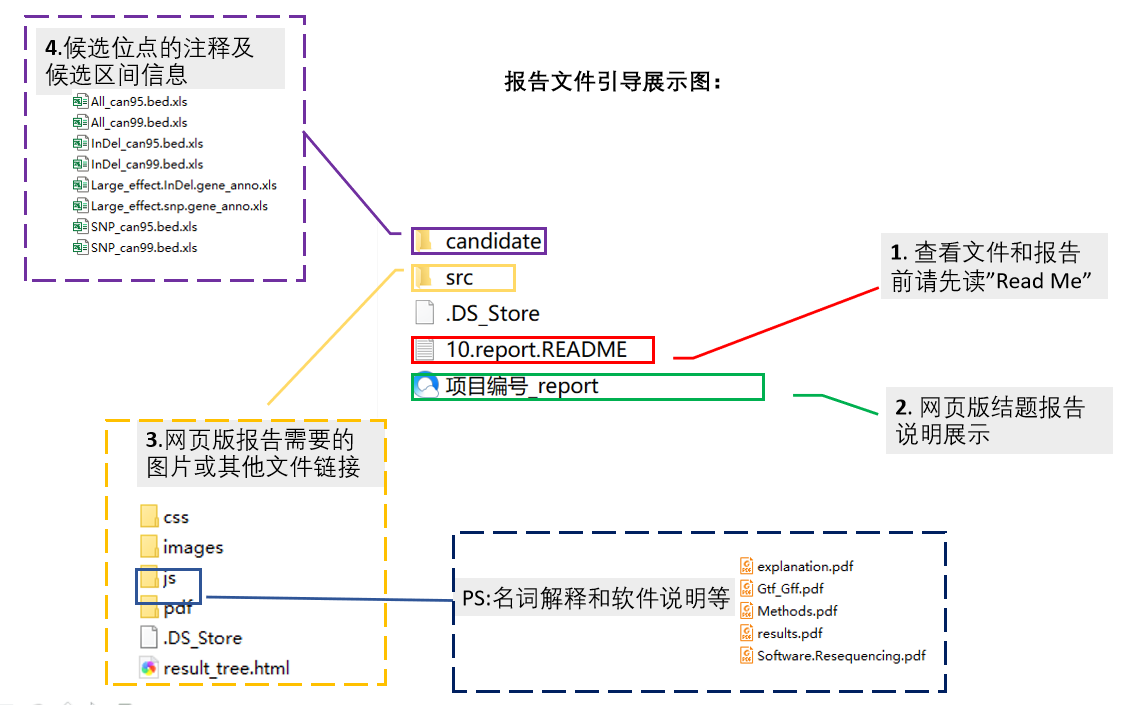

Supplement: Supplemental Information 1 [file peerj-14-20811-s001.zip › Supplementary 1/src/images/files_user.png]

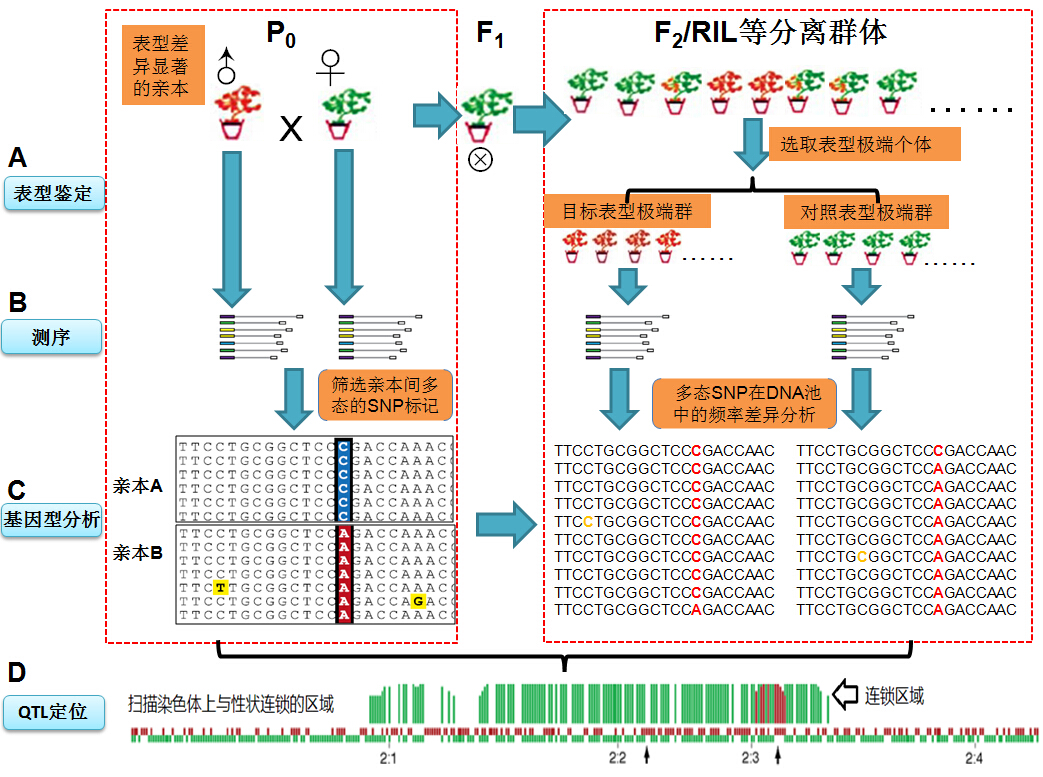

Supplement: Supplemental Information 1 [file peerj-14-20811-s001.zip › Supplementary 1/src/images/flow.jpg]

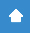

Supplement: Supplemental Information 1 [file peerj-14-20811-s001.zip › Supplementary 1/src/images/goTop.jpg]

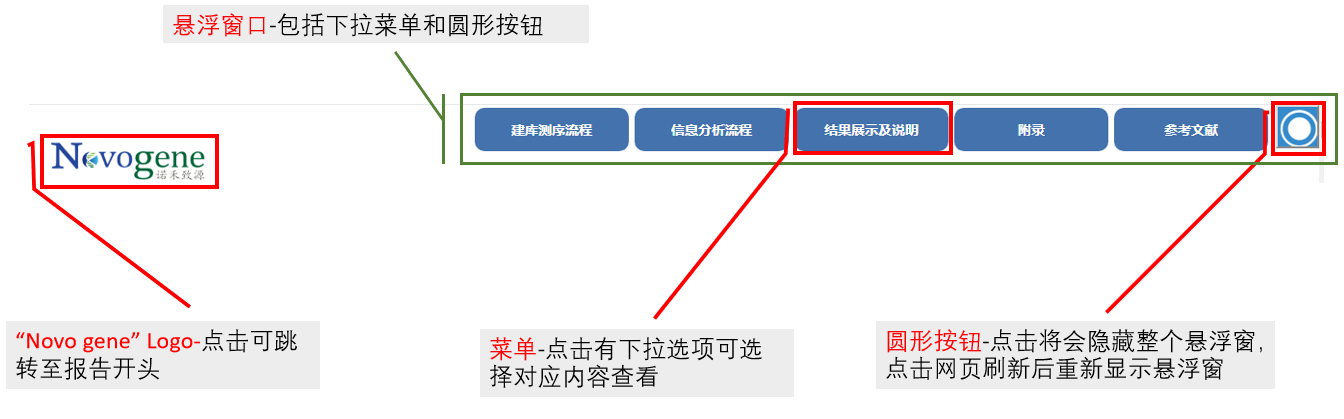

Supplement: Supplemental Information 1 [file peerj-14-20811-s001.zip › Supplementary 1/src/images/head_order.png]

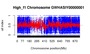

Supplement: Supplemental Information 1 [file peerj-14-20811-s001.zip › Supplementary 1/src/images/High_f1.All_index.GWHASIY00000001.JPEG]

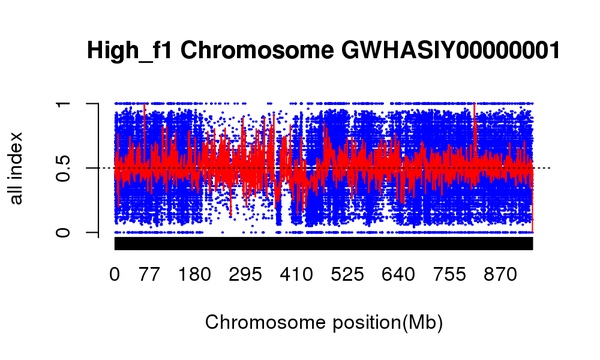

Supplement: Supplemental Information 1 [file peerj-14-20811-s001.zip › Supplementary 1/src/images/High_f1.All_index.GWHASIY00000001.png]

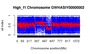

Supplement: Supplemental Information 1 [file peerj-14-20811-s001.zip › Supplementary 1/src/images/High_f1.All_index.GWHASIY00000002.JPEG]

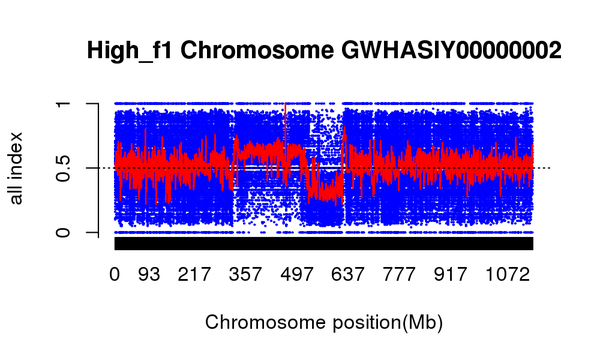

Supplement: Supplemental Information 1 [file peerj-14-20811-s001.zip › Supplementary 1/src/images/High_f1.All_index.GWHASIY00000002.png]

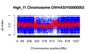

Supplement: Supplemental Information 1 [file peerj-14-20811-s001.zip › Supplementary 1/src/images/High_f1.All_index.GWHASIY00000003.JPEG]

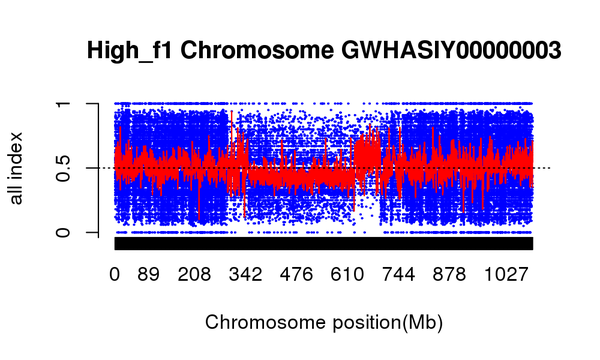

Supplement: Supplemental Information 1 [file peerj-14-20811-s001.zip › Supplementary 1/src/images/High_f1.All_index.GWHASIY00000003.png]

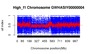

Supplement: Supplemental Information 1 [file peerj-14-20811-s001.zip › Supplementary 1/src/images/High_f1.All_index.GWHASIY00000004.JPEG]

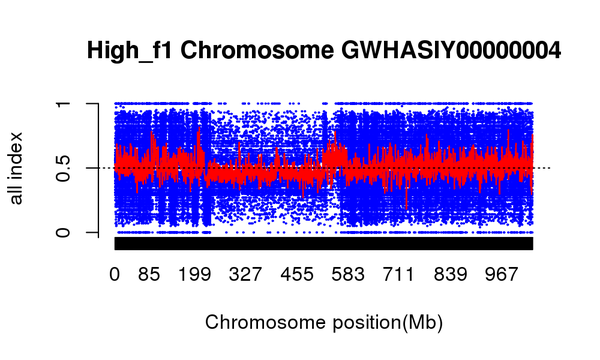

Supplement: Supplemental Information 1 [file peerj-14-20811-s001.zip › Supplementary 1/src/images/High_f1.All_index.GWHASIY00000004.png]

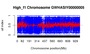

Supplement: Supplemental Information 1 [file peerj-14-20811-s001.zip › Supplementary 1/src/images/High_f1.All_index.GWHASIY00000005.JPEG]

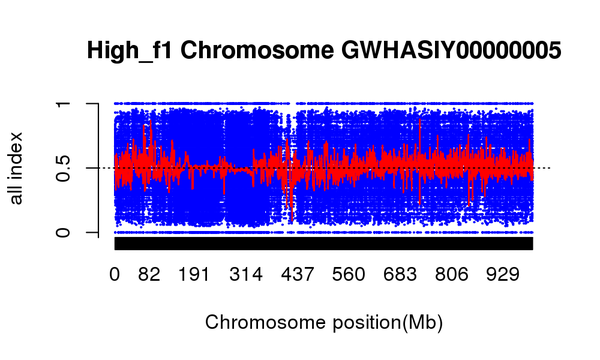

Supplement: Supplemental Information 1 [file peerj-14-20811-s001.zip › Supplementary 1/src/images/High_f1.All_index.GWHASIY00000005.png]

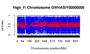

Supplement: Supplemental Information 1 [file peerj-14-20811-s001.zip › Supplementary 1/src/images/High_f1.All_index.GWHASIY00000006.JPEG]

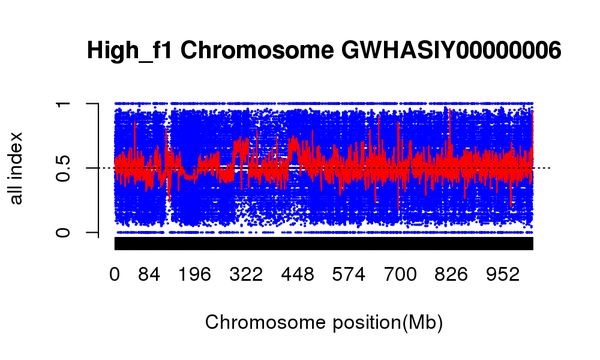

Supplement: Supplemental Information 1 [file peerj-14-20811-s001.zip › Supplementary 1/src/images/High_f1.All_index.GWHASIY00000006.png]

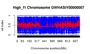

Supplement: Supplemental Information 1 [file peerj-14-20811-s001.zip › Supplementary 1/src/images/High_f1.All_index.GWHASIY00000007.JPEG]

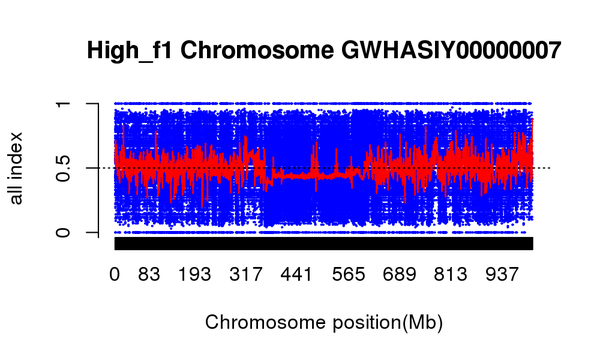

Supplement: Supplemental Information 1 [file peerj-14-20811-s001.zip › Supplementary 1/src/images/High_f1.All_index.GWHASIY00000007.png]

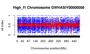

Supplement: Supplemental Information 1 [file peerj-14-20811-s001.zip › Supplementary 1/src/images/High_f1.All_index.GWHASIY00000008.JPEG]

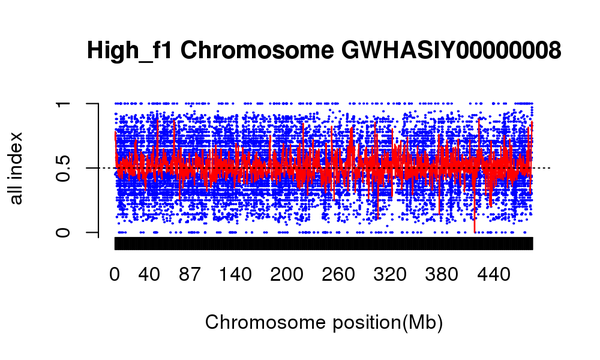

Supplement: Supplemental Information 1 [file peerj-14-20811-s001.zip › Supplementary 1/src/images/High_f1.All_index.GWHASIY00000008.png]

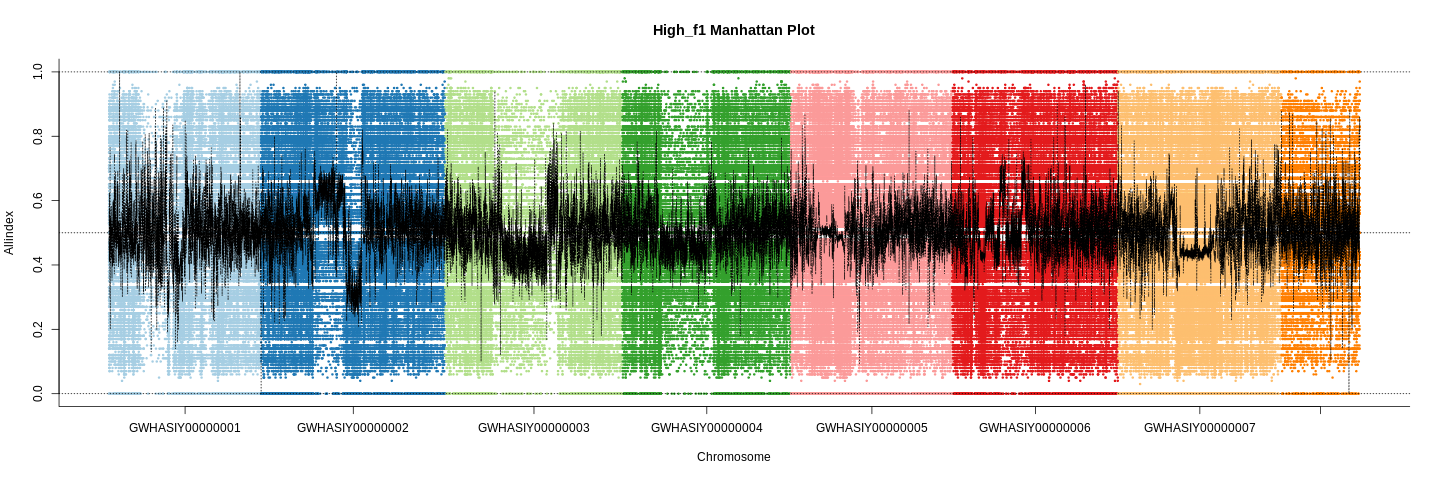

Supplement: Supplemental Information 1 [file peerj-14-20811-s001.zip › Supplementary 1/src/images/High_f1.All_index.manhattan.png]

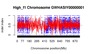

Supplement: Supplemental Information 1 [file peerj-14-20811-s001.zip › Supplementary 1/src/images/High_f1.InDel_index.GWHASIY00000001.JPEG]
