# Supplementary material for: Gene mapping and development of molecular markers for thousand-grain weight in rye based on bulked segregant analysis
Source: PeerJ. 2026 Feb 12;14:e20811. doi: 10.7717/peerj.20811 (PMC12906707; doi:10.7717/peerj.20811)
Supplement: Supplemental Information 1 [file peerj-14-20811-s001.zip › Supplementary 1/src/pdf/explanation.pdf]

动植物重测序名词解释

诺禾致源动植物重测序业务线

重测序基础概念

1、高通量测序：高通量测序技术（High-throughput sequencing，HTS）是对传统 Sanger 测序（称为一代测序技术）革命性的改变，一次对几十万到几百万条核酸分子进行序列测定，又称为下一代测序技术(next generation sequencing，NGS)。

2、全基因组重测序（WGS）：全基因组重测序是对基因组序列已知的个体进行基因组测序，并在个体或群体水平上进行差异性分析的方法。

3、简化基因组测序（GBS）：通过 *Mse* I、*EcoR* I、*Nla* III、*Hae* III 等单酶或组合酶切进行简化，获取酶切位点附件的序列信息，捕获区域为全基因组的 1%左右。

4、SNP：单核苷酸多态性（single nucleotide polymorphism），主要是指在基因组水平上由单个核苷酸的变异所引起的 DNA 序列多态性，包括单个碱基的转换、颠换等。

5、InDel：插入/缺失（Insertion/Deletion），基因组中小片段（1-50bp）的插入和缺失序列。

6、SV：structure variation： 基因组结构变异，染色体结构变异是指在染色体上发生了大片段的变异。主要包括染色体大片段的插入和缺失（引起 CNV 的变化），染色体内部的某块区域发生重复复制、 翻转颠换、易位、 两条染色体之间发生重组（ inter-chromosome trans-location）等。

7、CNV：copy number variation：基因组拷贝数变异，是基因组变异的一种形式，通常使基因组中大片段的 DNA 形成非正常的拷贝数量。

8、样本比对率（mapping rate）：比对到参考基因组上的 reads 数目除以有效测序数据的 reads 数目，反映了样本测序数据与参考基因组的相似性。

9、基因组测序深度： 测序得到的总碱基数与待测基因组大小的比值。如测一个物种的全基因组，基因组大小约为 5G，测序获得 100G 的数据量，则测序深度为 20X。

10、基因组覆盖率： 指测序获得的序列占整个基因组的比例。由于基因组中的高 GC、重复序列等复杂结构的存在，测序最终拼接组装获得的序列往往无法覆盖有所的区域，这部分没有获得的区域就称为 Gap。

11、GC%： GC 含量，全基因组范围内或在特定基因组序列内的 4 种碱基中，鸟嘌呤和胞嘧啶所占的比率。

Unmapped reads 基础概念

1、Unmapped reads:100 条 reads，85 条可以 mapping 到参考基因组，mapping 就是 85%；剩余的 15 条 reads，就是 unmapped reads。

2、Unmapped reads 组装：通过对个体或群体进行全基因组高深度重测序，将每个个体的测序数据与参考基因组进行比对。对所获得的 Unmapped reads，通过组装软件进行局部拼接，拼接得到的 contigs 与数据库进行比对，进行基因结构预测及基因功能注释。

建库测序相关

1、 adapter：接头，用于上机测序。建库时引入的接头序列与测序芯片（flow cell）上固定的接头相互识别。

2、index：测序的标签，用于测定混合样本，通过每个样本添加的不同标签进行数据区分，鉴别测序样品。

3、Q20,Q30：Phred 数值大于 20、30 的碱基占总体碱基的百分比，其中 Phred=-10log10(e), e 为碱基错误率.

4、raw data/raw reads：测序下机的原始数据。

5、clean data/clean reads：对原始数据进行过滤后，剔除了低质量数据的剩余数据。后续分析均基于 clean data。

6、read：高通量测序平台产生的序列标签就称为 read。

BSA 性状定位基础概念

1、BSA：混合分组分析法（Bulked Segregant Analysis，BSA），是一种检测位于特定染色体区段上标记的方法。简单地讲，对研究的目标性状，选择表型差异显著的亲本构建家系群体，再从家系群体中选取目标性状表型极端的一定数量的单株，混合构建两个 DNA“池”（DNA pools）。两池间的 DNA 差异片段即为候选区域，所关注的基因或者 QTL 可能位于该候选区域中。

2、Genotype and phenotype： 基因型与表型， 基因型是指某一生物个体全部基因组合的总称；表型，又称性状，是基因型和环境共同作用的结果。

3、质量性状：指属性性状，是指同一种性状的不同表现型之间不存在连续性的数量变化，而呈现质的中断性变化的那些性状。

4、数量性状：是指在一个群体内的各个体间表现为连续变异的性状，如动植物的高度或长度等。数量性状较易受环境的影响，在一个群体内各个个体的差异一般呈连续的正态分布，难以在个体间明确地分组。

5、初定位：对目标性状进行粗略的定位，定位的遗传距离和物理距离一般比较大。

6、精细定位：是在初定位的基础上，进一步缩小初定位的结果。EMS 人工诱变的性状一般为质量性状，可以做到精细定位，自然突变的性状多为数量性状，适合做初定位分析。

7、家系群体：植物育种上的家系是指从群体中选出的某一个个体自交产生的后代，或由于 2 个个体杂交产生的后代，或由一个个体产生的后代（根据母本而不是根据父本来判别）等，通过不同程度的同系交配产生的家系。

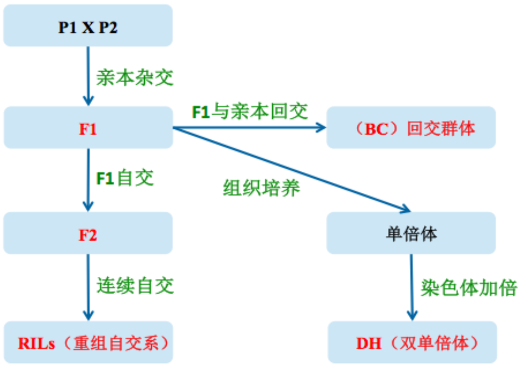

遗传图谱基础概念

1、QTL：是 quantitative trait locus 的缩写，数量性状座位或者数量性状基因座，它指的是控制数量性状的基因在基因组中的位置。

2、遗传图谱：是指基因或 DNA 标记在染色体上的相对位置与遗传距离。单位：由基因或 DNA 片段在染色体交换过程中分离的频率厘摩(cM)来表示。1cM 表示每次减数分裂的重组频率为 1%。cM 值越高表明两点之间遗传距离越远，cM 值越低表示两点间遗传距离越近。

3、物理图谱：表示的是基因或标记间的物理距离，距离的单位为长度单位，如 μm 或者碱基对数（bp 或 kp），物理图谱是基于直接测量的 DNA 结构。

4、RILs（重组自交系）群体：是杂种后代经过多代自交而产生的一种作图群体，通常从 F_2 代开始，采用单粒传的方法来建立。由于自交的作用是使基因型纯合化，因此，RIL 群体中每个株系都是纯合的，因而 RI 群体是一种可以长期使用的永久性分离群体。

5、DH（双单倍体）群体：高等植物的单倍体经过染色体加倍形成的二倍体，DH 群体产生的途径很多，亦因物种不同而异，最常见的方法是通过花药培养，即取 F_1 植株的花药进行离体培养，诱导产生单倍体植株，然后对染色体进行加倍产生 DH 植株。DH 植株是纯合的，自交后即产生纯系，因此 DH 群体可以稳定繁殖，长期使用，是一种永久性群体。

6、BC_1 群体：又称回交一代，也是一种常用的作图群体，为 A 与 B 的杂交 F_1 代与 A 或者 B 回交产生的子代，直接反映了 F1 代配子的分离比例。

7、F_2 群体：为 A 与 B 的杂交 F_1 代经自交产生的子代，是一种暂时性分离群体。

8、分离群体

第 1 类：暂时性分离群体：如 F2、F3、F4、BC、三交群体等，这类群体中分离单位是个体，一经自交或近交其遗传组成就会发生变化，无法永久使用。

第 2 类：永久性分离群体：如 RILs、DH 群体等，这类群体中分离单位是株系，不同株系之间存在基因型的差异，而株系内个体间的基因型是相同且纯合的，是自交不分离的。这类群体可通过自交或近交繁殖后代，而不会改变群体的遗传组成，可以永久使用。

9、连锁群：标记组成的序列，染色体是碱基组成的序列，连锁群上的标记只是存在多态性的碱基位点，即 SNP，所以来自于同一条染色体的标记一定构建到同一个连锁群上。连锁群的数目和染色体的数目等同，表明图谱构建的质量比较高。

染色体和连锁群示意图

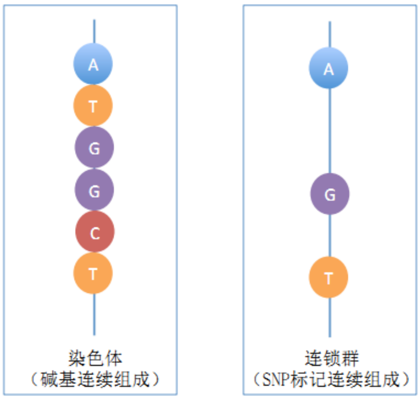

10、偏分离：指 DNA 标记在群体中的分离偏离孟德尔比例；遗传图谱连锁分析的前提假设是各基因座位等位基因分离符合孟德尔分离定律。若存在偏分离标记，将影响交换重组推断的准确性。

11、重组率：指重组型个体占总个体数的百分率。

全基因组关联分析基础概念

1、全基因组关联分析：又称为 Genome-wide association study（GWAS）是一种对全基因组范围内的常见遗传变异（单核苷酸多态性和拷贝数）基因总体关联分析的方法，在全基因组范围内进行整体研究，能够一次性对目标性状进行轮廓性概览，适用于复杂性状的研究。

2、群体分层: 指群体内存在亚群的现象，亚群内部个体间的相互关系大于整个群体内部个体间的平均亲缘关系。由于不同的亚群间，某些稳定的等位基因频率不同，当将两个亚群混合进行关联分析时，就会导致假阳性结果的产生。所以进行关联分析之前，一定要先进行群体分层分析。群体分层分析有群体进化树分析和主成分分析，两者的结果可以进行相互验证。

3、单倍型（haplotype) ：是单倍体基因型的简称，在遗传学上是指在同一染色体上进行共同遗传的多个基因座上等位基因的组合;通俗的说法就是若干个决定同一性状的紧密连锁的基因构成的基因型。

群体进化基础概念

1、群体主成分分析（PCA）：基于个体基因组 SNP 差异程度，通过聚类分析，可将不同性状特征或地理分布的个体按照特征向量值分成不同的亚群。

2、群体遗传结构分析：遗传变异在物种或群体中的一种非随机分布。按照地理分布或其他标准可将一个群体分为若干亚群，处于同一亚群内的不同个体亲缘关系较高，而亚群与亚群之间则亲缘关系稍远。群体结构分析有助于理解进化过程，并且可以通过基因型和表型的关联研究确定个体所属的亚群。

3、群体进化树分析：描述群体间进化顺序的分支图或树，用来表示群体间的进化关系。根据群体的物理或遗传学特征等方面的共同点或差异可以推断出它们的亲缘关系远近。

4、连锁不平衡分析：一般来说，在连锁不平衡分析中，驯化种由于受到了正选择的作用，LD 值就会较野生型偏大，这是因为在一段 DNA 序列中，位点与位点之间存在着连锁的关系。不同位点间的连锁构成了“单倍体型”，随着重组的积累，特定的单倍体型会被削弱而逐渐消失。由于重组率与连锁距离有关，所以连锁不平衡范围会逐渐缩短。对于新产生的一个单倍体型，由于重组来不及破坏位点之间的连锁，所以它们之间连锁不平衡的距离往往比较远。在中性条件下，如果某个单倍体型是较新产生的，那么它的频率往往较低，而频率较高的单倍体型，需要经历很长一段时间才可能因为受到随机漂变的影响达到较高的频率。如果群体经历了正向选择，那么与有利位点连锁的周围位点会由于搭载效应而导致频率很快提升，所以包含有利位点的单倍体型一方面有着较高的频率，另一方面由于经历的时间不长，因此也有这较长的 LD 影响范围。这种特征为检测是否发生了正向选择提供了一个有效的突破点。

5、群体选择消除分析：由于某一点受到强选择后，其周围位点的多态性因受该位点牵连而发生多态性降低的现象。突变后的位点因对物种在特定的情况下有利或者受到了人为的选择，那么该突变位点在群体中的频率必然提高。其附近和它处在同一个单体型或者block的其他多态性位点同样跟着受到选择，频率提高，从而降低了整个周围区域的多态性。常用分析方法有θ_π通过群体遗传信息差异（SNP）反应群体基因组碱基多样性；

Tajima′D 反应群体受到正向选择的强度；

Fst 反应两个群体的差异。0<=F*st*<=1, F*st* 为 1 表示亚种群间存在明显的种群分化。

6、有效群体大小分析：指与实际群体有相同基因频率方差或相同杂合度衰减率的理想群体含量，通常小于绝对的群体大小。它决定了群体平均近交系数增量的大小，反映了群体遗传结构中基因的平均纯合速度，是群体遗传学研究的一个重要内容。有效群体大小的研究有利于我们更清楚的了解种群进化历史和复杂性状的遗传机制，也在畜禽育种中有重要的指导作用。

Hi-C 基础概念

1、Hi－C 技术：是高通量染色体构象捕获技术（High-throughput chromosome conformation capture），利用高通量测序技术，结合生物信息学分析方法，研究全基因组范围内整个染色质 DNA 在空间位置上的关系，构建染色体跨度单体型，同时捕获不同基因座位上之间的空间交互信息，获得高分辨率的染色质三维结构信息，同时开发调控基因的 DNA 元件。

2、互作图谱（heatmap）：是互作频率矩阵可视化图形，横纵坐标，既可以表示同一条染色体的位置坐标又可以表示不同染色体的位置编号，图 1 分别表示全部染色体间和 14 号染色体内部的互作图谱。

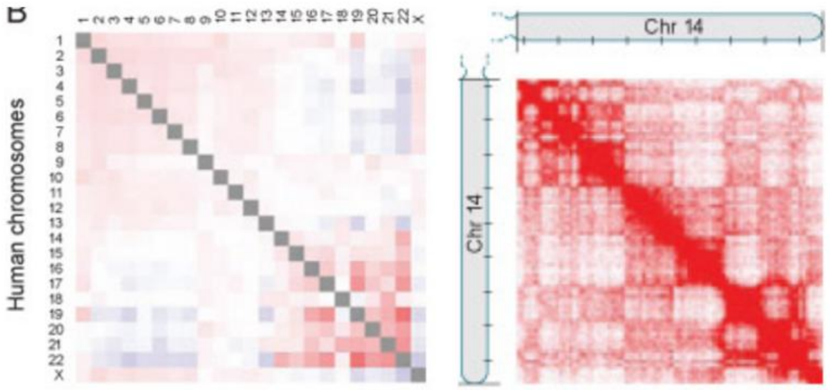

图 1：全部染色体间和单条染色体内的互作图谱

3、TAD（topologically associating domains）：拓扑关联域是指具有“特殊功能”的相互作用“方块”，区域内部的互作频率显著高于毗邻的两个区域的互作频率，一般是互作图谱对角线以下所对应的区域，如图 2 所示：

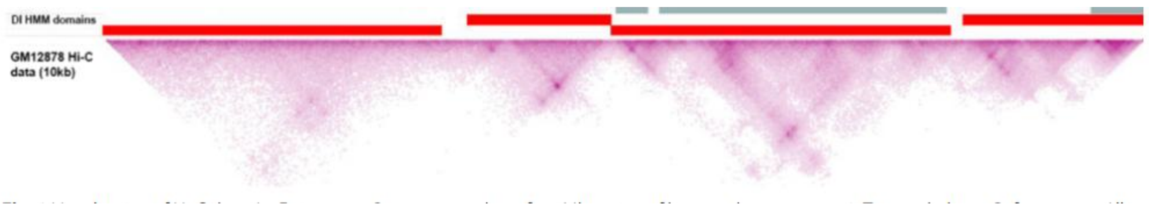

图 2：互作图谱上的 TAD

4、染色体疆域：是指生物学意义上的染色体所具有的特定的“边界”，而该“边界”有助于区分不同的染色体，基于染色体疆域以及染色体内的相互作用要强于染色体间的相互作用，可以借助 Hi-C 所得到的热图的互作频率的强弱，来验证染色体的疆域。
