# Supplementary material for: Gene mapping and development of molecular markers for thousand-grain weight in rye based on bulked segregant analysis
Source: PeerJ. 2026 Feb 12;14:e20811. doi: 10.7717/peerj.20811 (PMC12906707; doi:10.7717/peerj.20811)
Supplement: Supplemental Information 1 [file peerj-14-20811-s001.zip › Supplementary 1/src/pdf/Gtf_Gff.pdf]

动植物重测序 Gff 和 Gff 注释文件说明

诺禾致源动植物重测序业务线

Gff（General Feature Format）是 Sanger 研究定义的一种简单的、方便的对于 DNA、RNA 以及蛋白质序列的特征进行描述的一种数据格式。Gff 是存文本文件，由 tab 键隔开的 9 列组成，Gff3 可以经过 Gff 转换而来，以下是各列说明。

第一列：“seqid”：序列的编号，编号的有效字[a-zA-Z0-9.:^*$@!+_?-|]；

第二列：“source”：注释信息的来源，比如”Genescan”、”Genbank” 等，可以为空，为空用”.”点号代替；

第三列：“type”：注释信息的类型，标准类型为"CDS", "start_codon", "stop_codon", and "exon"；

第四列：“start”：开始位置，注意计数是从 1 开始的；

第五列：“end”：结束位置，结束位置不能大于序列的长度

第六列：“score”：得分（0-1000 之间），数字，是注释信息可能性的说明，可以是序列相似性比对时的 E-values 值或者基因预测是的 P-values 值。”.”表示为空。

第七列：“strand”：序列的方向， +表示正义链, -反义链 , ? 表示未知.

第八列：“phase”：仅对注释类型为 “CDS”有效，表示起始编码的位置，有效值为 0、1、2。

第九列：“attributes”： 以多个键值对组成的注释信息描述，键与值之间用”=“，不同的键值用”;“隔开，一个键可以有多个值，不同值用”,“分割。

预先定义的键包括：ID 注释信息的编号，在一个 GFF 文件中必须唯一；Name 注释信息的名称，可以重复；Alias 别名 Parent Indicates 该注释所属的注释，值为注释信息的编号，比如外显子所属的转录组编号，转录组所属的基因的编号等。

Gtf（Gene Transfer Format）是对 Gff 文件的优化，前 8 列和 Gff 文件相同，第九列不同。

Gtf 的第九列必须以 gene_id value；transcript_id value 开头。

备注：

（1）Gff 和 Gtf 的模板请见表 1 和表 2；

表 1：gff 模板

seqid	source	type	start	end	score	strand	phase	attributes
1	protein_coding	mRNA	27413971	27415335	.	+	.	ID=AT1G72850.1;
1	protein_coding	CDS	27413971	27414389	.	+	0	Parent=AT1G72850.1;
1	protein_coding	CDS	27414486	27415335	.	+	1	Parent=AT1G72850.1;
1	protein_coding	mRNA	20922543	20925619	.	-	.	ID=AT1G55940.1;
1	protein_coding	CDS	20922543	20922901	.	-	2	Parent=AT1G55940.1;
1	protein_coding	CDS	20922989	20923067	.	-	0	Parent=AT1G55940.1;
1	protein_coding	CDS	20923154	20923246	.	-	0	Parent=AT1G55940.1;
1	protein_coding	CDS	20923326	20923634	.	-	0	Parent=AT1G55940.1;
1	protein_coding	CDS	20923669	20923821	.	-	0	Parent=AT1G55940.1;
1	protein_coding	CDS	20923926	20924256	.	-	1	Parent=AT1G55940.1;

表 2：gtf 模板

seqid	source	type	start	end	score	strand	phase	attributes
1	Protein_coding	exon	3631	3913	.	+	.	gene_id "AT1G01010"; transcript_id "AT1G01010.1"; exon_number "1"; gene_name "ANAC001"; transcript_name "ANAC001-201"; seqedit "false";
1	protein_coding	CDS	3760	3913	.	+	0	gene_id "AT1G01010"; transcript_id "AT1G01010.1"; exon_number "1"; gene_name "ANAC001"; transcript_name

								"ANAC001-201"; protein_id "AT1G01010.1";
1	protein_coding	start_codon	3760	3762	.	+	0	gene_id "AT1G01010"; transcript_id "AT1G01010.1"; exon_number "1"; gene_name "ANAC001"; transcript_name "ANAC001-201";
1	protein_coding	exon	3996	4276	.	+	.	gene_id "AT1G01010"; transcript_id "AT1G01010.1"; exon_number "2"; gene_name "ANAC001"; transcript_name "ANAC001-201"; seqedit "false";
1	protein_coding	CDS	3996	4276	.	+	2	gene_id "AT1G01010"; transcript_id "AT1G01010.1"; exon_number "2"; gene_name "ANAC001"; transcript_name "ANAC001-201"; protein_id "AT1G01010.1";
1	protein_coding	exon	4486	4605	.	+	.	gene_id "AT1G01010"; transcript_id "AT1G01010.1"; exon_number "3"; gene_name "ANAC001"; transcript_name "ANAC001-201"; seqedit "false";
1	protein_coding	CDS	4486	4605	.	+	0	gene_id "AT1G01010"; transcript_id "AT1G01010.1"; exon_number "3"; gene_name "ANAC001"; transcript_name "ANAC001-201"; protein_id "AT1G01010.1";

1	protein_coding	exon	4706	5095	.	+	.	gene_id "AT1G01010"; transcript_id "AT1G01010.1"; exon_number "4"; gene_name "ANAC001"; transcript_name "ANAC001-201"; seqedit "false";
1	protein_coding	CDS	4706	5095	.	+	0	gene_id "AT1G01010"; transcript_id "AT1G01010.1"; exon_number "4"; gene_name "ANAC001"; transcript_name "ANAC001-201"; protein_id "AT1G01010.1";
1	protein_coding	exon	5174	5326	.	+	.	gene_id "AT1G01010"; transcript_id "AT1G01010.1"; exon_number "5"; gene_name "ANAC001"; transcript_name "ANAC001-201"; seqedit "false";

（2）参考网址：

http://www.douban.com/note/385690568/

http://wenda.haosou.com/q/1385370455129305
