# Supplementary material for: Gene mapping and development of molecular markers for thousand-grain weight in rye based on bulked segregant analysis
Source: PeerJ. 2026 Feb 12;14:e20811. doi: 10.7717/peerj.20811 (PMC12906707; doi:10.7717/peerj.20811)
Supplement: Supplemental Information 1 [file peerj-14-20811-s001.zip › Supplementary 1/src/pdf/Methods.pdf]

Methods

Sample collection and preparation

DNA quantification and qualification

DNA degradation and contamination was monitored on 1% agarose gels.

DNA purity was checked using the NanoPhotometer® spectrophotometer (IMPLEN, CA, USA).

DNA concentration was measured using Qubit® DNA Assay Kit in Qubit® 2.0 Flurometer (Life Technologies, CA, USA).

Library preparation

A total amount of 1.5μg DNA per sample was used as input material for the DNA sample preparations. Sequencing libraries were generated using Truseq Nano DNA HT Sample preparation Kit (Illumina USA) following manufacturer's recommendations and index codes were added to attribute sequences to each sample. Briefly, the DNA sample was fragmented by sonication to a size of 350bp, then DNA fragments were end polished, A-tailed, and ligated with the full-length adapter for Illumina sequencing with further PCR amplification. At last, PCR products were purified (AMPure XP system) and libraries were analyzed for size distribution by Agilent2100 Bioanalyzer and quantified using real-time PCR.

Illumina sequencing

These libraries constructed above were sequenced by Illumina HiSeq4000 platform and 150bp paired-end reads were generated with insert size around 350bp.

Data analysis

Quality control

To make sure reads reliable and without artificial bias (low quality paired reads, which mainly resulted from base-calling duplicates and adapter contamination) in the following analyses, raw data (raw reads) of fast format was firstly processed through a series of quality control (QC) procedures in-house C scripts. QC standards as the following:

(1) Removing reads with ≥10% unidentified nucleotides (N);

(2) Removing reads with > 50% bases having phred quality < 5;

(3) Removing reads with > 10 nt aligned to the adapter, allowing ≤10% mismatches;

(4) Removing putative PCR duplicates generated by PCR amplification in the library construction process (read 1 and read 2 of two paired-end reads that were completely identical).

Mapping to reference genome

BWA（Burrows-Wheeler Aligner）[1] was used to align the clean reads of each samples against the reference genome (settings: mem -t 4 -k 32 –M -R). Alignment files were converted to BAM files using SAMtools software [2] (settings: –bS –t). In addition, potential PCR duplications were removed using SAMtools command “rmdup”. If multiple read pairs have identical external coordinates, only retain the pair with the highest mapping quality.

SNP/InDel detection and annotation

Variants calling were performed for all samples by using the Unified Genotyper function in GATK [3] software. SNP was used the Variant Filtration parameter in GATK (settings: --filterExpression "QD < 4.0 || FS > 60.0 || MQ < 40.0 ", -G_filter "GQ<20", --cluster WindowSize 4,). InDel was filtered by Variant Filtration parameter (settings: --filter Expression "QD < 4.0 || FS > 200.0 ||Read PosRankSum < -20.0 || Inbreeding Coeff < -0.8 "). ANNOVAR[4], an efficient software tool, was used to annotate SNP or InDel based on the GFF3 files for the reference genome.

SNP/InDel Index

The homozygous SNPs/InDels between two parents were extracted from the vcf files for SNP/InDel. The reads depth information for homozygous SNPs/InDels above in the offspring pools was gained to calculate the SNP/InDel index[5]. We used the genotype of one parent as the reference and to statistic reads number for this parent’s genotype or the others in offspring pool. Then calculate the ratio of the number of different reads in total number, which is the SNP/InDel index of the base sites. We filtered out those points which SNP/InDel index in both pools are less than 0.3. Sliding window methods was used to present the SNP/InDel index of whole genome. The average of all SNP/InDel index in each window was as the SNP/InDel index for this window. Usually we use window size of 1Mb and step size if 10Kb as default settings. The difference of the SNP/InDel index of two pools was calculated as the delta SNP/InDel index.

References

[1].Li H, Durbin R: Fast and accurate short read alignment with Burrows-Wheeler transform. Bioinformatics 2009, 25(14):1754-1760.

[2] Li H, Handsaker B, Wysoker A, et al. The sequence alignment/map format and SAMtools[J]. Bioinformatics, 2009, 25(16): 2078-2079.

[3].McKenna A, Hanna M, Banks E, et al. The Genome Analysis Toolkit: a MapReduce framework for analyzing next-generation DNA sequencing data [J]. Genome research, 2010, 20(9): 1297-1303.

[4].Wang K, Li M, Hakonarson H: ANNOVAR: functional annotation of genetic variants from high-throughput sequencing data. Nucleic acids research 2010, 38(16):e164.

[5].Takagi H, Abe A, Yoshida K, et al. QTL-seq: rapid mapping of quantitative trait loci in rice by whole genome resequencing of DNA from two bulked populations [J]. The Plant Journal, 2013, 74(1): 174-183.
