# Supplementary material for: Gene mapping and development of molecular markers for thousand-grain weight in rye based on bulked segregant analysis
Source: PeerJ. 2026 Feb 12;14:e20811. doi: 10.7717/peerj.20811 (PMC12906707; doi:10.7717/peerj.20811)
Supplement: Supplemental Information 1 [file peerj-14-20811-s001.zip › Supplementary 1/src/pdf/results.pdf]

BSA 性状定位结果文件整理

诺禾致源作物业务线 2018.5

说明：此说明对结果文件的整体进行介绍，每个文件的具体信息可以参照相关文件的README。

主目录：results 文件夹

包含 7 个子文件夹，分别为：

1 Original Data

2 Quality Control

3 Mapping

4 SNP_VarDetect

5 indel_VarDetect

6 SNP_index_indel_index_analysis：重要，第二个看

7 Trait_loci：重要，第一个先看这个文件夹的内容

详细说明见下表：

<table>
<tr><th colspan="4">Results</th><th>结果文件说明</th><th>对应结题报告</th></tr>
<tr><td rowspan="14">results</td><td rowspan="2">1.OriginalData
测序数据格式说明</td><td colspan="2">1.OriginalData.README.txt</td><td>说明文档</td><td rowspan="2">1</td></tr>
<tr><td colspan="2">example.fq.txt</td><td>数据示例</td></tr>
<tr><td rowspan="8">2.QualityControl
测序数据质控结果</td><td rowspan="2">2.1.QualityDistribution
测序质量分布结果</td><td>2.1.QualityDistribution.README.txt</td><td>说明文档</td><td rowspan="2">2.1</td></tr>
<tr><td>*.QM.png</td><td>测序质量分布图</td></tr>
<tr><td rowspan="2">2.2.ErrorRate
测序错误率分布结果</td><td>2.2.ErrorRate.README.txt</td><td>说明文档</td><td rowspan="2">2.2</td></tr>
<tr><td>*.Error.png</td><td>错误率分布图</td></tr>
<tr><td rowspan="2">2.3.ReadsClassification
测序数据过滤分布结果</td><td>2.3.ReadsClassification.README.txt</td><td>说明文档</td><td rowspan="2">2.3</td></tr>
<tr><td>*.raw_reads_classification.png</td><td>原始数据组成分布图</td></tr>
<tr><td rowspan="2">2.4.CleanData_Qcsummary 测序数据质控统计结果</td><td>2.4.CleanData_QCsummary.README.txt</td><td>说明文档</td><td rowspan="2">2.4</td></tr>
<tr><td>CleanData_QCsummary.xls</td><td>质控数据汇总表</td></tr>
<tr><td rowspan="4">3.Mapping
样本与参考基因组的比对统计结果</td><td rowspan="4">3.1.Reference 参考基因组基本信息统计</td><td>3.1.Reference.README.txt</td><td>说明文档</td><td rowspan="2">3.1</td></tr>
<tr><td>genome_stat.xls</td><td>参考基因组统计结果</td></tr>
<tr><td>ref.fa 和 ref.fa.fai</td><td>参考基因组序列</td><td>——</td></tr>
<tr><td>gff3</td><td>参考基因组对应的注释文件</td><td>——</td></tr>
</table>

<table>
<tr><td rowspan="32"></td><td rowspan="3"></td><td rowspan="3">3.2.MapStat
测序深度及覆盖度统计</td><td>3.2.MapStat.README.txt</td><td>说明文档</td><td rowspan="3">3.2</td></tr>
<tr><td>*.Sequencing-depth.png/pdf</td><td>测序深度统计图</td></tr>
<tr><td>Allsample_alninfo.xls</td><td>测序深度统计表</td></tr>
<tr><td rowspan="5">4.SNP_VarDetect
SNP 变异位点信息、统计和注释结果</td><td colspan="2">4.SNP_VarDetect.README.txt</td><td>说明文档</td><td></td></tr>
<tr><td colspan="2">SNP.vcf</td><td>SNP 变异 vcf 文件</td><td rowspan="4">4.1</td></tr>
<tr><td colspan="2">snp.avinput.exonic_variant_function</td><td>SNP 外显子注释结果</td></tr>
<tr><td colspan="2">snp.avinput.variant_function</td><td>SNP 全基因组注释结果</td></tr>
<tr><td colspan="2">SNP_Annotation_Statistics.xls</td><td>SNP 注释统计</td></tr>
<tr><td rowspan="5">5. indel_VarDetect
Indel 变异位点信息、统计和注释结果</td><td colspan="2">5.indel_VarDetect.README.txt</td><td>说明文档</td><td></td></tr>
<tr><td colspan="2">Indel.vcf</td><td>Indel 变异 vcf 文件</td><td></td></tr>
<tr><td colspan="2">indel.avinput.exonic_variant_function</td><td>Indel 外显子注释结果</td><td></td></tr>
<tr><td colspan="2">indel.avinput.variant_function</td><td>Indel 全基因组注释结果</td><td></td></tr>
<tr><td colspan="2">Indel_Annotation_Statistics.xls</td><td>Indel 注释统计</td><td></td></tr>
<tr><td rowspan="12">6.SNP_index_indel_index_analysis
变异位点的 index 分析</td><td colspan="2">6.SNPindex_analysis.README.txt</td><td>说明文档</td><td></td></tr>
<tr><td rowspan="9">6.1.SNPindex_analysis
SNP 的 index 分析结果</td><td>S1.SNP_index.manhattan.pdf/png</td><td>子代池 1 的 indexmanhattan 图</td><td rowspan="2">5.2</td></tr>
<tr><td>S2.SNP_index.manhattan.pdf/png</td><td>子代池 2 的 indexmanhattan 图</td></tr>
<tr><td>Delta_SNPindex.manhattan.pdf/png</td><td>Deltaindex 的 manhattan 图</td><td>5.3</td></tr>
<tr><td>S1.SNP_index.pdf/png</td><td>子代池 1 的 index 分布图</td><td rowspan="2">5.2</td></tr>
<tr><td>S2.SNP_index.pdf/png</td><td>子代池 2 的 index 分布图</td></tr>
<tr><td>Delta_SNPindex.png/pdf</td><td>delta index 分布图</td><td>5.3</td></tr>
<tr><td>candidateSNPindex.xls</td><td>候选 SNP 位点</td><td>5.5</td></tr>
<tr><td>Filter_SNP_index.xls</td><td>过滤后的 SNPindex 列表（1 个子代池的释放）</td><td>5.1</td></tr>
<tr><td>Delta_SNPindex.xls</td><td>SNPindex 差值列表（2 个子代池的释放）</td><td>——</td></tr>
<tr><td>6.2.indel_index_analysis
Indel 的 index 分析结果</td><td>同 6.1</td><td>indel 的 index 计算相关结果文件</td><td>6</td></tr>
<tr><td>6.3.all_index_analysis
SNP 和 Indel 合并的 index 分析结果</td><td>同 6.1</td><td>SNP 与 indel 的 index 合并计算的结果文件</td><td>7</td></tr>
<tr><td rowspan="7">7.Trait_loci</td><td colspan="2">7.Trait_loci.README.txt</td><td>说明文档</td><td></td></tr>
<tr><td colspan="2">candidateSNPindex.avinput_variant_function</td><td>候选 SNP 位点注释</td><td>——</td></tr>
<tr><td colspan="2">candidateSNPindex.exonic_variant_function</td><td>候选 SNP 位点注释</td><td>——</td></tr>
<tr><td colspan="2">candidate_SNP_gene.xls</td><td>候选基因列表</td><td rowspan="2">5.5</td></tr>
<tr><td colspan="2">candidateSNP_Annotation_Statistics.xls</td><td>候选 SNP 位点注释统计</td></tr>
<tr><td colspan="2">candidateAllindex_SNP*</td><td>allindex 分析的候选 SNP 位点注释及候选基因</td><td rowspan="2">7.4</td></tr>
<tr><td colspan="2">candidateAllindex_InDel*</td><td>allindex 分析的候选 indel 位点注释及候选基因</td></tr>
</table>
