# Supplementary material for: Gene mapping and development of molecular markers for thousand-grain weight in rye based on bulked segregant analysis
Source: PeerJ. 2026 Feb 12;14:e20811. doi: 10.7717/peerj.20811 (PMC12906707; doi:10.7717/peerj.20811)
Supplement: Supplemental Information 1 [file peerj-14-20811-s001.zip › Supplementary 1/src/pdf/Software.Resequencing.pdf]

Analysis	Software	Version	Remarks
比对	BWA	0.7.10	和参考基因组比对
SAM和BAM文件转化	samtools	0.1.19	
SNP calling	GATK	3.3-0	群体SNPcalling
SNP注释	ANNOVAR	2013Aug23	SNP根据基因位置注释
