# Supplementary material for: Gene mapping and development of molecular markers for thousand-grain weight in rye based on bulked segregant analysis
Source: PeerJ. 2026 Feb 12;14:e20811. doi: 10.7717/peerj.20811 (PMC12906707; doi:10.7717/peerj.20811)
Supplement: Supplemental Information 1 [file peerj-14-20811-s001.zip › Supplementary 1/src/result_tree.html]

Directory Tree


# Directory Tree

../../results
  
 ├── 1.OriginalData
  
 ├── 2.QualityControl
  
 │   ├── 2.1.QualityDistribution  
 │   ├── 2.2.ErrorRate  
 │   ├── 2.3.ReadsClassification  
 │   └── 2.4.CleanData\_QCsummary  
 ├── 3.Mapping
  
 │   └── 3.2.MapStat  
 ├── 4.SNP\_VarDetect
  
 ├── 5.indel\_VarDetect
  
 ├── 6.SNP\_index\_indel\_index\_analysis
  
 │   ├── 6.1.SNPindex\_analysis  
 │   ├── 6.2.indel\_index\_analysis  
 │   └── 6.3.all\_index\_analysis  
 └── 7.Trait\_loci

15 directories

---

tree v1.5.3 © 1996 - 2009 by Steve Baker and Thomas Moore   
HTML output hacked and copyleft © 1998 by Francesc Rocher   
Charsets / OS/2 support © 2001 by Kyosuke Tokoro
