# Supplementary material for: Gene mapping and development of molecular markers for thousand-grain weight in rye based on bulked segregant analysis
Source: PeerJ. 2026 Feb 12;14:e20811. doi: 10.7717/peerj.20811 (PMC12906707; doi:10.7717/peerj.20811)
Supplement: Supplemental Information 1 [file peerj-14-20811-s001.zip › Supplementary 1/X101SC21123089-Z01-J001_Report.html]

BSA性状定位项目结题报告 


- 建库测序流程
  - DNA样品检测
  - 文库构建及库检
  - 上机测序
- 信息分析流程
- 结果展示及说明
  - 原始序列数据
  - 测序数据质量评估
  - 比对统计
  - SNP和InDel检测及注释
  - 子代SNP频率差异分析
  - 子代InDel频率差异分析
  - 子代SNP及InDel频率差异分析
  - 目标性状区域定位
  - 附录
    - 软件列表
    - Methods英文版
    - 动植物重测序名词解释
    - 结果文件使用说明
    - 备注
  - 参考文献


# 诺禾致源BSA性状定位项目结题报告

问题反馈邮箱:op-ccx@novogene.com

### 项目编号:X101SC21123089-Z01-J001

### 项目名称:河北科技师范学院黑麦BSA测序分析技术服务（委托）合同

### 报告时间:2022-03-03

### 报告编号:X101SC21123089-Z01-J001-B1-2

### 报告查看说明

    诺禾致源BSA项目报告为网页版报告；右上角有悬浮窗口和下拉菜单，点击可跳转到结题报告的相应部分，点击最右侧圆形按钮将隐藏悬浮窗，点击网页刷新后重新显示；点击下方目录中各级标题也可进行跳转；点击报告中novogene logo可跳转至报告开头

### 项目结果概述

    1、建库测序：对项目样本DNA进行质量检测，构建350bp的WGS文库，库检合格后进行Illumina PE150测序；

    2、测序结果：测序共产生 Raw data 438.1G，过滤后的 Clean data 428.222G，测序质量高（Q20 ≥ 96.88%、Q30 ≥ 91.82%）[交付标准：Q20 ≥ 85%,Q30 ≥ 80%]，GC含量在45.66%~46.1%之间；

    3、比对结果：采用BWA软件比对，所有样本的比对率在0.0%~0.0%之间， 对参考基因组（排除N区）的平均覆盖深度在8.3X~15.31X之间， 1X覆盖度（至少有一个碱基的覆盖）在78.73%以上。比对结果正常；

    4、SNP检测：采用GATK3.8软件的UnifiedGenotyper模块进行多个样本SNP和InDel的检测，使用VariantFiltration进行过滤，得到群体vcf文件，采用ANNOVAR软件对变异位点进行注释，给出变异位点所在基因与变异类型。软件及过滤参数详见“4 SNP和InDel的检测”；

    5、候选区间：筛选亲本纯合差异的变异位点，以其中一个亲本（默认P1）作为参考，分别计算两个子代池的index值；然后划窗口计算每个窗口的index平均值，定位候选区间，95%和99%阈值条件下的候选区间列表请见：SNP\_can95.bed.xls ; SNP\_can99.bed.xls;

    6、候选基因：根据群体表型特征，子代池是否为纯合，子代群体性状分离比等设定index阈值为0.2和0.8，挑选出3,833个SNP候选位点和652个InDel候选位点，根据ANNOVAR的注释结果,优先挑选upstream, stop loss, stop gain, 非同义突变, 可变剪接等重要突变位点所在的基因作为候选基因。候选基因列表请见：candidate.snp\_gene.xls ; candidate.InDel\_gene.xls。

    7、下图为报告结果文件展示图。

北京诺禾致源科技股份有限公司


---

目录 - 一、建库测序流程

- 1 DNA样品检测
- 2 文库构建及库检
- 3 上机测序

- 二、信息分析流程
- 三、结果展示及说明

- 1 原始序列数据
- 2 测序数据质量评估
- 3 比对统计
- 4 SNP和InDel检测及注释
- 5 子代SNP频率差异分析
- 6 子代InDel频率差异分析
- 7 子代SNP及InDel频率差异分析
- 7.4 基于SNP和InDel标记的目标性状区域定位

- 四、附录

- 1 软件列表
- 2 Methods英文版
- 3 动植物重测序名词解释
- 4 结果文件使用说明
- 5 备注

- 五、参考文献


北京诺禾致源科技股份有限公司


---

  

## 一、 建库测序流程

从DNA样品到最终数据获得，样品检测、建库、测序每一个环节都会对数据质量和数量产生影响，而数据质量又会直接影响后续信息分析的结果。为了从源头上保证测序数据的准确性、可靠性，诺禾致源对样品检测、建库、测序每一个生产步骤都严格把控，从根本上确保了高质量数据的产出。流程图如下：

图1.1 建库测序流程图

### 1 DNA样品检测

诺禾致源对DNA样品的检测主要包括3种方法：  

(1) 琼脂糖凝胶电泳分析DNA的纯度和完整性；  
(2) Nanodrop检测DNA的纯度（OD260/280比值）；  
(3) Qubit对DNA浓度进行精确定量；

### 2 文库构建及库检

检验合格的DNA样品通过Covaris破碎机随机打断成长度为350bp的片段。采用TruSeq Library Construction Kit 进行建库，严格使用说明书推荐的试剂和耗材。DNA片段经末端修复、加ployA尾、加测序接头、纯化、PCR扩增等步骤完成整个文库制备。构建好的文库通过illumina HiSeq^TM^ PE150进行测序（图1.2）。

图1.2 建库测序流程

文库构建完成后，先使用Qubit2.0进行初步定量，稀释文库至1ng/μl，随后使用Agilent 2100对文库的insert size进行检测，insert size符合预期后，使用Q-PCR方法对文库的有效浓度进行准确定量（文库有效浓度>2nM），以保证文库质量。

### 3 上机测序

库检合格后，把不同文库按照有效浓度及目标下机数据量的需求pooling后进行Illumina HiSeq ^TM^ PE150测序。

北京诺禾致源科技股份有限公司


---

  

## 二、生物信息分析流程

信息分析的主要步骤如下：  

    （1） 对下机得到的Raw data进行质控得到Clean data；  

    （2） 将Clean data比对到参考基因组上；

    （3） 根据比对结果，采用GATK3.8进行SNP、InDel的检测及注释。

    （4） 子代变异频率差异分析；

    （5） 性状定位；

    （6） 候选位点注释：通过对候选位点注释得到候选基因；

北京诺禾致源科技股份有限公司


---

  

## 三、 结果展示及说明

### 1　原始序列数据

高通量测序(如Illumina HiSeq^TM^/Miseq^TM^)得到的原始图像数据文件经CASAVA碱基识别(Base Calling)分析转化为原始测序序列（Sequenced Reads），称之为 Raw Data或Raw Reads，结果以 FASTQ (简称为fq)文件格式存储，其中包含测序序列（reads）的序列信息以及其对应的测序质量信息。

FASTQ格式文件中每个read由四行描述，如下：

@HWI-ST1276:71:C1162ACXX:1:1101:1208:2458 1:N:0:CGATGT  
NAAGAACACGTTCGGTCACCTCAGCACACTTGTGAATGTCATGGGATCCAT  
+  
#55???BBBBB?BA@DEEFFCFFHHFFCFFHHHHHHHFAE0ECFFD/AEHH

其中第一行以“@”开头，随后为Illumina 测序标识别符(Sequence Identifiers)和描述文字(选择性部分)；  
第二行是碱基序列；  
第三行以“+”开头，随后为Illumina 测序标识别符(选择性部分)；  
第四行是对应碱基的测序质量，该行中每个字符对应的 ASCII 值减去 33，即为对应第二行碱基的测序质量值。

Illumina测序标识符详细信息如下表：

|  |  |
| --- | --- |
| HWI-ST1276 | Instrument – unique identifier of the sequencer |
| 71 | Run number – Run number on instrument |
| C1162ACXX | FlowCell ID – ID of flowcell |
| 1 | LaneNumber – positive integer |
| 1101 | TileNumber – positive integer |
| 1208 | X – x coordinate of the spot. Integer which can be negative |
| 2458 | Y – y coordinate of the spot. Integer which can be negative |
| 1 | ReadNumber - 1 for single reads; 1 or 2 for paired ends |
| N | Whether it is filtered - NB：Y if the read is filtered out, not in the delivered fastq file, N otherwise |
| 0 | Control number - 0 when none of the control bits are on, otherwise it is an even number |
| CGATGT | Illumina index sequences |

北京诺禾致源科技股份有限公司


---

  

### 2　测序数据质量评估

#### 2.1　测序质量分布检查

如果测序错误率用e表示，Illumina HiSeq^TM^/Miseq^TM^的碱基质量值用Q_phred_表示，则有：Q_phred_=-10log_10_(e)。Illumina Casava 1.8版本碱基识别与Phred分值之间的简明对应关系见下表：

| Phred分值 | 不正确的碱基识别 | 碱基正确识别率 | Q-sorce |
| --- | --- | --- | --- |
| 10 | 1/10 | 90% | Q10 |
| 20 | 1/100 | 99% | Q20 |
| 30 | 1/1000 | 99.9% | Q30 |
| 40 | 1/10000 | 99.99% | Q40 |

图2.1　测序质量分布图

横坐标为reads的碱基位置，纵坐标为单碱基错误率  
前150bp 为双端测序序列的第一端测序 Reads的质量值分布情况，后150bp为另一端测序reads 的质量值分布情况。

北京诺禾致源科技股份有限公司


---

  

#### 2.2　测序错误率分布检查

测序错误率与碱基质量有关，根据测序技术的特点，测序片段前端几个cycles和末端的错误率会偏高。具体见**图 2.2**：

  

图2.2　测序错误率分布图

横坐标为reads的碱基位置，纵坐标为单碱基错误率  
前150bp 为双端测序序列的第一端测序 Reads的错误率分布情况，后150bp为另一端测序reads 的错误率分布情况。

北京诺禾致源科技股份有限公司


---

  

#### 2.3　测序数据过滤

测序得到的原始测序序列（Sequenced Reads）或者 raw reads，里面含有带接头的、低质量的reads。为了保证信息分析质量，必须对raw reads过滤，得到clean reads，后续分析都基于 clean reads。数据处理的步骤如下：

(1) 去除带接头(adapter)的reads pair；  
(2) 当单端测序read中含有的N的含量超过该条read长度比例的10%时，需要去除此对paired reads；  
(3) 当单端测序read中含有的低质量（Q ≤ 5）碱基数超过该条read长度比例的 50% 时，需要去除此对paired reads。

图2.3　原始数据过滤结果

(1) Adapter related：因有接头，过滤掉的 reads数及其占总 raw reads数的比例。
(2) Containing N：因N含量超过 10%，过滤掉的 reads数及其占总raw reads数的比例。  
(3) Low quality：因低质量，过滤掉的reads数及其占总raw reads数的比例。
(4) Clean reads：最终得到的 clean reads 数及其占总 raw reads 数的比例。

北京诺禾致源科技股份有限公司


---

  


#### 2.4　测序数据质量情况汇总

测序数据质量情况汇总详见**表2.4**。

表2.4　测序数据质量情况汇总

| Sample | Raw Base(bp) | Clean Base(bp) | Effective Rate(%) | Error Rate(%) | Q20(%) | Q30(%) | GC Content(%) |
| --- | --- | --- | --- | --- | --- | --- | --- |
| High\_f1 | 150,063,403,500 | 146,738,338,200 | 97.78 | 0.03 | 96.93 | 91.92 | 45.66 |
| 4\_2\_fuben | 71,805,001,200 | 69,990,140,400 | 97.47 | 0.03 | 96.88 | 91.82 | 45.95 |
| Low\_f1 | 143,754,626,400 | 140,384,085,600 | 97.66 | 0.03 | 96.89 | 91.85 | 45.76 |
| 3\_1\_muben | 72,477,255,600 | 71,109,292,200 | 98.11 | 0.03 | 96.98 | 91.97 | 46.10 |

数据质量情况详细内容如下：

(1) Sample: 样本名。  
(2) Raw Base(bp)：原始数据产量，测序序列的个数乘以测序序列的长度，以bp为单位。  
(3) Clean Base(bp)：过滤之后的有效数据量，过滤后测序序列的个数乘以测序序列的长度，以bp为单位。  
(4) Effective Rate(%)：过滤后获得clean data 与raw data的比值。  
(5) Error Rate(%)：碱基错误率。  
(6) Q20、Q30：Phred 数值大于20、30的碱基占总体碱基的百分比。  
(7) GC Content(%)：碱基G和C的数量总和占总的碱基数量的百分比。

  


#### 2.5 测序数据评估小结

本次测序共产生 Raw data 438.1G，过滤后的 Clean data 428.222G，
各样本的 Raw data 在71805.001 M~150063.403 M之间，
测序质量高（Q20 ≥ 96.88%、Q30 ≥ 91.82%），GC含量在45.66%~46.1%之间。

综上，所有样本的数据量足够，测序质量合格，GC分布正常，建库测序成功。

#### 2.6 测序质量评估FAQ

**Q1**：测序错误率在测序片段前端和末端的碱基错误率会偏高，错误率在多少是可以接受的范围？

**答**：在测序片段前端和末端的碱基错误率的偏高是由于测序机器的预热或者试剂的消耗引起的，属于测序技术限制，诺禾致源会对测序数据进行严格的质量把控，一般情况下测序数据的Q20大于85%，Q30大于80%。

**Q2**：诺禾致源质控的标准是什么？

**答**：为保证后续分析的准确性，诺禾致源会严格把控clean data的筛选标准，具体标准如下：  
       **(1)** 去除带接头(adapter)的paired reads；  
       **(2)** 当单端测序read中含有的N的含量超过该条read长度比例的10%时，需要去除此对paired reads；  
       **(3)** 当单端测序read中含有的低质量（Q ≤ 5）碱基数超过该条read长度比例的 50% 时，需要去除此对paired reads。

**相关名词解释**:

**adapter**：接头，用于上机测序。建库时引入的接头序列与测序芯片（flow cell）上固定的接头相互识别。

**index**：测序的标签，用于测定混合样本，通过每个样本添加的不同标签进行数据区分，鉴别测序样品。

**Q20,Q30**：Phred 数值大于20、30的碱基占总体碱基的百分比，其中Phred=-10log10(e),e为错误率。

**raw data/raw reads**：测序下机的原始数据。

**clean data/clean reads**：对原始数据进行过滤后，剔除了低质量数据的剩余数据。后续分析均基于clean data。


北京诺禾致源科技股份有限公司


---

北京诺禾致源科技股份有限公司


---

  

#### 3.2　Reads与参考基因组比对情况统计

样本比对率反映了样本测序数据与参考基因组的相似性，覆盖深度和覆盖度能够直接反应测序数据的均一性及与参考序列的同源性。

表3.2 测序深度及覆盖度统计

| Sample | Mapped reads | Total reads | Mapping rate(%) | Average depth(X) | Coverage at least 1X(%) | Coverage at least 4X(%) |
| --- | --- | --- | --- | --- | --- | --- |
| 3\_1\_muben | 474,061,948 | 467,805,175 | 98.68 | 8.55 | 78.97 | 59.69 |
| 4\_2\_fuben | 466,600,936 | 460,805,114 | 98.76 | 8.30 | 78.73 | 58.55 |
| Low\_f1 | 978,255,588 | 963,739,078 | 98.52 | 14.68 | 87.03 | 74.77 |
| High\_f1 | 935,893,904 | 915,786,528 | 97.85 | 15.31 | 86.81 | 75.24 |

测序深度及覆盖度统计详细内容如下：

(1) Sample: 样本名。  
(2) Mapped reads：比对到reference上的reads条数（包括单端比对和双端比对）。  
(3) Total reads：有效测序数据的reads总条数。  
(4) Mapping rate：比对率，比对到参考基因组上的reads数目除以有效测序数据的reads数目。  
(5) Average depth：平均测序深度，比对到参考基因组的碱基总数除以基因组大小。  
(6) Coverage at least 1X：参考基因组中至少有1个碱基覆盖的位点占基因组的百分比。  
(7) Coverage at least 4X：参考基因组至少有4个碱基覆盖的位点占基因组的百分比。

图3.2 测序深度分布图

横坐标表示测序深度，图中蓝色的线和左侧的纵坐标轴表示对应深度的位点占全基因组的百分比，图中红色的线和右侧纵坐标表示小于或等于该深度的位点占全基因组的百分比。

#### 3.3　比对结果小结

参考基因组大小为7,737,590,180bp，所有样本的比对率在0.0%~0.0%之间，
对参考基因组（排除N区）的平均覆盖深度在8.3X~15.31X之间，
1X覆盖度（至少有一个碱基的覆盖）在78.73%以上。比对结果正常，可用于后续的变异检测及相关分析。

#### 3.4 比对统计FAQ

**Q1**：如果该物种没有参考基因组怎么办？或者如果样品是两个品种的杂交后代，而两个品种都有参考基因组，该选择哪个参考基因组？

**答**：如果该物种没有参考基因组，而近缘物种有参考基因组，可以选择近缘物种的参考基因组进行比对分析。如果两个品种都有参考基因组，则要根据样品的表型和其他数据选择一个与样品差异较小的品种作为参考基因组。已发表参考基因组的物种请参见基因组列表。

**Q2**：造成比对率（mapping rate）较低的原因可能有哪些？

**答**：诺禾致源采用BWA软件进行序列比对，采用mem算法的默认参数进行比对，
比对率低的主要原因可能是参考基因组组装不好，或者所测材料与使用的参考基因组差异比较大，或者有污染所致。

北京诺禾致源科技股份有限公司


---

  

### 4 SNP和InDel检测及注释

SNP(单核苷酸多态性) 主要是指在基因组水平上由单个核苷酸的变异所引起的DNA序列多态性，包括单个碱基的转换、颠换等。采用GATK3.8软件的UnifiedGenotyper模块进行多个样本SNP的检测，使用VariantFiltration进行过滤，过滤参数为 --clusterWindowSize 4，--filterExpression "QD < 4.0 || FS > 60.0 || MQ < 40.0 " ，-G\_filter "GQ<5"。

InDel是指基因组中小片段的插入和缺失序列。采用GATK3.8软件的UnifiedGenotyper模块进行InDel的检测，使用VariantFiltration进行过滤，过滤参数为 --filterExpression "QD < 4.0 || FS > 200.0"。

#### 4.1 SNP和InDel检测及注释结果统计

ANNOVAR是一种高效的软件工具，它能利用最新的信息，对由多个基因组检测出的基因变异进行功能注释。只要给出变异所在的染色体、起始位点、终止位点、参考核苷酸和变异核苷酸，ANNOVAR就能进行Gene-based annotation、Region-based annotations、Filter-based annotation和Other functionalities。鉴于ANNOVAR强大的注释功能和国际的认可性，利用它对SNP检测结果进行注释。

表4.1a SNP检测及注释结果统计

| Category | | Number of SNPs |
| --- | --- | --- |
| Upstream | | 239,683 |
| Exonic | UTR3 | 88,626 |
|  | UTR5 | 45,293 |
|  | UTR5;UTR3 | 231 |
|  | Stop gain | 1,319 |
|  | Stop loss | 210 |
|  | Synonymous | 197,312 |
|  | Non-synonymous | 129,389 |
|  | unknowns | 0 |
| Intronic | | 714,008 |
| Splicing | | 620 |
| Downstream | | 216,991 |
| upstream/downstream | | 9,422 |
| Intergenic | | 32,444,360 |
| Other | | 0 |
| ts | | 21,472,355 |
| tv | | 12,615,109 |
| ts/tv | | 1.702 |
| Total | | 34,087,464 |

SNP检测及注释结果统计详细内容如下：

(1) Category: 类别。  
(2) Upstream：基因上游1 Kb区域。  
(3) Exonic：变异位于外显子区域；  
    UTR5：5‘端非翻译区；UTR3：3‘端非翻译区；UTR5;UTR3：在一个基因的UTR5，同时在另一个基因的UTR3：  
    Stop gain：使基因获得终止密码子的变异；Stop loss：使基因失去终止密码子的变异；  
    synonymous：同义变异：Non-synonymous：非同义突变：  
    unknowns：未注释到的位点。  
(4) Intronic：变异位于内含子区域。  
(5) Splicing：变异位于剪接位点（内含子中靠近外显子/内含子边界的2bp）。  
(6) Downstream：基因下游1 Kb区域。  
(7) Upstream/Downstream: 基因上游1 Kb区域，同时也在另一基因的下游1 Kb区域。  
(8) Intergenic：变异位于基因间区。  
(9) Other：其他类别。  
(10) ts：transitions，转换。  
(11) tv：transversions，颠换。  
(12) ts/tv：转换与颠换的比率。  
(13) Total：SNP位点总数。

表4.1b InDel检测及注释结果统计

| Category | | Number of Indels |
| --- | --- | --- |
| Upstream | | 36,690 |
| Exonic | UTR3 | 17,183 |
|  | UTR5 | 10,938 |
|  | UTR5;UTR3 | 70 |
|  | Stop gain | 139 |
|  | Stop loss | 21 |
|  | Frameshift deletion | 1,793 |
|  | Frameshift insertion | 2,257 |
|  | Non-frameshift deletion | 3,603 |
|  | Non-frameshift insertion | 3,366 |
|  | unknowns | 0 |
| Intronic | | 93,689 |
| Splicing | | 363 |
| Downstream | | 31,490 |
| upstream/downstream | | 1,862 |
| Intergenic | | 2,210,781 |
| Other | | 78 |
| Insertion | | 1,308,550 |
| Deletion | | 1,105,773 |
| Total | | 2,414,323 |

候选InDel位点注释情况详细内容如下：

(1) Category: 类别。  
(2) Upstream：基因上游1 Kb区域。  
(3) Exonic：变异位于外显子区域；  
    UTR5：5‘端非翻译区；UTR3：3‘端非翻译区；UTR5;UTR3；在一个基因的UTR5，同时在另一个基因的UTR3；  
    Stop gain：使基因获得终止密码子的变异；Stop loss：使基因失去终止密码子的变异；  
    Frameshift deletion：移码缺失；Frameshift insertion：移码插入；  
    Non-frameshift deletion：非移码缺失；Non-frameshift insertion：非移码插入；  
    unknowns：未注释到的位点。  
(4) Intronic：变异位于内含子区域。  
(5) Splicing：变异位于剪接位点（内含子中靠近外显子/内含子边界的2bp）。  
(6) Downstream：基因下游1 Kb区域。  
(7) Upstream/Downstream: 基因上游1 Kb区域，同时也在另一基因的下游1 Kb区域。  
(8) Intergenic：变异位于基因间区。  
(9) Other：其他类别。  
(10) Insertion：插入。  
(11) Deletion：缺失。  
(12) Total：InDel位点总数。

#### 4.2 SNP和InDel检测及注释FAQ

**Q1**：对SNP、InDel进行注释使用的基因组注释文件是什么格式？

**答**：诺禾致源利用ANNOVAR进行注释，对检测到的SNP、InDel进行注释，主要利用基因结构注释文件（gtf/gff/gff3/bed等格式，推荐gtf，gff）。gff和gtf文件的格式说明，同时注释文件需要和参考基因组配套，即参考基因组和gff文件中的染色体名称一致。（pdf说明请见：PDF），

**Q2**：SNP、InDel的纯合和杂合的定义？

**答**：SNP、InDel的纯合和杂合是利用GATK软件根据测序深度、碱基质量值、比对质量值和基因型质量值等综合判断出来的。简单来说，纯合SNP/InDel可以认为该位点测到的所有reads只是一种碱基类型；杂合SNP/InDel为二种或二种以上的碱基类型，不排除特殊位置。


北京诺禾致源科技股份有限公司


---

  

### 5 子代SNP频率差异分析

#### 5.1 子代SNP频率计算

通过计算SNP-index值来进行性状定位，选择亲本3\_1\_muben作为参考亲本，分别计算两个子代的SNP-index（即SNP的频率）。SNP-index计算方法如图5.1所示。其中，完全与其相同的:SNP-index为0；完全与其不同的SNP-index为1。为了去除背景噪音、减少测序错误比对错误等造成的影响和提高定位的效率，对SNP和SNP-index进行筛选和过滤，筛选和过滤标准如下：

(1) 对于两个亲本的群体，筛选两个亲本间纯合差异的标记；共挑选出2,513,966个多态性标记；

(2) 子代中 SNP-index 小于0.3，或深度小于7的位点，过滤掉;

(3) 一个子代SNPindex缺失的位点，过滤掉;

经过以上筛选，得到过滤后的1,485,248个多态性标记位点，部分位点SNPindex信息见表5.1,亲本加两个子代池index的计算方式见图5.2。

图5.1 SNP-index 计算方法

表5.1 部分位点SNPindex信息

| CHROM | POS | REF | ALT | Genotype\_3\_1\_muben | Depth\_3\_1\_muben | Ref\_depth\_Low\_f1 | Alt\_depth\_Low\_f1 | Depth\_Low\_f1 | SNPindex\_Low\_f1 | Ref\_depth\_High\_f1 | Alt\_depth\_High\_f1 | Depth\_High\_f1 | SNPindex\_High\_f1 |
| --- | --- | --- | --- | --- | --- | --- | --- | --- | --- | --- | --- | --- | --- |
| GWHASIY00000001 | 358165 | T | A | T | 9 | 7 | 4 | 11 | 0.36 | 7 | 7 | 14 | 0.50 |
| GWHASIY00000001 | 358253 | G | T | G | 6 | 7 | 3 | 10 | 0.30 | 8 | 5 | 13 | 0.38 |
| GWHASIY00000001 | 359040 | T | G | T | 4 | 3 | 4 | 7 | 0.57 | 5 | 2 | 7 | 0.29 |
| GWHASIY00000001 | 371412 | A | G | A | 6 | 12 | 3 | 15 | 0.20 | 6 | 8 | 14 | 0.57 |
| GWHASIY00000001 | 386723 | G | T | G | 7 | 5 | 3 | 8 | 0.38 | 3 | 4 | 7 | 0.57 |

部分位点SNPindex信息详细内容如下：

(1) CHROM：染色体名。  
(2) POS：SNP位点。  
(3) REF：该位点参考基因组碱基型。  
(4) ALT：该位点突变碱基型。  
(5) Genotype\_\*：亲本碱基型，只保留亲本纯合位点。  
(6) Depth\_\*：亲本碱基型深度。  
(7) Ref\_depth\_\*：各样品支持参考基因组碱基型的reads数。  
(8) Alt\_depth\_\*：各样品支持突变碱基型的reads数。  
(9) Depth\_\*：各样品在该SNP位点的reads支持数。  
(10) SNPindex\_\*：各样品在该SNP位点的SNP频率指数。

图5.2 亲本加两个子代池index的计算

#### 5.2 子代SNP频率分布

为直观反映子代SNP-index在染色体上的分布情况，对SNP-index在染色体上的分布进行作图。默认选择1Mb为窗口，1kb为步长，计算每个窗口中SNP-index的平均值，子代池的SNP-index在全基因组上的分布（manhattan图）如图5.2a，在每条染色体上的分布如图5.2b.

图5.2a 两个子代SNPindex在染色体上的分布

横轴：染色体长度（Mb）；纵轴：SNPindex值；

图中每一个点代表一个SNP位点，其横坐标为SNP位点所在窗口的中间值,纵坐标为其index值,不同染色体间用不同颜色区分；图中黑色的线是SNP-index均值线，需要关注的是两个子代池间均值线差异大的区域。

图5.2b　子代SNPindex在分染色体上的分布

横轴：染色体长度（Mb）；纵轴：SNPindex

图中每一个点代表一个SNP位点，纵坐标为其index值；图中红色的线是SNP-index均值线，需要关注的是某条染色体上两个子代池间均值线差异大的区域。


北京诺禾致源科技股份有限公司


---

  

#### 5.3 子代SNP频率差异分布

为了直观体现两个子代池间差异的区域，计算两个子代SNP-index的差值：△(SNP-index) =SNPindex（S2)-SNPindex(S1)。然后同样选择1Mb为窗口，1kb为步长，计算每个窗口中△(SNP-index)的平均值来反应△(SNP-index)分布；结合群体类型（F2/RIL）和子代混池个数，进行1000次置换检验（Takagi H et.al 2013），选取95%和99%置信水平作为阈值线。置信水平以上的窗口为候选区间，候选区间列表见SNP\_can95.bed.xls ; SNP\_can99.bed.xls;

△(SNP-index)在染色体上的分布（manhattan图）如图5.3a，对△SNP-index在各个染色体上的分布进行作图，如图5.3b。

图5.3a 两个子代△(SNP-index)在染色体上的分布

横轴：染色体长度（Mb）；纵轴：△(SNP-index)

图中每一个点代表一个SNP位点，其横坐标为SNP位点所在窗口的中间值,纵坐标为两个子代池index的差值,不同染色体间用不同颜色区分；图中黑色的线是SNP-index均值线，蓝色的线为95%阈值线，紫色的线为99%阈值线，需要关注的是均值线超过阈值线的区域。

图5.3b 子代各个染色体△(SNP-index)分布

横轴：各个染色体名称及其长度（Mb）；纵轴：△(SNP-index)

图中每一个点代表一个SNP位点，纵坐标为两个子代池index的差值；图中红色的线是SNP-index均值线，绿色的线为95%阈值线，紫色的线为99%阈值线，需要关注的是某条染色体上均值线超过阈值线的区域。

#### 5.4 子代SNP频率差异分析FAQ

**Q1**：SNP-index的计算方法？

**答**：SNP index的计算是对子代池中SNP的一种统计方法，其原理是利用测序reads对每个碱基位点的碱基进行统计，以某一亲本或参考基因组为参考，统计子代池中和亲本或者参考基因组在某一个碱基位点相同或者不相同的reads条数，计算不相同reads条数占总条数的比例，即为该碱基位点的SNP index。对于有两个子代池数据的项目，会过滤掉两个池中SNP-index小于0.3的点。对于过滤后的SNP index会利用滑窗口的方式统计某窗口中所有SNP的SNP-index的平均值作为该窗口的SNP-index，一般默认参数是1Mb的窗口，1kb滑动。按照上述方法分别计算两个子代池的SNP-index，然后在计算两个子代池的SNP-index的差值即为delta SNP-index。

**Q2**：子代SNP频率分布图中的每条线表示什么？

**答**：子代SNP频率分布图中的多条染色体在一起的为整个基因组的SNP频率的曼哈顿分布图，在曼哈顿分布图中横坐标表示染色体，纵坐标表示SNP-index的值，图中的点表示每个SNP对应的位置和SNP-index的值，图中的线表示每个窗口中所有SNP的SNP-index的平均值，是所有SNP-index的趋势的体现。如果挑选出来的SNP数量过少，如是突变材料和野生材料的项目，由于野生型材料和突变材料背景一致，SNP数量很少，故没有窗口的SNP-index的值。

**Q3**：什么情况下可以减少材料的个数？

**答**：如果实验材料为野生材料经诱变产生的突变体和野生材料构建的F2群体或RIL群体时，可以选择野生型亲本和突变子代池两个材料进行分析；如果是两个有性状差异的远缘亲本进行杂交组成的作图群体时，建议准备两个亲本和两个极端性状的子代池进行分析。

**Q4**：BSA性状定位是否可以提供目标区域或者客户指定区域的图片？

**答**：可以，需要客户确定目标区域，此分析为非标准化分析。


北京诺禾致源科技股份有限公司


---

  

#### 5.5 基于SNP标记的目标性状区域定位

为了不忽略掉微效QTL的影响，在全基因组范围内挑选两个子代在SNP-index差异显著的SNP位点，即挑选子代2（极端性状B）SNP-index大于等于0.8，且子代1（极端性状A）SNP-index小于等于0.2 的位点，对于候选的3,833个多态性标记位点，提取ANNOVAR的注释结果,优先挑选upstream, stop loss, stop gain, 非同义突变, 可变剪接等重要的位点所在的基因作为候选基因，候选基因列表请见：candidate.snp\_gene.xls。

表5.4 候选多态性标记位点注释情况

| Category | | Number of SNPs |
| --- | --- | --- |
| Upstream | | 34 |
| Exonic | UTR3 | 8 |
|  | UTR5 | 6 |
|  | UTR5;UTR3 | 0 |
|  | Stop gain | 0 |
|  | Stop loss | 0 |
|  | Synonymous | 17 |
|  | Non-synonymous | 13 |
|  | unknowns | 0 |
| Intronic | | 61 |
| Splicing | | 0 |
| Downstream | | 31 |
| upstream/downstream | | 1 |
| Intergenic | | 3,662 |
| Other | | 0 |
| ts | | 2,420 |
| tv | | 1,413 |
| ts/tv | | 1.713 |
| Total | | 3,833 |

SNP检测及注释结果统计详细内容如下：

(1) Category: 类别。  
(2) Upstream：基因上游1 Kb区域。  
(3) Exonic：变异位于外显子区域；  
    UTR5：5‘端非翻译区；UTR3：3‘端非翻译区；UTR5;UTR3：在一个基因的UTR5，同时在另一个基因的UTR3：  
    Stop gain：使基因获得终止密码子的变异；Stop loss：使基因失去终止密码子的变异；  
    synonymous：同义变异：Non-synonymous：非同义突变：  
    unknowns：未注释到的位点。  
(4) Intronic：变异位于内含子区域。  
(5) Splicing：变异位于剪接位点（内含子中靠近外显子/内含子边界的2bp）。  
(6) Downstream：基因下游1 Kb区域。  
(7) Upstream/Downstream: 基因上游1 Kb区域，同时也在另一基因的下游1 Kb区域。  
(8) Intergenic：变异位于基因间区。  
(9) Other：其他类别。  
(10) ts：transitions，转换。  
(11) tv：transversions，颠换。  
(12) ts/tv：转换与颠换的比率。  
(13) Total：SNP位点总数。

#### 5.6 基于SNP标记的目标性状区域定位FAQ

**Q1**：候选区间如何确定？

**答**：根据计算的SNP-index和delta SNP-index，挑选出SNP-index在两个池中差异较大，delta SNP-index在一定置信水平下的窗口即为候选区间。

**Q2**：候选SNP和候选基因如何确定？

**答**：在候选区间中挑选出SNP index在一个池中为1，另一个池中为0的SNP作为原始的候选SNP，根据这些SNP的注释信息，挑选中在外显子上,且是非同义突变,stop gain,stop loss等重要突变的SNP，这些SNP作为候选SNP，这些SNP所在的基因作为候选基因。

**Q3**：候选基因如何验证？

**答**：由于候选基因的功能的验证需要很多的实验去证明，或者找到的基因是新基因，没有相关的功能注释信息，根据测序数据，客户可以首先验证提供的候选基因中的候选SNP是否是正确的，然后在对每个候选基因进行其他的生化实验的验证，如荧光定量PCR对表达量进行验证，或者直接利用转基因技术对候选基因进行验证，或者酵母双杂对两种蛋白互作进行，或者利用酵母单杂研究蛋白质和特定DNA序列相互作用等等，验证方法根据物种和候选基因而定。

北京诺禾致源科技股份有限公司


---

  

### 6 子代InDel频率差异分析

#### 6.1 子代InDel频率计算

同计算SNP-index值类似，通过计算InDel-index值来进行性状定位，选择亲本3\_1\_muben作为参考亲本，分别计算两个子代的InDel-index（即InDel的频率）。其中，完全与其相同的:InDel-index为0；完全与其不同的InDel-index为1。为了去除背景噪音、减少测序错误比对错误等造成的影响和提高定位的效率，对InDel和InDel-index进行筛选和过滤，筛选和过滤标准如下：

(1) 对于两个亲本的群体，筛选两个亲本间纯合差异的标记；共挑选出281,024个多态性标记；

(2)子代中 InDel-index 小于0.3，或InDel深度小于7的位点，过滤掉;

(3) 一个子代InDel-index缺失的位点，过滤掉;

经过以上筛选，得到过滤后的117,602个多态性标记位点，部分位点InDelindex信息见表6.1

表6.1 部分位点InDel-index信息

| CHROM | POS | REF | ALT | Genotype\_3\_1\_muben | Depth\_3\_1\_muben | Ref\_depth\_Low\_f1 | Alt\_depth\_Low\_f1 | Depth\_Low\_f1 | InDelindex\_Low\_f1 | Ref\_depth\_High\_f1 | Alt\_depth\_High\_f1 | Depth\_High\_f1 | InDelindex\_High\_f1 |
| --- | --- | --- | --- | --- | --- | --- | --- | --- | --- | --- | --- | --- | --- |
| GWHASIY00000001 | 622130 | GTTTTTTTTT | GTTTTTTTTTT | GTTTTTTTTT | 3 | 4 | 3 | 7 | 0.43 | 3 | 4 | 7 | 0.57 |
| GWHASIY00000001 | 718940 | TGGGGG | TGGGGGG | TGGGGG | 3 | 3 | 6 | 9 | 0.67 | 4 | 3 | 7 | 0.43 |
| GWHASIY00000001 | 1102656 | GA | G | G | 7 | 5 | 8 | 13 | 0.38 | 4 | 13 | 17 | 0.24 |
| GWHASIY00000001 | 1107409 | AAGGAGGAGGAGGAGGAGG | AAGGAGGAGGAGGAGG | AAGGAGGAGGAGGAGGAGG | 5 | 11 | 2 | 13 | 0.15 | 6 | 3 | 9 | 0.33 |
| GWHASIY00000001 | 1107916 | ACC | AC | AC | 8 | 4 | 6 | 10 | 0.40 | 9 | 9 | 18 | 0.50 |

部分位点InDel-index信息详细内容如下：

(1) CHROM：染色体名。  
(2) POS：InDel位点。  
(3) REF：该位点参考基因组碱基型。  
(4) ALT：该位点突变碱基型。  
(5) Genotype\_\*：亲本碱基型，只保留亲本纯合位点。  
(6) Depth\_\*：亲本碱基型深度。  
(7) Ref\_depth\_\*：各样品支持参考基因组碱基型的reads数。  
(8) Alt\_depth\_\*：各样品支持突变碱基型的reads数。  
(9) Depth\_\*：各样品在该InDel位点的reads支持数。  
(10) SNPindex\_\*：各样品在该InDel位点的InDel频率指数。

#### 6.2 子代InDel频率分布

为直观反映子代InDel-index在染色体上的分布情况，对InDel-index在染色体上的分布进行作图。默认选择1Mb为窗口，1kb为步长，计算每个窗口中InDel-index的平均值，子代池的InDel-index在全基因组上的分布（manhattan图）如图6.2a，在每条染色体上的分布如图6.2b.

图6.2a 子代InDel-index在染色体上的分布

横轴：染色体长度（Mb）；纵轴：InDel-index

图中每一个点代表一个InDel位点，其横坐标为InDel位点所在窗口的中间值,纵坐标为其index值,不同染色体间用不同颜色区分；图中黑色的线是InDel-index均值线，需要关注的是两个子代池间均值线差异大的区域。

图6.2b 子代各个染色体InDel-index分布

横轴：各个染色体名称及其长度（Mb）；纵轴：InDel-index

图中每一个点代表一个InDel位点，纵坐标为其index值；图中红色的线是InDel-index均值线，需要关注的是某条染色体上两个子代池间均值线差异大的区域。

北京诺禾致源科技股份有限公司


---

  

#### 6.3 子代InDel频率差异分布

为了直观体现两个子代池间差异的区域，计算两个子代InDel-index的差值：△(InDel-index) =InDelindex（S2)-InDelindex(S1)。然后同样选择1Mb为窗口，1kb为步长，计算每个窗口中△(InDel-index)的平均值来反应△(InDel-index)分布；结合群体类型（F2/RIL）和子代混池个数，进行1000次置换检验（Takagi H et.al 2013），选取95%和99%置信水平作为阈值线。置信水平以上的窗口为候选区间，候选区间列表见InDel\_can95.bed.xls ; InDel\_can99.bed.xls;

△(InDel-index)在染色体上的分布（manhattan图）如图6.3a，对△InDel-index在各个染色体上的分布进行作图，如图6.3b。

图6.3a 两个子代△(InDel-index)在染色体上的分布

横轴：染色体长度（Mb）；纵轴：△(InDel-index)

图中每一个点代表一个InDel位点，其横坐标为InDel位点所在窗口的中间值,纵坐标为两个子代池index的差值,不同染色体间用不同颜色区分；图中黑色的线是InDel-index均值线，蓝色的线为95%阈值线，紫色的线为99%阈值线，需要关注的是均值线超过阈值线的区域。

图6.3b 两个子代△(InDel-index)在分染色体上的分布

横轴：染色体长度（Mb）；纵轴：△(InDel-index)

图中每一个点代表一个InDel位点，纵坐标为两个子代池index的差值；图中红色的线是InDel-index均值线，绿色的线为95%阈值线，紫色的线为99%阈值线，需要关注的是某条染色体上均值线超过阈值线的区域。

#### 6.4 子代InDel频率差异分析FAQ

**Q1**：InDel-index的计算方法？

**答**：InDel-index的计算是对子代池中InDel的一种统计方法，以某一亲本或参考基因组为参考，统计子代池中和亲本或者参考基因组在某位点相同或者不相同的reads条数，计算不相同reads条数占总条数的比例，即为该位点的InDel- index。 对于有两个子代池的项目，会过滤掉两个池中InDel -index小于0.3的点。并利用滑窗口的方式计算窗口中的InDel –index。

**Q2**：子代InDel频率分布图的具体含义是什么？

**答**：子代InDel频率分布图是整个基因组InDel频率的曼哈顿分布图，横坐标表示染色体，纵坐标表示InDel-index的值，图中的点表示每个InDel对应的位置和InDel -index的值，图中黑线表示每个窗口中InDel -index的平均值，是所有InDel频率的趋势体现。

**Q3**：分析子代InDel-index的作用？

**答**： ①在SNP标记的基础上进行InDel标记，可以增加标记密度；
②通过InDel将SNP位点连成区间，即合并候选区间；
③筛选与表型差异相关的InDel标记。

北京诺禾致源科技股份有限公司


---

  

### 7 子代SNP和InDel频率差异分析

#### 7.1 子代SNP和InDel频率合并后的分布

与SNP计算一样，通过计算All-index值来进行性状定位，选择亲本3\_1\_muben作为参考亲本，分别计算两个子代的All-index（即SNP或InDel的频率）。其中，完全与其相同的:All-index为0；完全与其不同的All-index为1。为了去除背景噪音、减少测序错误比对错误等造成的影响和提高定位的效率，对All和All-index进行筛选和过滤，筛选和过滤标准如下：

(1) 对于两个亲本的群体，筛选两个亲本间纯合差异的标记；共挑选出2,794,990个多态性标记；

(2) 子代中 All-index 小于0.3，或All深度小于7的位点，过滤掉;

(3) 一个子代All-index缺失的位点，过滤掉;

经过以上筛选，得到过滤后的1,602,850个多态性标记位点，部分位点All-index信息见表5.1

| CHROM | POS | REF | ALT | Genotype\_3\_1\_muben | Depth\_3\_1\_muben | Ref\_depth\_Low\_f1 | Alt\_depth\_Low\_f1 | Depth\_Low\_f1 | SNPindex\_Low\_f1 | Ref\_depth\_High\_f1 | Alt\_depth\_High\_f1 | Depth\_High\_f1 | SNPindex\_High\_f1 |
| --- | --- | --- | --- | --- | --- | --- | --- | --- | --- | --- | --- | --- | --- |
| GWHASIY00000001 | 358165 | T | A | T | 9 | 7 | 4 | 11 | 0.36 | 7 | 7 | 14 | 0.50 |
| GWHASIY00000001 | 358253 | G | T | G | 6 | 7 | 3 | 10 | 0.30 | 8 | 5 | 13 | 0.38 |
| GWHASIY00000001 | 359040 | T | G | T | 4 | 3 | 4 | 7 | 0.57 | 5 | 2 | 7 | 0.29 |
| GWHASIY00000001 | 371412 | A | G | A | 6 | 12 | 3 | 15 | 0.20 | 6 | 8 | 14 | 0.57 |
| GWHASIY00000001 | 386723 | G | T | G | 7 | 5 | 3 | 8 | 0.38 | 3 | 4 | 7 | 0.57 |

部分位点All-index信息详细内容如下：

(1) CHROM：染色体名。  
(2) POS：SNP、InDel位点。  
(3) REF：该位点参考基因组碱基型。  
(4) ALT：该位点突变碱基型。  
(5) Genotype\_\*：亲本碱基型，只保留亲本纯合位点。  
(6) Depth\_\*：亲本碱基型深度。  
(7) Ref\_depth\_\*：各样品支持参考基因组碱基型的reads数。  
(8) Alt\_depth\_\*：各样品支持突变碱基型的reads数。  
(9) Depth\_\*：各样品在该SNP、InDel位点的reads支持数。  
(10) SNPindex\_\*：各样品在该SNP、InDel位点的SNP、InDel频率指数。

#### 7.2 子代SNP和InDel频率合并后的分布

图7.2a 子代Allindex在染色体上的分布

横轴：染色体长度（Mb）；纵轴：Allindex

图中每一个点代表一个SNP或InDel位点，其横坐标为SNP或InDel位点所在窗口的中间值,纵坐标为其All-index值,不同染色体间用不同颜色区分；图中黑色的线是All-index均值线，需要关注的是两个子代池间均值线差异大的区域。

图7.2b 子代Allindex在分染色体上的分布

横轴：染色体长度（Mb）；纵轴：Allindex

图中每一个点代表一个SNP或InDel位点，纵坐标为其All-index值；图中红色的线是All-index均值线，需要关注的是某条染色体上两个子代池间均值线差异大的区域。


北京诺禾致源科技股份有限公司


---

  

#### 7.3 子代Allindex差异分布

为了直观体现两个子代池间差异的区域，计算两个子代All-index的差值：△(All-index) =Allindex（S2)-Allindex(S1)。然后同样选择1Mb为窗口，1kb为步长，计算每个窗口中△(All-index)的平均值来反应△(All-index)分布；结合群体类型（F2/RIL）和子代混池个数，进行1000次置换检验（Takagi H et.al 2013），选取95%和99%置信水平作为阈值线。置信水平以上的窗口为候选区间，候选区间列表见All\_can95.bed.xls ; All\_can99.bed.xls;

△(All-index)在染色体上的分布（manhattan图）如图7.3a，对△All-index在各个染色体上的分布进行作图，如图7.3b。

图7.3a 两个子代delta\_All-index在染色体上的分布

横轴：染色体长度（Mb）；纵轴：△(All-index)

图中每一个点代表一个SNP或InDel位点，其横坐标为SNP或InDel位点所在窗口的中间值,纵坐标为两个子代池All-index的差值,不同染色体间用不同颜色区分；图中黑色的线是All-index均值线，蓝色的线为95%阈值线，紫色的线为99%阈值线，需要关注的是均值线超过阈值线的区域。

图7.3b 子代各个染色体△(All-index)分布

横轴：各个染色体名称及其长度（Mb）；纵轴：△(All-index)

图中每一个点代表一个SNP或InDel位点，纵坐标为两个子代池All-index的差值；图中红色的线是All-index均值线，绿色的线为95%阈值线，紫色的线为99%阈值线，需要关注的是某条染色体上均值线超过阈值线的区域。

北京诺禾致源科技股份有限公司


---

  

#### 7.4 基于SNP和InDel标记的目标性状区域定位

为了不忽略掉微效QTL的影响，在全基因组范围内挑选两个子代在SNP-index或InDel-index差异显著的SNP或InDel位点，即挑选子代2（极端性状B）SNP-index或InDel-index大于等于0.8，且子代1（极端性状A）SNP-index或InDel-index小于等于0.2的位点，对于候选的4,485个多态性标记位点，提取ANNOVAR的注释结果,优先挑选upstream, stop loss, stop gain, 非同义突变, 可变剪接等重要突变的位点所在的基因作为候选基因，候选基因列表请见：candidate.snp\_gene.xls ; candidate.InDel\_gene.xls。

表7.4a 候选SNP多态性标记位点注释情况

| Category | | Number of SNPs |
| --- | --- | --- |
| Upstream | | 34 |
| Exonic | UTR3 | 8 |
|  | UTR5 | 6 |
|  | UTR5;UTR3 | 0 |
|  | Stop gain | 0 |
|  | Stop loss | 0 |
|  | Synonymous | 17 |
|  | Non-synonymous | 13 |
|  | unknowns | 0 |
| Intronic | | 61 |
| Splicing | | 0 |
| Downstream | | 31 |
| upstream/downstream | | 1 |
| Intergenic | | 3,662 |
| Other | | 0 |
| ts | | 2,420 |
| tv | | 1,413 |
| ts/tv | | 1.713 |
| Total | | 3,833 |

SNP检测及注释结果统计详细内容如下：

(1) Category: 类别。  
(2) Upstream：基因上游1 Kb区域。  
(3) Exonic：变异位于外显子区域；  
    UTR5：5‘端非翻译区；UTR3：3‘端非翻译区；UTR5;UTR3：在一个基因的UTR5，同时在另一个基因的UTR3：  
    Stop gain：使基因获得终止密码子的变异；Stop loss：使基因失去终止密码子的变异；  
    synonymous：同义变异：Non-synonymous：非同义突变：  
    unknowns：未注释到的位点。  
(4) Intronic：变异位于内含子区域。  
(5) Splicing：变异位于剪接位点（内含子中靠近外显子/内含子边界的2bp）。  
(6) Downstream：基因下游1 Kb区域。  
(7) Upstream/Downstream: 基因上游1 Kb区域，同时也在另一基因的下游1 Kb区域。  
(8) Intergenic：变异位于基因间区。  
(9) Other：其他类别。  
(10) ts：transitions，转换。  
(11) tv：transversions，颠换。  
(12) ts/tv：转换与颠换的比率。  
(13) Total：SNP位点总数。

表7.4b 候选InDel位点注释情况

| Category | | Number of Indels |
| --- | --- | --- |
| Upstream | | 3 |
| Exonic | UTR3 | 0 |
|  | UTR5 | 1 |
|  | UTR5;UTR3 | 0 |
|  | Stop gain | 0 |
|  | Stop loss | 0 |
|  | Frameshift deletion | 0 |
|  | Frameshift insertion | 1 |
|  | Non-frameshift deletion | 0 |
|  | Non-frameshift insertion | 0 |
|  | unknowns | 0 |
| Intronic | | 6 |
| Splicing | | 0 |
| Downstream | | 3 |
| upstream/downstream | | 0 |
| Intergenic | | 114 |
| Other | | 524 |
| Insertion | | 286 |
| Deletion | | 366 |
| Total | | 652 |

候选InDel位点注释情况详细内容如下：

(1) Category: 类别。  
(2) Upstream：基因上游1 Kb区域。  
(3) Exonic：变异位于外显子区域；  
    UTR5：5‘端非翻译区；UTR3：3‘端非翻译区；UTR5;UTR3；在一个基因的UTR5，同时在另一个基因的UTR3；  
    Stop gain：使基因获得终止密码子的变异；Stop loss：使基因失去终止密码子的变异；  
    Frameshift deletion：移码缺失；Frameshift insertion：移码插入；  
    Non-frameshift deletion：非移码缺失；Non-frameshift insertion：非移码插入；  
    unknowns：未注释到的位点。  
(4) Intronic：变异位于内含子区域。  
(5) Splicing：变异位于剪接位点（内含子中靠近外显子/内含子边界的2bp）。  
(6) Downstream：基因下游1 Kb区域。  
(7) Upstream/Downstream: 基因上游1 Kb区域，同时也在另一基因的下游1 Kb区域。  
(8) Intergenic：变异位于基因间区。  
(9) Other：其他类别。  
(10) Insertion：插入。  
(11) Deletion：缺失。  
(12) Total：InDel位点总数。

#### 7.5 目标性状的候选区间如何确定？

**Q1**：All-index的计算方法？

**答**：根据计算的All-index和△(All-index)，挑选出All-index在两子代池中差异较大，并且△(All-index)在一定置信水平下，大于阈值的窗口作为候选区间。

**Q2**：候选SNP、InDel和候选基因如何确定？

**答**：在整个基因组范围内挑选候选SNP和InDel，挑选子代池中All-index小于等于0.2的位点或者挑选子代池中All-index大于等于0.8的位点作为SNP、 InDel候选位点。根据候选位点的注释信息，优先挑选upstream, stop loss, stop gain, 非同义突变, 可变剪接位点, 移码突变等重要突变位点，这些位点所在的基因作为候选基因。

北京诺禾致源科技股份有限公司


---

  

## 四、附录


### 1　软件列表


## 动植物重测序分析软件介绍

| Analysis | Software | Version | Remarks |
| 序列比对 | BWA | 0.7.10 | 与参考基因组进行比对 |
| SAM和BAM文件转化 | SAMtools | 0.1.19 |  |
| SNP检测 | GATK | 3.8 | 群体SNPcalling |
| SNP注释 | ANNOVAR | 2013Aug23 | SNP根据基因位置注释 |

软件列表：PDF


### 2　Methods英文版

Methods英文版：PDF

### 3 动植物重测序名词解释

动植物重测序名词解释：PDF

### 4　结果文件使用说明

| 4.1 结果文件解析说明： | | |
| --- | --- | --- |
| \*.tar.gz形式的压缩文件： | Unix/Linux/Mac用户 | 使用tar -zxvf \*.tar.gz命令 |
|  | Windows用户 | 使用解压缩软件如WinRAR、7-Zip等 |
| \*.gz形式的压缩文件： | Unix/Linux/Mac用户 | 使用命令gzip –d \*.gz命令 |
|  | Windows用户 | 使用解压缩软件如WinRAR、7-Zip等 |
| \*.zip形式的压缩文件： | Unix/Linux/Mac用户 | 使用命令unzip \*.zip命令 |
|  | Windows用户 | 使用解压缩软件如WinRAR、7-Zip等 |

更多解压命令可参见网络资料：http://www.php100.com/html/webkaifa/Linux/2009/1213/3652.html。

  

| 4.2 结果文件查看说明: | | |
| --- | --- | --- |
| \*.fasta | 序列文件，fasta格式，一般为基因序列或者基因组序列。因文件一般较大，打开较为困难，为您提供了sampled\*.fasta文件（部分\*.fasta中的序列），方便您查看文件格式。 | unix/Linux/Mac用户使用 less 或 more 命令查看； |
|  |  | windows使用高级文本编辑器Editplus/Notepad++等查看。 |
| \*.fq/fastq | 序列文件，fastq格式，一般为reads序列；因文件一般较大，打开较为困难。为您提供了sampled\*.fastq文件（部分\*.fastq中的序列），方便您查看文件格式。 | unix/Linux/Mac用户使用 less 或 more 命令查看； |
|  |  | windows用户使用高级文本编辑器Editplus/Notepad++等查看。 |
| \*.vcf | 用来描述SNP或InDel等变异信息的文本文件，内含每个位点的深度，碱基质量值，比对质量值等信息；因文件一般较大，打开较为困难。vcf的文件说明请查看结果文件的Read Me，方便您查看文件格式。 | unix/Linux/Mac用户使用 less 或 more 命令查看； |
|  |  | windows用户使用高级文本编辑器Editplus/Notepad++等查看。 |
| \*.xls,\*.txt | 结果数据表格文件，文件以制表符（Tab）分隔。 | unix/Linux/Mac用户使用 less 或 more 命令查看； |
|  |  | windows用户使用高级文本编辑器Editplus/Notepad++ 等，也可以用Microsoft Excel打开。 |
| \*.png | 结果图像文件，位图，无损压缩。 | unix/Linux/Mac用户使用display命令打开。 |
|  |  | windows用户可以使用图片浏览器打开，如photoshop等。 |
| \*.pdf | 结果图像文件，矢量图，可以放大和缩小而不失真，方便用户查看和编辑处理，可使用Adobe Illustrator进行图片编辑，用于文章发表等。 | unix/Linux用户使用evince命令打开。 |
|  |  | windows/Mac用户可以使用Adobe Reader/福昕阅读器/网页浏览器等打开。 |

  

| 4.3 结果文件内容说明 [共分为7个文件夹。6号文件夹包含不同标记的index分析结果和图片，7号中是候选位点和候选基因的结果文件，6和7这两部分结果是BSA的核心结果，建议老师先行进行查看此结果文件]；结果文件的具体内容请查看结果文件内容说明PDF版：结果文件列表.pdf | | | | | |
| --- | --- | --- | --- | --- | --- |
| 结果文件列表 对应结题报告 | | | | | |  |
| results | 1.OriginalData （测序数据格式说明） | 1.OriginalData.README.txt | | 说明文档 | 1 |
 example.fq.txt | | 数据示例 | 1 | 2.QualityControl （测序数据质控结果） | 2.1.QualityDistribution 测序质量分布结果 | 2.1.QualityDistribution.README.txt | 说明文档 | 2.1 | \*.QM.png | 测序质量分布图 | 2.1 | 2.ErrorRate 测序错误率分布结果 | 2.2.ErrorRate.README.txt | 说明文档 | 2.2 | \*.Error.png | 测序错误率分布图 | 2.2 | 2.3.ReadsClassification 测序数据过滤分布结果 | 2.3.ReadsClassification.README.txt | 说明文档 | 2.3 | \*.raw\_reads\_classification.png | 原始数据组成分布图 | 2.3 | 2.4.CleanData\_Qcsummary 测序数据质控统计结果 | 2.4.CleanData\_QCsummary.README.txt | 说明文档 | 2.4 | CleanData\_QCsummary.xls | 质控数据汇总表 | 2.4 | 3.Mapping 样本与参考基因组的比对统计结果 | 3.1.Reference 参考基因组基本信息统计 | 3.1.Reference.README.txt | 说明文档 | 3.1 | genome\_stat.xls | 参考基因组统计结果 | 3.1 | \*.fa/\*.fa.fai | 参考基因组序列及索引 | 3.1 | \*.gff3 | 参考基因组对应的注释文件 | 3.1 | 3.2 MapStat 测序深度及覆盖度统计 | 3.2.MapStat.README.txt | 说明文档 | 3.2 | \*.Sequencing-depth.png/pdf | 测序深度统计图 | 3.2 | Allsample\_alninfo.xls | 测序深度统计表 | 3.2 | 4.SNP\_VarDetect 变异位点、统计、注释结果 | 4.SNP\_VarDetect.README.txt | | 说明文档 | 4.1 | SNP.vcf | | SNP变异vcf文件 | 4.1 | snp.avinput.exonic\_variant\_function | | SNP外显子注释结果 | 4.1 | snp.avinput.variant\_function | | SNP全基因组注释结果 | 4.1 | SNP\_Annotation\_Statistics.xls | | SNP注释统计 | 4.1 | 5.indel\_VarDetect 变异位点、统计、注释结果 | 5.indel\_VarDetect.README.txt | | 说明文档 | 4.1 | Indel.vcf | | InDel变异vcf文件 | 4.1 | indel.avinput.exonic\_variant\_function | | InDel外显子注释结果 | 4.1 | indel.avinput.variant\_function | | InDel全基因组注释结果 | 4.1 | InDel\_Annotation\_Statistics.xls | | InDel注释统计 | 4.1 | 6.SNP\_index\_indel\_index\_analysis 变异位点的index值分析 | 6.SNPindex\_analysis.README.txt | | 说明文档 | 5.1 | 6.1.SNPindex\_analysis SNP的index分析结果 | S1.SNP\_index.manhattan.pdf/png | 子代池1的index manhattan图 | 5.2 | S2.SNP\_index.manhattan.pdf/png | 子代池2的index manhattan图 | 5.2 | Delta\_SNPindex.manhattan.pdf/png | Deltaindex的manhattan图 | 5.3 | S1.SNP\_index.pdf/png | 子代池1的index分布图 | 5.2 | S1.SNP\_index.pdf/png | 子代池2的index分布图 | 5.2 | Delta\_SNPindex.png/pdf | delta index分布图 | 5.3 | Delta\_SNPindex.xls | SNPindex差值列表（2个子代池的释放） | -- | Filter\_SNP\_index.xls | 过滤后的SNPindex列表（1个子代池的释放) | 5.1 | candidateSNPindex.xls | 候选SNP位点 | 5.5 | 6.2.indel\_index\_analysis Indel的index分析结果 | 同6.1 | indel的index计算相关结果文件 | 6 ||  |  |  |  |  |  |
 6.3.all\_index\_analysis SNP和Indel合并的index分析结果 | 同6.1 | SNP与indel的index合并计算的结果文件 | 7 ||  |  |  |  |  |  |
 7.Trait\_loci 定位结果 | 7.Trait\_loci.README.txt | | 说明文档 | -- | candidate\_SNP\_gene.xls | | 候选基因列表 | 5.5 | candidateSNP\_Annotation\_Statistics.xls | | 候选SNP位点注释统计 | 5.5 | candidateSNPindex.avinput\_variant\_function | | 候选SNP位点注释 | -- | candidateSNPindex.exonic\_variant\_function | | 候选SNP位点注释 | -- | candidateAllindex\_SNP\* | | allindex分析的候选SNP位点注释及候选基因 | 7.4 | candidateAllindex\_InDel\* | | allindex分析的候选indel位点注释及候选基因 | 7.4 |

### 5 备注

结果文件建议使用Excel、EditPlus或NotePad++等专业文本编辑器打开。Notepad++下载网址：http://baoku.360.cn/soft/show/appid/342 ; EditPlus下载网址：http://baoku.360.cn/soft/show/appid/252 。

如果展示表格底部出现滑动条，则横向滑动可以显示全部内容。

点击Novogene图标或者右下角按钮可以返回首页。

客服电话:400-658-1585

北京诺禾致源科技股份有限公司


---

  

## 五、 参考文献

Li H, Durbin R: Fast and accurate short read alignment with Burrows-Wheeler transform. Bioinformatics 2009, 25(14):1754-1760.

Wang K, Li M, Hakonarson H: ANNOVAR: functional annotation of genetic variants from high-throughput sequencing data. Nucleic acids research 2010, 38(16):e164.

Fiume M, Williams V, Brook A, et al. Savant: genome browser for high-throughput sequencing data[J]. Bioinformatics, 2010, 26(16): 1938-1944.

Fiume M, Smith E J M, Brook A, et al. Savant Genome Browser 2: visualization and analysis for population-scale genomics[J]. Nucleic acids research, 2012, 40(W1): W615-W621.

McKenna A, Hanna M, Banks E, et al. The Genome Analysis Toolkit: a MapReduce framework for analyzing next-generation DNA sequencing data[J]. Genome research, 2010, 20(9): 1297-1303.

Takagi H, Abe A, Yoshida K, et al. QTL‐seq: rapid mapping of quantitative trait loci in rice by whole genome resequencing of DNA from two bulked populations[J]. The Plant Journal, 2013, 74(1): 174-183.
